# Nucleolar reorganization on stress depends on physicochemical changes due to reduced nascent rRNA synthesis

**DOI:** 10.1038/s44319-026-00932-z

**Published:** 2026-09-22

**Authors:** Sinjini Ghosh, Souvik Sen, Aprotim Mazumder

**Affiliations:** Tata Institute of Fundamental Research Hyderabad, 36/P, Gopanpally, Serilingampally Mandal, Hyderabad, 500046 Telangana India

**Keywords:** RNA Biology

## Abstract

Successive maturation of ribosomal subunits occurs through multilayered phase-separated structures of the cell nucleolus. The spatio-functional relationship between transcription of rRNA and nucleolar substructures, and how this adapts to cellular stress remain incompletely understood. In this study, we resolve the sub nucleolar structures using expansion microscopy to reveal ordered structures of fibrillar center (FC) and dense fibrillar component (DFC) domains as nested shells, which are reorganized upon cellular stress like DNA damage or RNA polymerase I (RNAPI) inhibition. Direct visualization of nascent (5’ ETS) and mature (28S or 18S) rRNA suggests that rRNA synthesis is a critical regulator of nucleolar size and organization. Nucleolar reorganization upon stress emerges to be a direct function of nascent rRNA levels. Stress-induced transcription inhibition can remodel the sub-nucleolar compartments to a low mobility state and perturbs the nucleolar pH gradient due to the missing rRNA scaffold and other factors. We show that rather than signaling to mediate rDNA repair, nucleolar reorganization arises naturally from reduced rRNA levels and the resultant biophysical restructuring of the nucleolus under cellular stress.

## Synopsis

Expansion Microscopy shows that DNA or transcriptional stress triggers reorganization of the core-shell architecture of the mammalian nucleolus. This scales with reduction in nascent rRNA levels, and is accompanied by local pH changes and transitions of sub-compartment proteins to a lower mobility state.

- Expansion microscopy reveals the nested core-shell architecture of individual Fibrillar Centres (FC) and Dense Fibrillar Components (DFC) embedded within a larger Granular Component (GC) compartment. Cellular stress reorganizes nucleolar architecture.
- Nascent and mature rRNA show differential localization and responses with DNA damage and transcriptional stress.
- Stress-induced spatial rearrangement of nucleolar sub-compartments scales with nascent rRNA levels.
- Nucleolar reorganization is also accompanied by differential shifts of nucleolar pH, and alteration of nucleolar condensate properties to a state with low translational and rotational mobility of FC proteins like UBF.

## Abstract (web summary)

Expansion Microscopy shows that DNA or transcriptional stress triggers reorganization of the core-shell architecture of the mammalian nucleolus. This scales with reduction in nascent rRNA levels, and is accompanied by local pH changes and transitions of sub-compartment proteins to a lower mobility state.

## Graphical abstract

**Figure:**
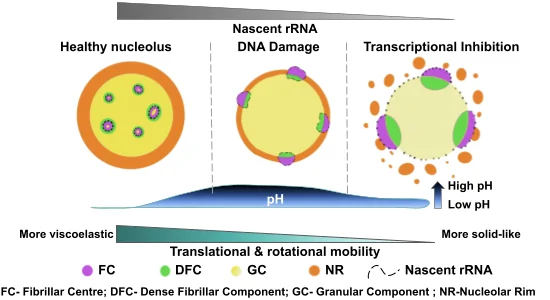

## Introduction

The nucleolus is the central site for ribosome production in eukaryotic cells, and nucleolar function is influenced by its structural organization. A key step in ribosome assembly in human cells begins with RNA polymerase I (RNAPI) synthesizing 47S precursor rRNA transcripts from rDNA gene regions that is processed into three different rRNA (18S, 5.8S, and 28S) (2023; Pederson, 1998). These pre-rRNA molecules undergo multiple steps of folding, modifications and processing and then assemble with ribosomal proteins (RPs), assembly and maturation factors, enabling the initial maturation of the large and small ribosomal subunits (LSU, SSU) of the ribosome. These ribonucleoparticles (LSU and SSU precursors) undergo further maturation in the nucleus, followed by nuclear export to facilitate the final stages of assembly in the cytoplasm to form mature ribosomes (Cerezo et al, 2025). rRNA synthesis is tightly regulated and sensitive to conditions of stress and diseases (Aldrich et al, 2026). Research using diverse tools of electron and light microscopy, molecular biology and biochemistry, has led to a model where different sub-nucleolar domains correspond to different stages in ribosome biogenesis (Hernandez-Verdun, 2006a). rRNA synthesis occurs at the boundary between the fibrillar center (FC) and the dense fibrillar component (DFC) of the nucleolus, while later stages of ribosome maturation happen within the DFC and granular component (GC), respectively (Louvet et al, 2005). Key proteins in this process include the multi-HMG-box chromatin protein Upstream Binding Transcription Factor (UBF) residing in the FC, Fibrillarin (FBL) in the DFC and Nucleolin (NCL) in the GC. Recent studies have also revealed a fourth compartment termed as the nucleolar rim, which houses proteins with higher intrinsic disorder (like Ki-67), but its function is still under investigation (Stenström et al, 2020). Under conditions of DNA damage or transcriptional stress, the three major compartments reorganize into a bipartite arrangement: in this conformation, the innermost FC and DFC compartments shift to the periphery of the nucleolus (Al-Baker et al, 2004). Another structural organization called the ‘nucleolar necklace’ can form under conditions of RNA polymerase II (RNAPII) elongation inhibition (treatment with 5, 6, dichloro-1-*β*-D-ribofuranosylbenzimidazole (DRB)) wherein the FC-DFC modules remain intact while the granular compartment fragments into smaller masses (Louvet et al, 2005). Despite these insights, the detailed spatial arrangement of the nucleolus has only recently been investigated, with studies probing its ultrastructure to gain a better understanding of its tripartite organization (Maiser et al, 2020; Wei et al, 2026; Yao et al, 2019). Studies on the effects of cellular stress or disrupted transcription on nucleolar protein and RNA organization are constrained by diffraction limits of standard optical imaging, which fails to capture the nucleolar substructures, and lack of detection of specific transcripts in species of interest and complex sample preparation in electron microscopy.

**Figure 1:**
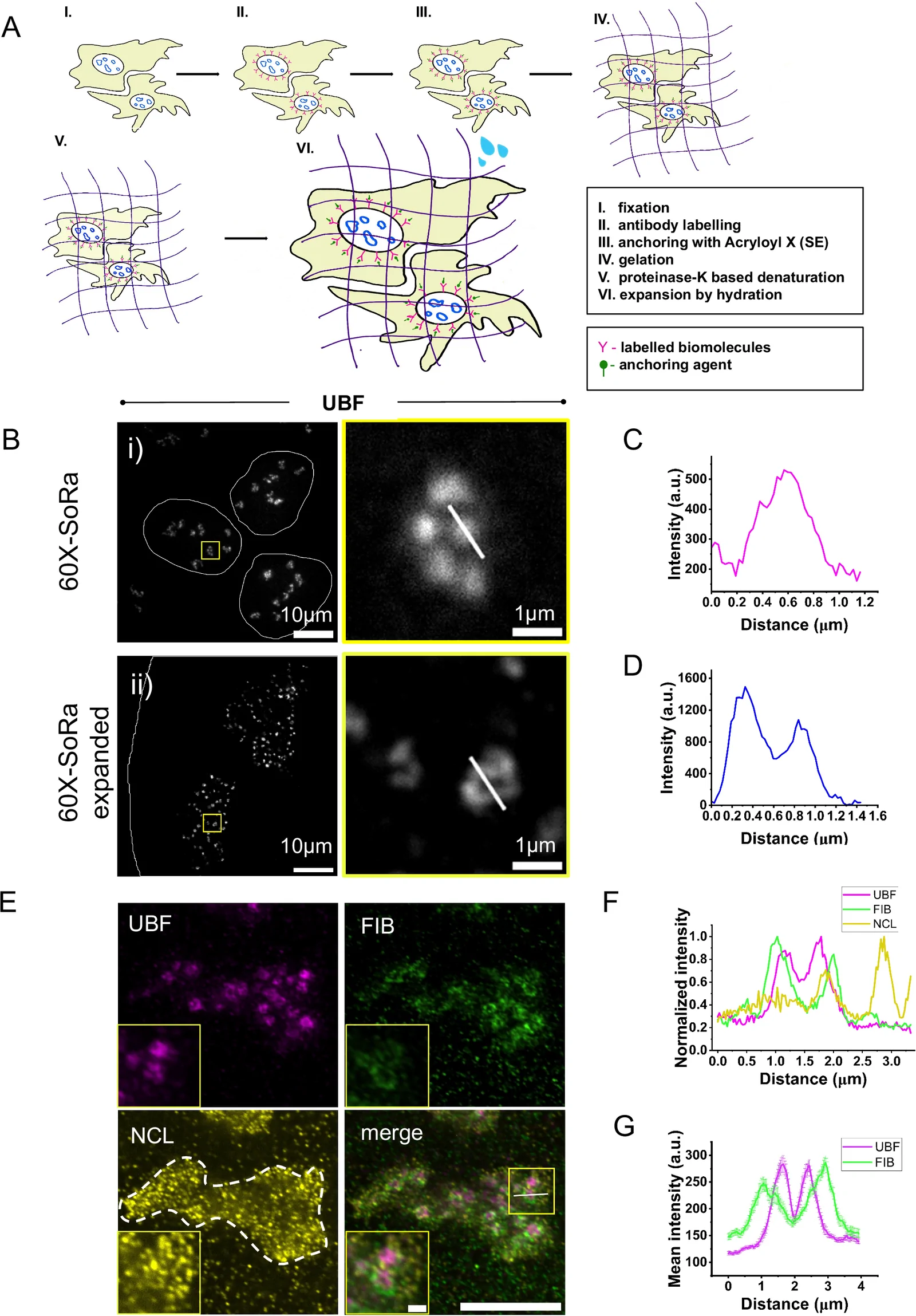
Expansion microscopy shows the ultrastructure of the nucleolus comprising of core-shell sub-compartments. (**A**) Schematic for expansion microscopy. The cells are fixed after culturing on a coverslip (I), and then stained with the antibody of choice (II). The cellular biomolecules are then anchored using Acryloyl-X-SE (III), followed by gelation (IV), denaturation (V), and finally expansion of the gel by hydration. (**B**) Pre-expansion (top panel) and post-expansion (bottom panel) image of U2OS cells stained with an antibody against UBF1 taken in 60X So-Ra, followed by an inset from each of the images (bottom panel). Scale bar is 10 microns for expanded and 1 micron for unexpanded images. (**C**) Line profile for unexpanded (magenta) and (**D**) expanded (blue) UBF foci, marked by white lines in the top and bottom panels of (**B**), respectively. (**E**) Immunofluorescence assay performed on U2OS cells combined with expansion microscopy to mark all three sub-compartments of a single nucleolus: UBF (magenta; Fibrillar centers - FC), FIB (green; Dense fibrillar component - DFC), NCL (yellow; Granular component, GC), with an inset marked in a yellow box highlighting 2–3 UBF-FIB rings. Scale bar is 10 microns for the nucleolus and 1 micron for the inset. (**F**) Single normalized intensity profile marked across all three sub-compartments shown by a white line in the merge image in (**E**). (**G**) The grand mean of intensity profiles from ten FCs. For each FC, four radial lines were measured at 45° intervals and averaged to generate an individual profile. Data were presented as mean ± SEM (Standard error of mean) across the 10 individual FCs from a single experiment. Source data are available online for this figure.

Of the many biological condensates within the cells like stress granules, Cajal bodies, paraspeckles and P-bodies and others, the multiphase nucleolus is perhaps the most apparent and best studied (Sabari, 2020). The intrinsically disordered regions within the nucleolar proteins aid in its condensation into multilayered droplets while RNA binding domains of these proteins and rRNA itself can add to sub-compartment specificity(Carvalho et al, 2021; Lafontaine et al, 2021). The rRNA synthesized at the nucleolar core acts as a scaffold to segregate proteins into different compartments based on their function in the assembly line of making mature rRNA as parts of ribosomes(Riback et al, 2023; Wadsworth et al, 2024). rRNA enables multimodal interactions with proteins, while allowing immiscibility and stability among the sub-compartments due to its polyanionic nature(Berry et al, 2015; Lafontaine et al, 2021). Other than the maturation factors, ATP-driven stripping of biogenesis factors from pre-ribosomal particles, nucleolytic cleavage of the precursor rRNA, and the sequence-encoded surface tensions of the key nucleolar proteins dictate the core-shell architecture of the nested sub-compartments (Aubert et al, 2018). The combined action of K-block and D/E tract enriched nucleolar proteins contributes to the organizational properties of the nucleolus (Rai et al, 2023). The K-blocks create a scaffold of transient multivalent interactions that aid in nucleolar organization, while recent studies speculate that D/E tracts may serve as proton carriers that set up a pH gradient, both contributing to the directional export of processed rRNA (King et al, 2024). Additionally, the gradient of rRNA concentration and length provides enhanced stable differentiation among the sub-compartment owing to its surfactant-like properties (Yamamoto et al, 2023). But how the rRNA levels and localization is different in control versus stressed cells at the level of individual nucleoli remains elusive still. Recent super-resolution methods have changed this landscape (Correll et al, 2024; Maiser et al, 2020; Shan et al, 2023; Yao et al, 2019), and among these methods is expansion microscopy. This method is an emerging set of sample preparation techniques that involve embedding a sample in an expandable polymer and crosslinking its components to this polymer network (Asano et al, 2018; Cho et al, 2018; Gao et al, 2017). The polymer is then expanded in water, causing both the polymer and specimen to expand isotropically. This pulls the biomolecules apart, enabling nanoscale imaging with diffraction-limited optics, thus bypassing the optical limitations of conventional light microscopes (Fig. 1A). This tool has proven effective for analyzing a range of cellular structures across various biological specimens, including cultured cells, unicellular organisms, and tissues (Asano et al, 2018; Cahoon et al, 2017; Cho et al, 2018; Gao et al, 2017; Halpern et al, 2017; Wassie et al, 2019). However, its effectiveness for isotropic expansion and detailed study of a complex multicomponent compartment like the nucleolus is only beginning to get established. Recent studies have employed this method to the study of the chromatin or the nucleolus (Alley, 2025; Pownall et al, 2023), but the biological structure-function relationship in the nucleolus and its stress-dependent reorganization with visualization of specific rRNA species remain unexplored.

In this study, we utilized expansion microscopy in conjunction with super-resolution by optical realignment (So-Ra) imaging to investigate the intricate details of the tripartite nucleolus under normal conditions and with cellular stress. To study stress-induced nucleolar remodeling, we wanted to distinguish between the physical DNA damage from the direct shutdown of nucleolar transcriptional activity. Therefore, we used 4-quinazoline-1-oxide (4-NQO) and *N*-(2-(Dimethylamino) ethyl)-12-oxo-12H-benzo[g]pyrido [1-b] quinazoline-4-carboxamide (BMH21) as distinct molecular handles. 4-NQO is a potent mutagen yielding reactive derivatives which can further generate bulky adducts mimicking UV-radiation-induced damage (Li et al, 2005). On the other hand, BMH21 is a small-molecule quinazoline that disrupts RNAPI-mediated transcriptional elongation by intercalating to the GC-rich regions of rDNA, and triggers degradation of RPA194- the largest catalytic subunit of RNAPI (Jacobs et al, 2022). In addition to super-resolving nucleolar ultrastructure under stress, single-molecule fluorescence in situ hybridization (smFISH) can aid in the visualization of individual RNA molecules within a cell, achieved by using a set of multiple fluorescently labeled oligomers designed against target RNA (Alley et al, 2025; Dhuppar and Mazumder, 2020; Femino et al, 1998; Pasnuri et al, 2023; Raj et al, 2008). We therefore, multiplexed immunofluorescence for nucleolar proteins with smFISH for rRNA in order to examine their relative spatial association. We assessed the correlation between the nascent rRNA synthesis with the spatial reorganization of the nucleolus under genotoxic or transcriptional stress. Such stress is well documented to cause nucleolar reorganization (Al-Baker et al, 2004; Boulon et al, 2010; Daniely et al, 2002a; Ljungman, 2000; Rubbi and Milner, 2003), but we show that nascent rRNA is one of the predictors of nucleolar reorganization. Stress-induced transcriptional downregulation in turn alters the physicochemical properties of the nucleolus in terms of rRNA and pH gradients, resulting in the realignment of the nucleolar sub-compartments under stress.

## Results

### Nucleolus comprises a core-shell (FC-DFC) architecture immersed in a larger condensate of GC, which reorganizes under stress as revealed by expansion microscopy

**Figure EV1:**
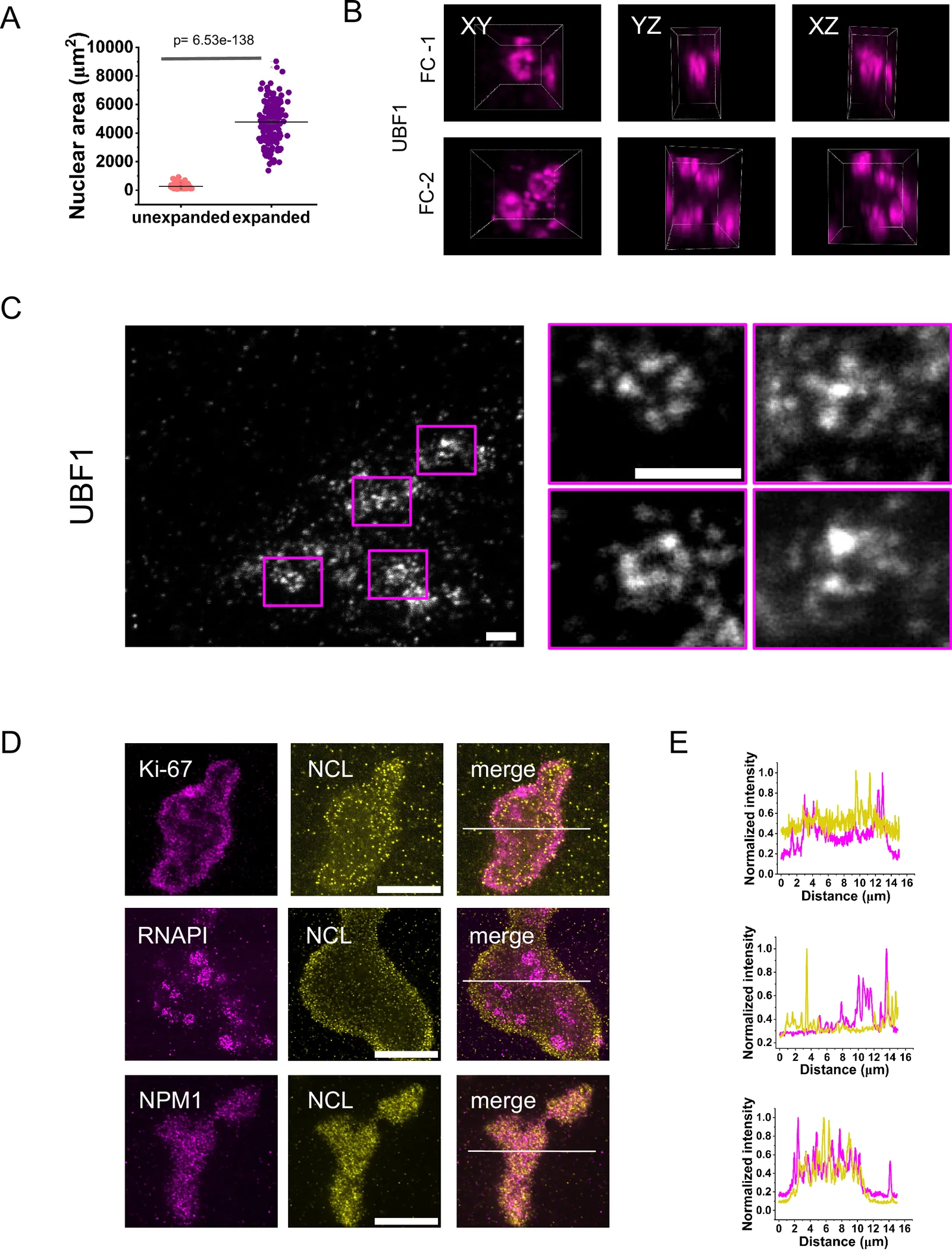
Structural characterization of nucleolar sub-compartments using Expansion Microscopy and STED. (**A**) Graph for nuclear area in unexpanded and expanded nuclei of U2OS cells. Data represents mean ± SEM, *n* = 3 for ≥60 cells, over three independent experiments. (**B**) 3D renders of expanded U2OS cell nucleoli showing three different orthogonal slices (left to right) of XY, YZ, XZ of two FC modules marked by UBF1 (magenta). Scale bar is 5 µm. (**C**) STED images of cells marking UBF1 for a single nucleolus, with 4FC centers marked in magenta boxes, and the insets shown on its right. Scale bar is 0.5 µm. (**D**) U2OS cell nucleoli after expansion, marked with Ki-67, RNAPI, NPM1 (top to bottom respectively, in magenta) co-stained with NPM1 (yellow) in each case, and merge of the two compartments on the rightmost panel. Scale bar is 10 µm. (**E**) Line profile for the merged images from (**D**). Statistical significance calculated using K.S test. Source data are available online for this figure.

The nucleolus is the most distinct phase-separated organelle within the cell. But the detailed structure of the nucleolus, along with different rRNA species have only recently started to be investigated in detail (Maiser et al, 2020; Yao et al, 2019). We used expansion microscopy to visualize the subdomains of the nucleolus (Fig. 1B–D) to gain more insight into nucleolar structure-function relationships. We first standardized the tool using the cell nucleus size as a readout using protein-expansion microscopy (Pro-ExM) (Gao et al, 2017; Tillberg, 2016). We found that the ratio between the area of expanded nuclei to that of unexpanded nuclei resulted in an expansion factor of ~4.18 (Fig. EV1A). This, combined with So-Ra (a SIM-equivalent technology that improves lateral resolution by approximately a factor of 2 (Maiser et al, 2020)), which gives an additional 4X factor in 2D, we can effectively magnify the sample by ~16X. The expansion factor not only serves to enlarge the sample, but also effectively improves resolution since the biomolecules that were in close proximity are now physically apart in space due to the expansion (Cho et al, 2018; Tillberg, 2016; Wassie et al, 2019). The So-Ra provides an additional resolution of the sample by 1.4X (2X with deconvolution), which enables us to gain nanoscale insights into details that were previously underestimated due to the diffraction limit of the microscope. Such expansion tools have been used for the study of chromatin and nucleolar structures recently, giving us additional confidence in our methods (Alley, 2025; Pownall et al, 2023). We used multiplexed labeling of different nucleolar compartment proteins and observed them with So-Ra after expansion (Fig. 1E). Expanded cells showed that both the DFC and FC components marked by fibrillarin (FIB) and upstream binding factor 1(UBF1), respectively, form ring-like structures where the FC ring is enclosed within the DFC ring (Fig. 1F). The rings are oriented in X, Y, and Z directions i.e., they form nested shells as observed in the 3D renders of FC-DFC compartments obtained using IMARIS (Fig. EV1B). We therefore quantified line profiles for the compartment markers over multiple such centers in different nucleoli to get average line profiles to conclude about the spatial relation of the concentric shells of UBF and FIB across cells (Fig. 1G). Considering the expansion factor during calculation, the FIB ringlets have an average diameter of 0.336 ± 0.075 μm, in line with recent studies (Alley, 2025; Maiser et al, 2020). We successfully further resolved the structure of UBF1 as the innermost ringlets (Fig. 1D,G) having an average diameter of 0.204 ± 0.055 μm. While fibrillarin rings have been reported before (Lafontaine et al, 2021; Maiser et al, 2020; Yao et al, 2019), the small size of fibrillar centers makes UBF rings hard to resolve. But using structural illumination microscopy (SIM) imaging, a recent study has reported UBF1 rings, giving us greater confidence about the structures we find (Maiser et al, 2020). We further corroborated the ring-like organization of UBF1 using STED (stimulated emission depletion) microscopy, where UBF1 rings are further spatially resolved into clusters of 5-6 (single cross-sectional plane) beads in a single ring (Fig. EV1C). These multiple factories of nested FC-DFC exist in a bed of GC marked by Nucleolin (NCL) (Fig. 1E). Moreover, we were also able to observe the nucleolar rim (NR) structures by staining for Ki-67 (Fig. EV1D-E), as described in a very recent study (Stenström et al, 2020). We also examine other nucleolar proteins, such as RNAPI and nucleophosmin (NPM), and observe that they, too, reside in the FC and GC compartments, respectively, as expected (Fig. EV1D-E). However, unlike UBF1, RNAPI does not form a ring-like structure, as seen in recent literature (Alley, 2025). Thus, the tool of expansion microscopy offers a unique handle to observe structural details of multiphase condensates of the tripartite nucleolus. A recent study has indeed also reported the use of expansion microscopy to study nucleolar structures (Alley et al, 2025), that lend further credence to our methods. In addition to reporting the structural details under normal homeostasis, we further investigated the roles of nascent and mature rRNA in such organization and its spatial reconfiguration upon stress, quantified over many individual nucleoli, going beyond the visual phenotypes.

**Figure 2:**
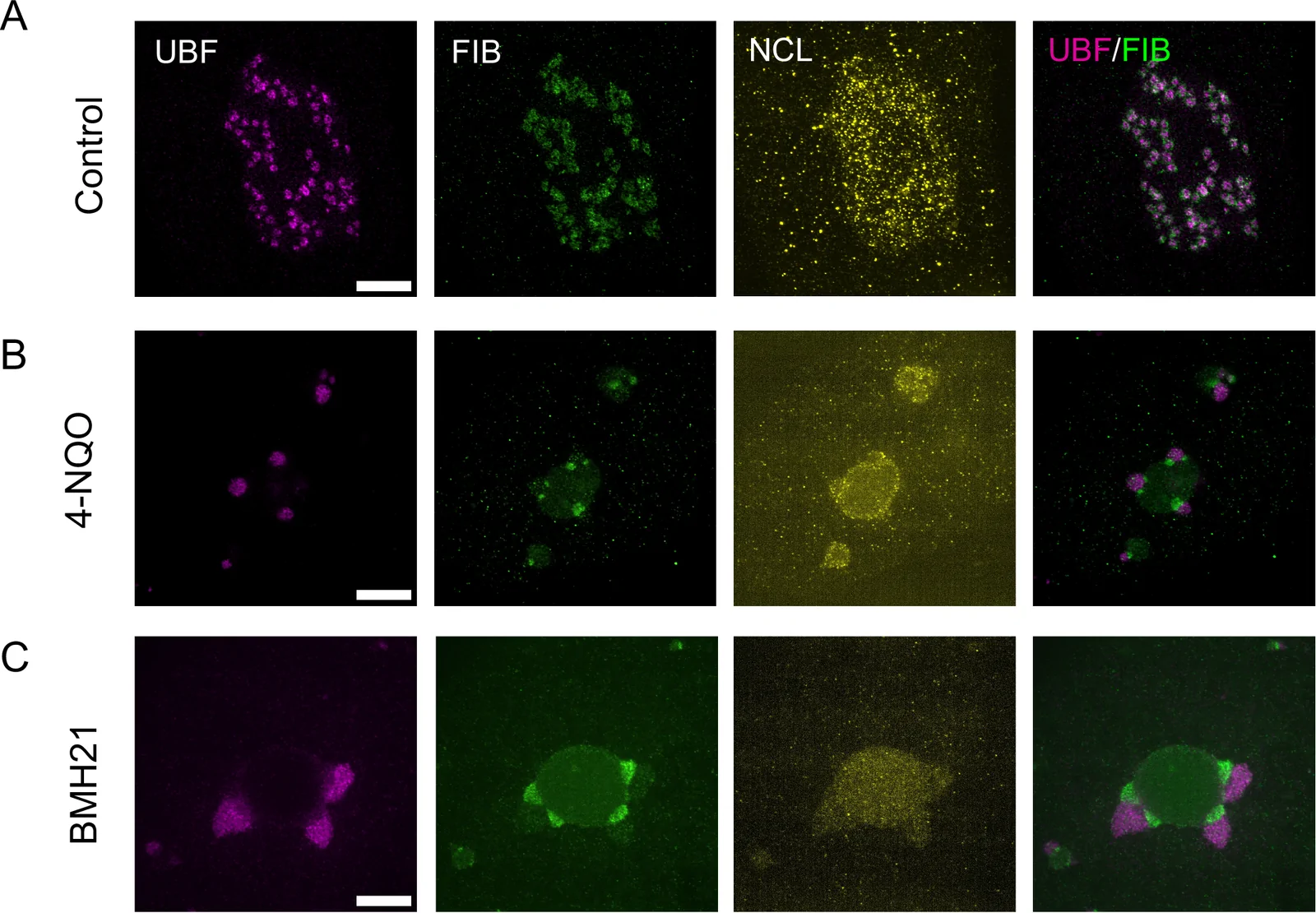
Expansion microscopy shows the reorganization of the nucleolus under genotoxic stress and transcription inhibition. Samples used are U2OS cells immunostained for UBF (magenta), FIB (green), and NCL (yellow). (**A**) Representative expansion microscopy (ExM) image of a single nucleolus in a control cell displaying tripartite organization and multiple fibrillar centers. Nucleolar reorganization following treatment with (**B**) 1 μg/ml 4-NQO for 1 h or (**C**) 2 μM BMH21 for 3 h. Three nucleoli can be seen in (**B**) and one in (**C**). The scale bar is 5 µm and applies to all the images. Due to baseline variations in initial signal intensity with coalescence of UBF foci, contrast and brightness were adjusted to optimize feature visibility in these representative images. No intensity quantification was performed on these images. Reorganization of UBF across the different stress conditions is quantified later in Fig. 5.

**Figure EV2:**
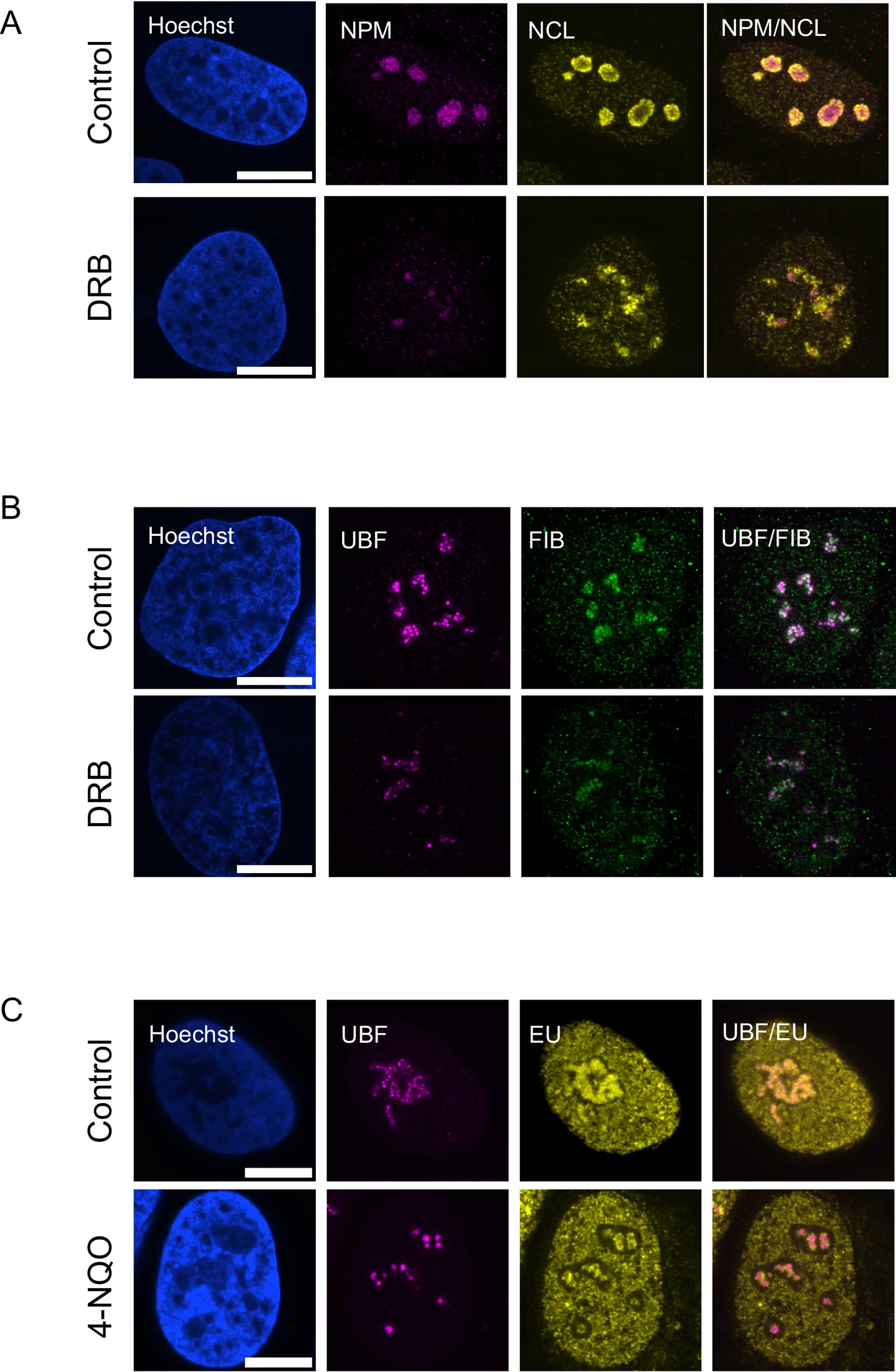
Spatial reorganization of nucleolar proteins upon RNA Polymerase II inhibition by DRB, and rRNA reorganization upon DNA damage by 4-NQO. (**A**) U2OS cells treated with DMSO (control) and DRB at 60 µM, for 1 h, stained with Hoechst (1 µg/ml) and nucleoli marked with NPM (magenta), NCL (yellow), and their merge. (**B**) U2OS cells treated with DMSO (control) and DRB at 60 µM, for 1 h, stained with Hoechst (1 µg/ml) and nucleoli marked with UBF (magenta), FIB (green), and their merge. (**C**) shows cells treated with DMSO (control) and 4-NQO (1 µg/ml) for 1 h with 5-EU pulsed for the last 30 min to detect nascent transcription. It is then stained with Hoechst (1 µg/ml) and nucleoli marked with UBF (magenta), and 5-EU labeled with TAMRA azide (yellow). Scale bar is 10 µm.

**Figure EV3:**
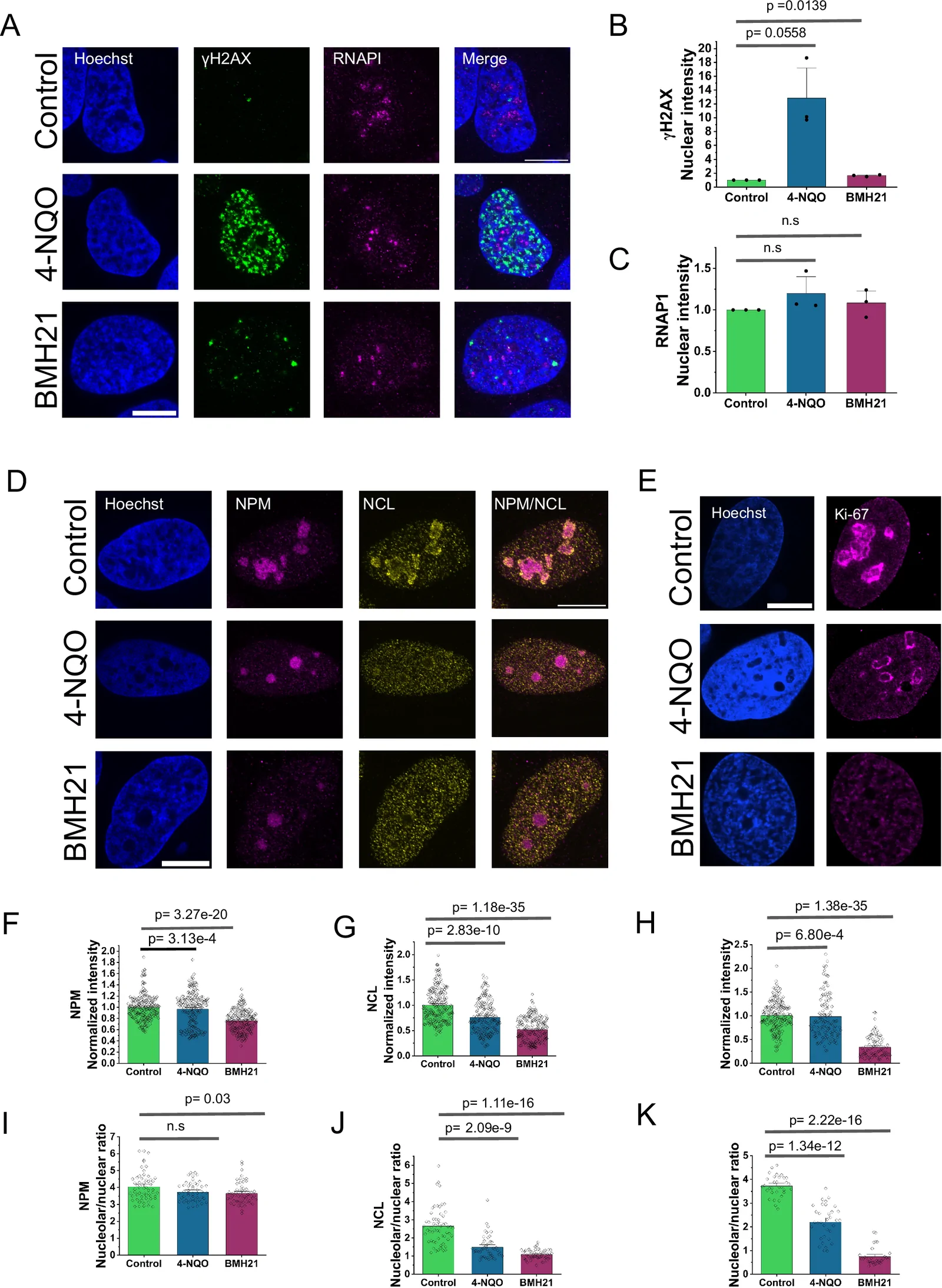
Effects of DNA damage by 4-NQO and transcriptional inhibition by BMH21 on RNA Polymerase I levels and nucleolar protein translocation. U2OS cell nuclei from cells treated with 4-NQO (1 µg/ml) for 1 h and BMH21 (2 µM) for 3 h, compared to control nuclei. (**A**) representative cell nuclei from cells stained with Hoechst (blue, 1 µg/ml), γH2AX (green) and RNAPI (magenta) and their merge. (**B**) Mean nuclear intensity of γH2AX and (**C**) RNAPI plotted from three independent experiments. Each dot represents the average of the individual experiments (each experiment normalized to its control). Statistical significance calculated using a *t*-test. The significance for (**C**) shows higher between control and 4-NQO due to the higher standard deviation among the experiments, despite the mean being significantly different. (**D**) shows nuclei from cells treated with 4-NQO (1 µg/ml) for 1 h and BMH21 (2 µM) for 3 h, compared to control nuclei. Cells are stained with Hoechst (blue, 1 µg/ml), NPM (magenta) and NCL (yellow) and their merge. (**E**) shows nuclei from cells treated with 4-NQO (1 µg/ml) for 1 h and BMH21 (2 µM) for 3 h, compared to control nuclei. Cells are stained with Hoechst (blue, 1 µg/ml) and an antibody against Ki-67 (magenta). (**F**) Normalized nucleolar intensity of NPM, (**G**) NCL and (**H**) Ki-67 plotted. Graph plotted for the ratio between average nucleolar to nuclear (nucleoplasmic) intensity of (**I**) NPM, (**J**) NCL, and (**K**) Ki-67 per cell. Data represents mean ± SEM, with *n* = 58; 48; 57 nuclei (**D**, **F**, **G**, **I**, **J**) and *n* = 32; 34; 33 nuclei (**E**, **H**, **K**) over three independent experiments. Statistical significance calculated using the K.S test. Scale bar is 10 µm. Source data are available online for this figure.

The spatial arrangement of the nested-shell emulsion structure of the nucleolus is a well-known biomarker for cell health, perturbation to which is seen in cancer and neurodegeneration (Carvalho et al, 2021; Dogra and Kriwacki, 2025; Penzo et al, 2019). Therefore, we investigated the impact of DNA damage and transcriptional inhibition on U2OS cells using 4-NQO, a UV-mimetic agent and BMH21 (a RNAPI inhibitor) on nucleolar organization with expansion microscopy (Fig. 2) A DNA damage response (DDR) marker like γH2AX was induced upon 4-NQO treatment as compared to BMH21, while RNAPI reorganizes to form nucleolar caps in both 4-NQO and BMH21 (Fig. EV3A–C). Nucleolar cap formation due to DNA damage or transcriptional inhibition is reported before (Al-Baker et al, 2004; Boulon et al, 2010); but these studies are performed at diffraction-limited resolution and are further somewhat confounded by the fact that many of them use Actinomycin-D used for RNAPI inhibition, that can itself trigger DDR (Mischo et al, 2005). While both Actinomycin-D (Act-D) and the small-molecule BMH21 function as DNA-intercalating transcription inhibitors, their downstream genotoxic profiles diverge significantly. Act-D can induce γH2AX foci (Mischo et al, 2005), reflecting the possible accumulation of DNA double-strand breaks typical of conventional intercalators. Conversely, BMH21-mediated inhibition of RNA polymerase I has been well-documented to suppress transcription and trigger RPA194 degradation without eliciting a γH2AX-positive DNA damage response (Colis et al, 2014) (also see Fig. EV3A–C), highlighting a distinct, non-genotoxic mechanism of RNAPI inhibition and nucleolar stress induction. The stress-induced reorganization revealed that both UBF1 and FIB rings are transformed into coalesced single-phase condensates, which re-arrange at the nucleolar periphery in an inside-out manner with UBF1 now on the outside. The UBF1 protrudes towards the nucleoplasm while FIB faces the inner nucleolar matrix of GC. NCL in this case shows lower intensity within the nucleoli (Fig. 2A–C), suggesting its nucleoplasmic translocation for downstream repair of the damage as reported before (Daniely et al, 2002b; Kobayashi et al, 2012). Because of their larger spatial extent, NR and GC proteins can be resolved well even without expansion. Therefore, we use So-Ra, to study NR protein Ki-67 and GC proteins NPM and NCL under the condition of DNA damage (by 4-NQO) and perturbed nucleolar transcription (by BMH21). NPM shows a moderate decrease in nucleolar intensity, along with lower nucleolar to nucleoplasmic ratios, indicating that it shuttles out into the nucleoplasm upon direct inhibition of rRNA synthesis by BMH21 (Fig. EV3D,F,I). But NCL and Ki-67 showed significant loss of nucleolar pool (Fig. EV3D,E,G,H) and increased nucleoplasmic pool (Fig. EV3J,K) upon DNA damage by 4-NQO and inhibition of nascent rRNA synthesis by BMH21. These indicate that different stressors can affect GC and NR proteins differently, enabling complementary and overlapping stress handling pathways within cells (Scott and Oeffinger, 2016). The coalesced foci of UBF and FIB are parts of the “nucleolar caps” known in earlier studies (van Sluis and McStay, 2017). But the nucleolus reorganizes differently based on how the transcriptional silencing occurs. If the noncoding RNAs (ncRNA) that help in the processing of the rRNA is blocked, the nucleolus segregates, mainly affecting the DFC and GC compartment where the processing take place. Upon treating the cells with 5, 6, dichloro-1-*β*-D-ribofuranosylbenzimidazole (DRB), an RNAPII-mediated transcription elongation inhibitor, we observe that in contrast to RNAPI inhibition, the GC compartment (NPM, NCL) undergoes segregation, forming broken beads of the “nucleolar necklace” known from older studies (Fig. EV2A), while the FCs (UBF1) remain unaffected (Fig. EV2B). Although it is known that transcriptional inhibition and DNA damage leads to nucleolar reorganization (Boulon et al, 2010; Shav-Tal et al, 2005; Haaf and Ward, 1996; van Sluis and McStay, 2017), how the two result in similar phenotypes despite the differences in the mechanisms of stress induction is poorly understood. Recent studies have discussed the synthesis of rRNA, its outward flux and the role of different assembly and maturation factors in this context, suggesting that not just proteins but nucleolar RNA (both rRNA and other noncoding RNAs) is critical for organization. Some studies suggest that the damage to the DNA leads to the nucleolus acting as a stress sensor to allow de-mixing of the condensate and its resident proteins to initiate repair, which leads to nucleolar segregation (Yang et al, 2018). Another idea is that damage to nuclear or nucleolar DNA eventually leads to reduced transcription that disrupts the RNA–protein interaction formed during active rDNA transcription (Korsholm et al, 2019; Korsholm et al, 2020; Larsen and Stucki, 2016). This leads to new interactions among proteins that eventually catalyze protein-rich heterotypic condensates that form the multiphase nucleolar caps (Korsholm et al, 2020). In order to understand the correlation among stress-induced ribosomal biogenesis and nucleolar reorganization better, we next sought to visualize the structural details of rRNA within the cell.

### Nascent rRNA adds on to the nested multilayered structure of the nucleolus, which undergoes reorganization upon stress

**Figure 3:**
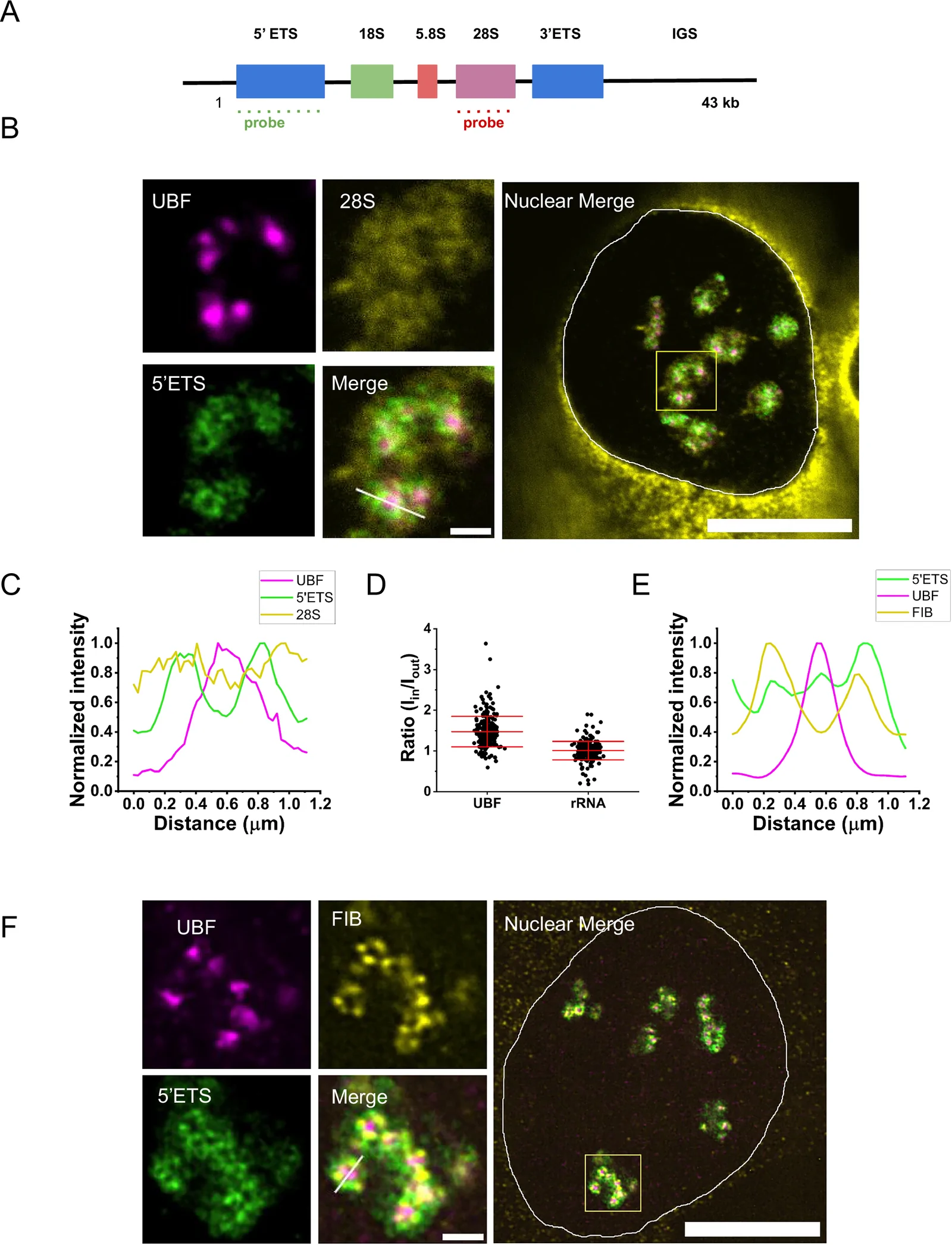
Nascent rRNA adds on to the nested multilayered structure of the nucleolus. (**A**) Schematic for probes used in rRNA FISH. (**B**) Representative images from an immunofluorescence assay on U2OS cells stained for UBF (magenta), and probed for 28S mature rRNA(yellow) on the top panel, nascent 5’ETS rRNA (green) and the merge (bottom panel), with the full nuclear merge on the right showing all the nucleoli in the given nucleus. The scale bar is 1 micron for the nucleoli and 10 microns for the nucleus. (**C**) Normalized intensity profile drawn across the merged inset (marked with a white line), for UBF, 28S rRNA and 5’ETS rRNA. (**D**) Ratio of intensity of UBF and 5’ETS rRNA measured at the center (I_in_) to the periphery (I_out_) of individual ring-shaped FC structures as seen in (**B**). *n* = 245 FCs, over three independent replicates (**E**) Intensity profile of a line drawn across immunofluorescence images U2OS nucleoli shown in (**F**) marked with UBF (magenta). FIB (yellow) in the top panel, 5’ETS (green) and the merge (bottom panel) with the full nuclear merge on the right showing all the nucleoli in the given nucleus. The images used are deconvolved. Scale bar is 1 µm for the nucleoli and 10 µm for the nucleus. Data represents mean ± SEM. Statistical significance calculated using (Kolmogorov–Smirnov) K.S. test. Source data are available online for this figure.

**Figure 4:**
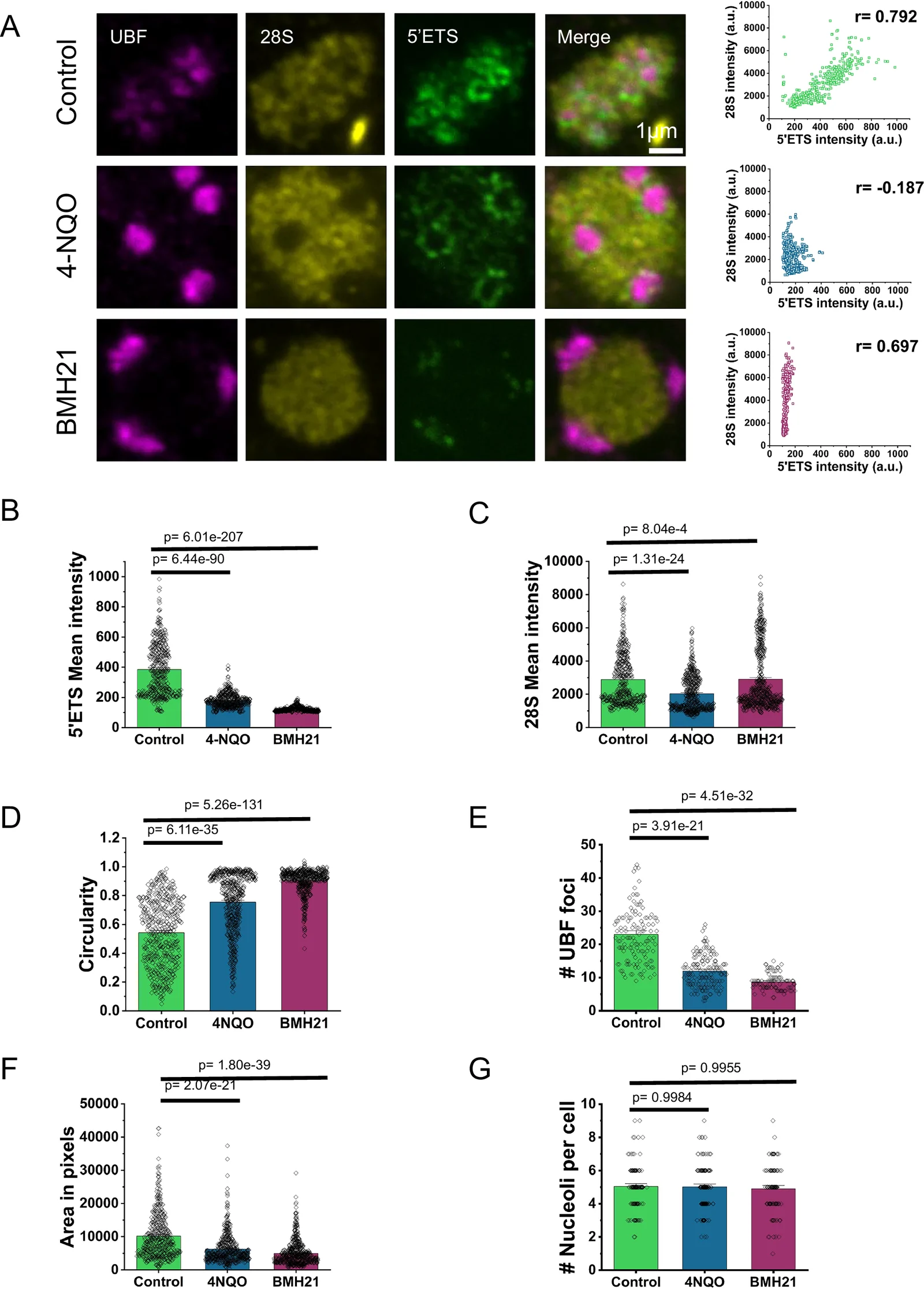
The nucleolus shows stress-induced relocalization of FC-resident protein UBF and changes in levels of nascent rRNA. (**A**) Representative immunofluorescence images of U2OS cell nucleoli stained for UBF (magenta), and probed for 28S rRNA (yellow), 5’ETS (green), and its merge, and compared with cells treated with 1 µg/ml 4NQO for 1 h (middle panel) or 2 µM of BMH21 for 3 h (lower panel) with a correlation study between intensity levels of 28S rRNA and 5’ETS rRNA. Pearson’s correlation coefficient (r) is mentioned on the graphs. The correlations are significant at *p* < 0.0001. (**B**–**G**) Various nucleolar parameters calculated from the experiment in (**A**). (**B**) Mean intensity of 5’ETS and (**C**) Mean intensity of 28S rRNA in individual nucleoli; *n* ≥ 459 nucleoli. (**D**) Circularity of nucleoli, *n* ≥ 451 nucleoli, (**E**) Number of UBF foci, *n* ≥ 90 nucleoli, (**F**) Nucleolar area, *n* ≥ 448 nucleoli, and (**G**) Number of nucleoli per cell (*n* > 100 nucleoli, from two independent replicates). Data from (**B**–**F**) represents mean ± SEM. Statistical significance calculated using the K.S. test. Scale bar is 1 micron. All data except (**G**) are from three independent experiments. Source data are available online for this figure.

**Figure EV4:**
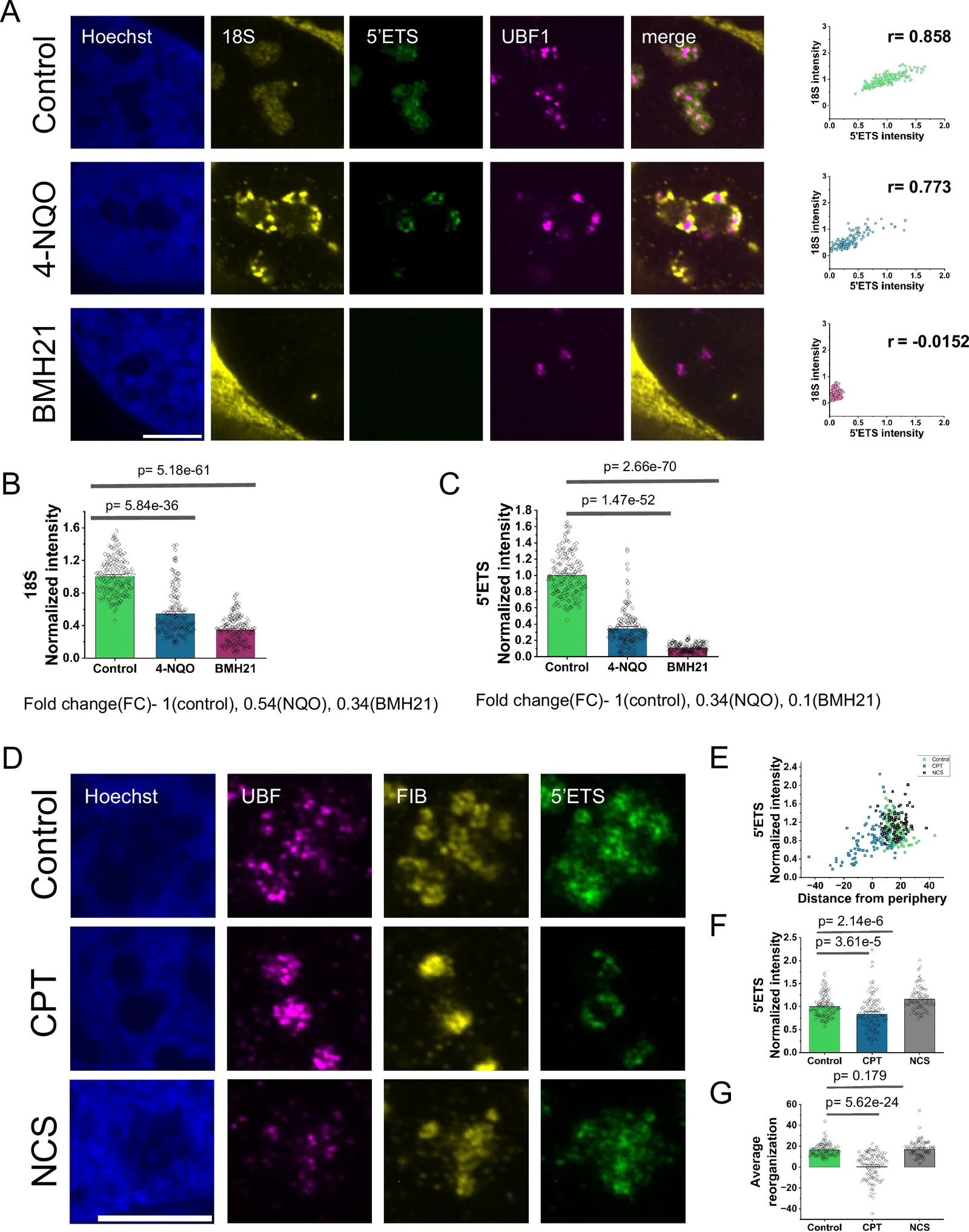
Changes to SSU rRNA levels and nucleolar reorganization in response to different stressing agents. U2OS cell nuclei from cells treated with 4-NQO (1 µg/ml) for 1 h and BMH21 (2 µM) for 3 h, compared to control nuclei. (**A**) representative cell nuclei from cells stained with Hoechst (blue, 1 µg/ml), probed with 18S rRNA(yellow), 5’ETS rRNA(green), and stained with an antibody against UBF1 (magenta) and merged, with a scatter plot for correlation between 18S rRNA and 5’ETS, showing the normalized intensity for each treatment beside it. Pearson’s correlation coefficient (r) is mentioned on the graph. The correlation is significant for control and 4-NQO and non- significant for BMH21 at *p* < 0.0001. (**B**) Normalized intensity of 18S rRNA. (**C**) Normalized intensity of 5’ETS rRNA. Data represents mean ± SEM, with mean labeled for each condition. *n* ≥ 150 (165; 150; 158) nucleoli over three independent experiments. Fold changes (FC) with respect to control is 0.54 for 4-NQO and 0.34 for BMH21 in (**B**), while it is 0.34 for 4-NQO and 0.1 for BMH21 in (**C**). (**D**) Cells treated with camptothecin (CPT, 25 µM, 1 h), and neocarzinostatin (NCS, 1 µg/ml, 1 h) compared to control in DMSO. Cells are stained with Hoechst (blue), UBF1 (magenta), Fibrillarin (yellow) and probed for 5’ETS rRNA (green). (**E**) Mean 5’ETS intensity plotted against the mean distance of the UBF1 foci from the nucleolar periphery (in pixel units). Note how CPT data points show both lower 5’ETS intensity and lower distance values indicative of reorganization, while the NCS data points effectively overlap with the control. (**F**) Normalized 5’ETS intensity from (**E**). (**G**) Average distance from the nucleolar periphery from (**B**). Data represents mean ± SEM. Statistical significance calculated using the K.S. test, where *n* ≥ 95 nucleoli across three independent experiments. While NCS data also turns out to be significantly different in a K.S test, given the high nucleoli numbers, the mean values are quite comparable to the control cells. This is also seen in the scatter plot. Scale bar is 1 µm. Source data are available online for this figure.

rRNA molecules are co-transcribed as the 47S precursor rRNA(pre-rRNA) inside the nucleolus (approximately 13 kb), flanked by the External Transcribed Spacers on both 5’ and 3’ end (5’ETS, 3’ETS), and interspersed by Internal Transcribed Spacer sequences (ITS1 and ITS2) (Aubert et al, 2018). The ETS gets spliced by endonucleases during the earlier steps, while the ITS gets removed in later stages of processing and modifications (Aubert et al, 2018; Vanden Broeck and Klinge, 2024), to finally form the mature 28S, 18S, and 5.8S rRNA. The modified rRNA then diffuses to GC for further processing, assembles with pre-ribosomal subunits, and radially moves out of the nucleolus, to form mature ribosomal subunits (Dogra and Kriwacki, 2025) in the nucleus after the shedding of specific assembly factors (Cerezo et al, 2019). The proteins aiding in this process of rRNA synthesis, processing and ribosomal assembly are organized into the nested multiphase nucleolus. Most of the earlier works have involved visualization of cellular RNA using RNA synthesis analogs like 5-ethynyl-uridine (5-EU), which mark the overall RNA pools in the cell (Jao and Salic, 2008). More recent studies have been investigating the structure of the nucleolus using rRNA sequence-specific probes by in situ hybridization (Quinodoz et al, 2025; Wei et al, 2026; Yao et al, 2019). As rRNA represents the major pool of cellular RNA, EU does show enhanced signals in nucleoli, but it does not discriminate rRNA in different stages of processing. We find that though 5-EU showed some patterned structure inside the nucleoli (Fig. EV2C), but given this limitation, it is perhaps not the best tool to differentiate between nascent and mature rRNA transcripts. Therefore, to understand how the synthesized rRNA resides within the nucleolar structure, we designed probes for both nascent (5’ETS) and mature (28S) rRNA (Fig. 3A) and used smFISH to identify their localization cell nucleoli. Given the high density of rRNA within nucleoli individual rRNA molecules are not distinguished like mRNA previous studies (Dhuppar and Mazumder, 2020; Pasnuri et al, 2023). But the distinct spatial localization of 5’ETS and 28S were clear. Intensities of each species were measured at a single nucleolar level for quantification. We were unable to couple our smFISH protocol with expansion microscopy, and these experiments were performed with only So-Ra imaging, which still offers improved resolutions by a factor of 1.4X over diffraction-limited light microscopy. We observe that the mature rRNA marked by probes against 28S rRNA, are spread across the GC and the cytoplasm (where they are expected as a part of ribosomes), and excluded from the FCs. The FC-DFC boundary contains nascent rRNA which have not undergone splicing and modifications that are salient features of the mature rRNAs (Fig. 3B). In contrast to the 28S mature rRNA signals, nascent rRNA marked by probes against the 5’ETS sequence, on the other hand, formed doughnut-like structures surrounding the FCs (marked by UBF1) (Fig. 3B) as marked by the line profile (Fig. 3C) drawn across the merged image in Fig. 3B. Beyond just visual phenotypes, we quantified these ring-like structures by computing the ratio between the intensities at the center of the UBF1 foci to the outside. For a uniform distribution, this enrichment metric should be 1. This enrichment metric is similar to or less than 1 for 5’ETS, suggesting it to be a hollow ring, while it is more than 1 for UBF1 itself, as it marks the FC (Fig. 3D). UBF1 rings are not apparent in this imaging mode without expansion, and the protein just serves as a proxy for the FC. From our previous expansion microscopy-based experiments, we already know that UBF1 is in itself a 3D-shell-like structure. Therefore, nascent rRNA is synthesized in an elongating bead-like manner on the ringlets of UBF1, forming a nested loop. Co-stained FC-DFC modules (using UBF1 and FIB) along with 5’ETS to understand the spatial localization of the nascent pre-rRNA showed that the pre-rRNA partially overlapped with FIB, seen in the images as well as the line profile (Fig. 3E,F). The partial interspersion of 5’ETS with FIB signals (Fig. 3F) corroborates the longstanding idea that rRNA transcription is initiated at the interface of FC-DFC with co-transcriptional processing, triggering phase separation and rRNA sorting into the subsequent compartments. In-vitro studies have shown how RNA acts as a scaffold for seeding liquid-liquid phase-separated (LLPS) condensates (Rai et al, 2023; Roden and Gladfelter, 2021), and it is known that reducing RNA levels can cause protein orders in condensates to be reversed in in-vitro experiments (Rai et al, 2023). It can also hasten the nucleation of the condensates by reducing the activation energy of the coalescence kinetics (Sabari, 2020; Lafontaine et al, 2021; Wadsworth et al, 2024). It is known that the rRNA aids in the formation of the nested tripartite structure of nucleoli (Farley et al, 2015; van Sluis and McStay, 2017) and direct or indirect inhibition of transcription can lead to nucleolar reorganization, leading to segregation or a reoriented nucleolar cap architecture. So, we directly visualized the rRNA upon global DNA damage and transcriptional stress. We find that inhibition of RNAPI-mediated transcription by BMH21 leads to complete abrogation of nascent rRNA visualized by 5’ETS probes (Fig. 4A,B). Mature and processed rRNA marked by 28S rRNA probes, though significant, show modest changes compared to 5’ETS rRNA (Fig. 4B,C). This is reasonable, because inhibition of transcription does not immediately degrade mature rRNA already synthesized. DNA damage by 4-NQO has a similar but less pronounced impact on the nascent rRNA. Correlations between nascent and mature rRNA levels were strong in control cells, and became markedly weak with cellular stress (Fig. 4A). Cells administered with 4-NQO (1 µg/ml, 1 h) revealed that even though nascent rRNA is not completely abrogated, it still leads to a bipartite rearrangement of FC-DFC as seen under the effects of transcriptional stress. However, the protrusion of UBF1 from the core of the nucleolus under these conditions was not as complete as with BMH21 (Fig. 4A), providing an early hint that nascent rRNA levels may be a critical regulator of nucleolar reorganization. Nascent rRNA, though present in this case, reorganizes to form larger rings (compared to control) with coalesced UBF foci at its center, as compared to active FC ringlets seen in control cells with super-resolution microscopy (Figs. 2A and EV1C). With higher downregulation of rRNA synthesis by BMH21, the residual pre-rRNA migrates to the nucleolar periphery, forming a ring around the nucleolar cap. We also observe that the nucleolar shape becomes more circular concomitant with progressive decline in nascent rRNA levels (Fig. 4D), and reduction in number of UBF foci (Fig. 4E). Although the 28S rRNA originates from the same 47S precursor as 5’ETS and can be used as a proxy for mature rRNA as done in previous studies (Yao et al, 2019, Quinodoz et al, 2025), it is important to note that 28S is a part of the LSU, whereas 5’ETS and 18S are parts of the precursor rRNA that eventually become a part of the SSU. To understand whether stress can differentially affect the maturation kinetics of the SSU (containing 18S) and the LSU (containing 28S), we also probed the cells with probes 18S rRNA and 5’ETS in the same cells to compare the nascent and mature rRNA involved in the same processing pathway (Fig. EV4A–C).

Both 18S and its corresponding upstream spacer, 5’ETS showed progressive decline across treatments of 4-NQO and BMH21, indicating that the small subunit pathway is more sensitive to transcriptional alterations, undergoing rapid quality-controlled degradation when transcription is perturbed. Previous work has shown that upon stress, ribosomal subunits can show differential clearance where 18S containing intermediates are rapidly degraded via nuclear exosome or non-functional rRNA decay (Coria et al, 2025; Ferreira et al, 2020). However, while there is a decrease, the relative fold change for the mature 18S (0.54 for 4NQO, and 0.34 for BMH21, Fig. EV4B) is smaller than for the nascent 5’ ETS (0.34 for 4NQO, and 0.1 for BMH21, Fig. EV4C). Therefore, while fold changes are different for 18S and 28S rRNA, both mature rRNA species (18S or 28S) show more modest changes than nascent rRNA (5’ ETS); but mature rRNA or partially processed RNA fragments in itself is insufficient to maintain the tripartite organization of the nucleolus in line with previous work (Quinodoz et al, 2025) and cannot substitute for the electrostatic scaffolding provided by actively transcribed nascent rRNA. The shape of the nucleolus has been shown to be a marker of nucleolar activity (Farley-Barnes et al, 2018; Hernandez-Verdun, 2006b; Lafita-Navarro and Conacci-Sorrell, 2023). Additionally, nucleolar area gradually decreases with damage and complete abrogation of rRNA synthesis (Fig. 4F), but the overall nucleolar numbers remain the same (Fig. 4G). Despite a reduction in size, the stability of nucleolar counts (Fig. 4G) indicates a functional downshift rather than a structural disintegration. While the structural integrity of the individual nucleolar organizing regions (NORs)-associated nucleolar assembly remains intact, their overall capacity for mature ribosomal production post rRNA synthesis is reduced under stress. Earlier reports suggested that the protein level reorganization in both inhibition of RNAPI-mediated transcription and DNA damage are similar (Franek et al, 2016). The stressors used to cause damage or inhibit transcription, like endonucleases or Actinomycin-D, show a cumulative response of DNA damage and transcription downregulation. Therefore, we try to delineate the two by using BMH21 which shows minimal DDR (Fig. EV3A,B). We show that the spatial localization and levels of nascent rRNA can vary in the case of DNA damage and transcriptional stress, perhaps related to the level of rRNA abrogation. To investigate this point further, we aimed to understand the relation between the spatial reorganization of the compartment-specific proteins and nascent rRNA levels with varying time after DNA damage.

### Cellular rRNA levels and organization scale with the stress-induced spatial rearrangement of nucleolar compartments

**Figure 5:**
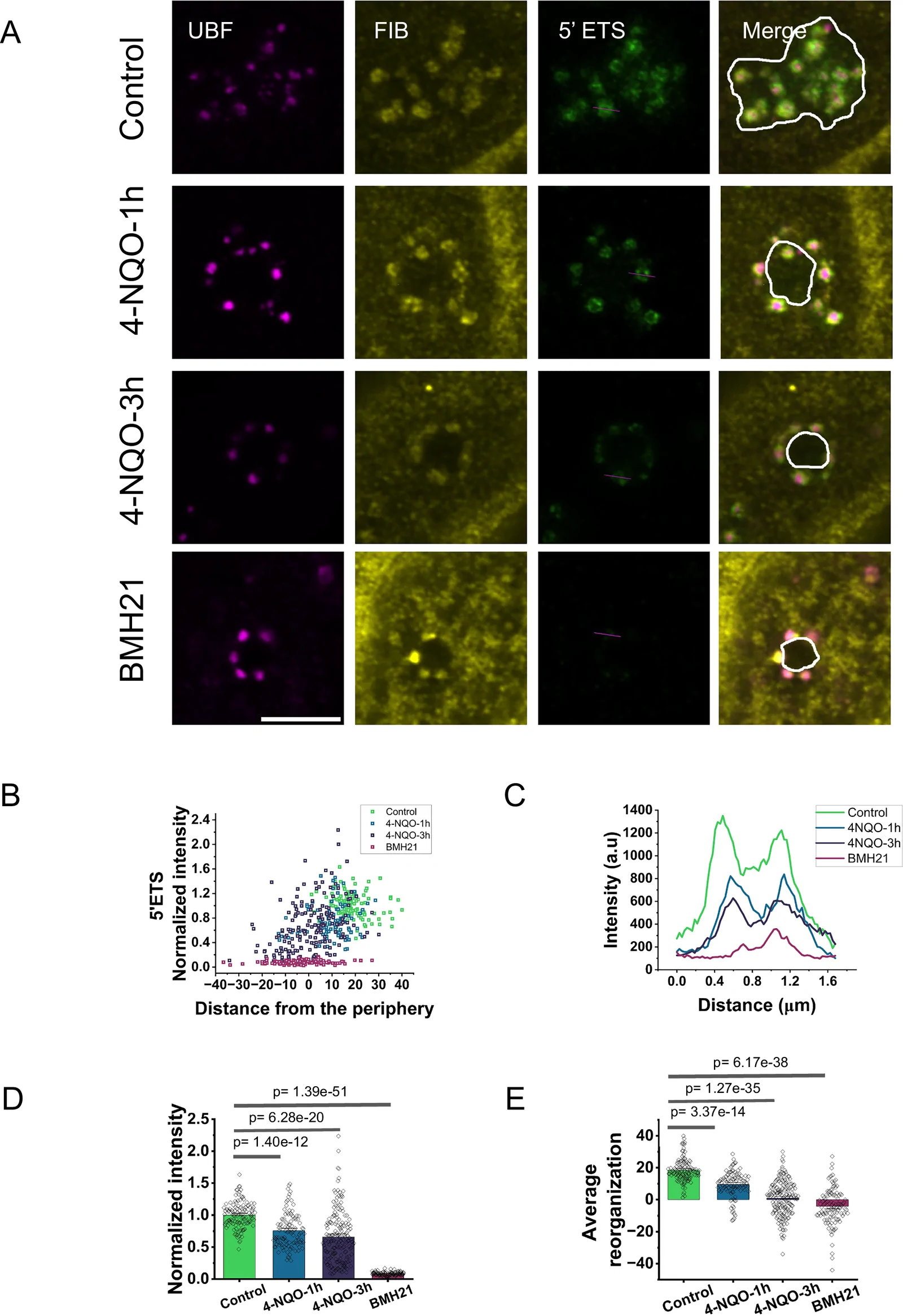
Genotoxic stress leads to a similar nucleolar organization as seen in transcriptional inhibition by BMH21. (**A**) Representative images from immunofluorescence assay of U2OS cell nucleoli stained for UBF(magenta), FIB (yellow), 5’ETS (green), and their merge, and compared to cells treated with 1 µg/ml 4NQO for 1 h, 3 h or 2 µM of BMH21 for 3 h. The white line marks the nucleolar boundary. (**B**) Scatter plot of average distance of UBF foci from the nucleolar periphery (in pixel units) and the rRNA intensity in individual nucleoli. *N* ≥ 95 nucleoli, across three independent experiments. (**C**) Spatial arrangement of rRNA across the images shown in (**A**) with a line profile. (**D**) Normalized 5’ETS (nascent rRNA) intensity from (**B**). (**E**) Average distance from the nucleolar periphery from (**B**). Data represents mean ± SEM. Statistical significance calculated using the K-S test. Scale bar is 5 microns. Source data are available online for this figure.

The cell nucleolus is emerging as a stress sensor within the cells (Yang et al, 2018). It has gained popularity as a potential biomarker for pathological conditions of cancer and neurodegeneration in the past decade. But most of the studies have looked at rRNA using either bulk methods or 5-EU at long-term damage intervals (Korsholm et al, 2020; Szaflarski et al, 2022). These data suggested rRNA synthesis is significantly downregulated along with nucleolar cap formation that leads to migration of the damaged loci to the nucleolar caps, allowing access to repair factors earlier known to be excluded from the nucleolar interior (Franek et al, 2016; van Sluis and McStay, 2017). But how the rRNA localization is actually spatially altered along with reduced synthesis upon DNA damage is not well understood. Therefore, to see if persistent DNA damage can cause further abrogation of rRNA levels, we looked at an additional timepoint of 4-NQO damage (1 µg/ml, for 3 h), comparing it to cells administered with the same dose but a lower time of DNA damage (1 h) and transcriptional stress by BMH21 (as discussed in the previous section). We found that the increased exposure to 4-NQO led to further migration of the FC-DFC modules towards the nucleolar periphery (Fig. 5A). The nascent rRNA is decorated at the nucleolar periphery with fewer rings of nascent rRNA enveloping the FC-DFC cap formed at the periphery, suggesting that increased damage time leads to further downregulation of nascent rRNA and more fusion of the protein condensates forming coalesced foci at the nucleolar perimeter. To quantify the nucleolar reorganization, we designed a metric where stress-induced reorganization of UBF is quantified and correlated to the nascent rRNA levels. The nucleolar perimeter is marked, and the nascent rRNA intensity within the nucleoli is computed. The FC centers marked by UBF1 in a nucleolus are segmented, and the centroid is calculated for each. The shortest distance between the individual UBF1 foci and the nucleolar periphery is calculated. Distance of each such UBF focus is calculated from the nucleolar perimeter, with positive values indicating foci within the nucleolar perimeter and negative values indicating foci whose centroid lay outside the nucleolar periphery, in effect indicating extrusion of the foci. The average distance of the UBF1 foci from the nucleolar periphery is then correlated to the changes in the intensity of the nascent rRNA levels, in a given nucleolus. The nascent rRNA show graded reduction in across control, 4NQO-1h, 4-NQO-3h, and BMH21 respectively, and it positively scales with the movement of FC compartments progressively towards the periphery (Fig. 5B,D,E). We also probed for nascent rRNA (5’ETS) multiplexed with DFC (Fibrillarin/FIB) and FC (UBF) proteins with other DNA-damaging agents to score for the nucleolar reorganization and see if such reorganization can be a general feature or specific to 4NQO. We used other chemical agents that can result in strand-breaks: Neocarzinostatin (NCS, 1 µg/ml, 1 h)—a double-strand break inducer, and Camptothecin (CPT, 25 µM, 1 h)— Topoisomerase -I inhibitor, eventually forming single and double-strand breaks (Fig. EV4D,E). Though reorganization will depend on the dose and time-point of a chemical agent used, we find that with the doses used, CPT reduced both nascent rRNA levels and caused nucleolar reorganization, but NCS, the rRNA intensity is unperturbed, the reorganization is also less pronounced (Fig. EV4E–G). This further highlights the fact that rRNA intensity and localization scale with the reorganization of the nucleolar compartments, like FC and DFC. UBF1 and FIB coalesce into larger condensates upon decreasing rRNA synthesis across most of the stressors. This is possibly due to greater homo and heterotypic protein–protein interaction in the absence of synthesizing rRNA as compared to RNA–protein interactions in actively transcribing populations of cells (Lafontaine et al, 2021; Zhu, 2019). Therefore, we next probed into the dynamic biophysical properties of the nucleolar sub-compartments that regulate the nucleolar assembly, to better understand how stress alters the spatial and chemical properties of the sub-nucleolar condensates, owing to their reorganization.

### The physicochemical properties of the condensates alter in response to DNA damage and repressed transcription, leading to the bipartite reorganization of the nucleolus

**Figure 6:**
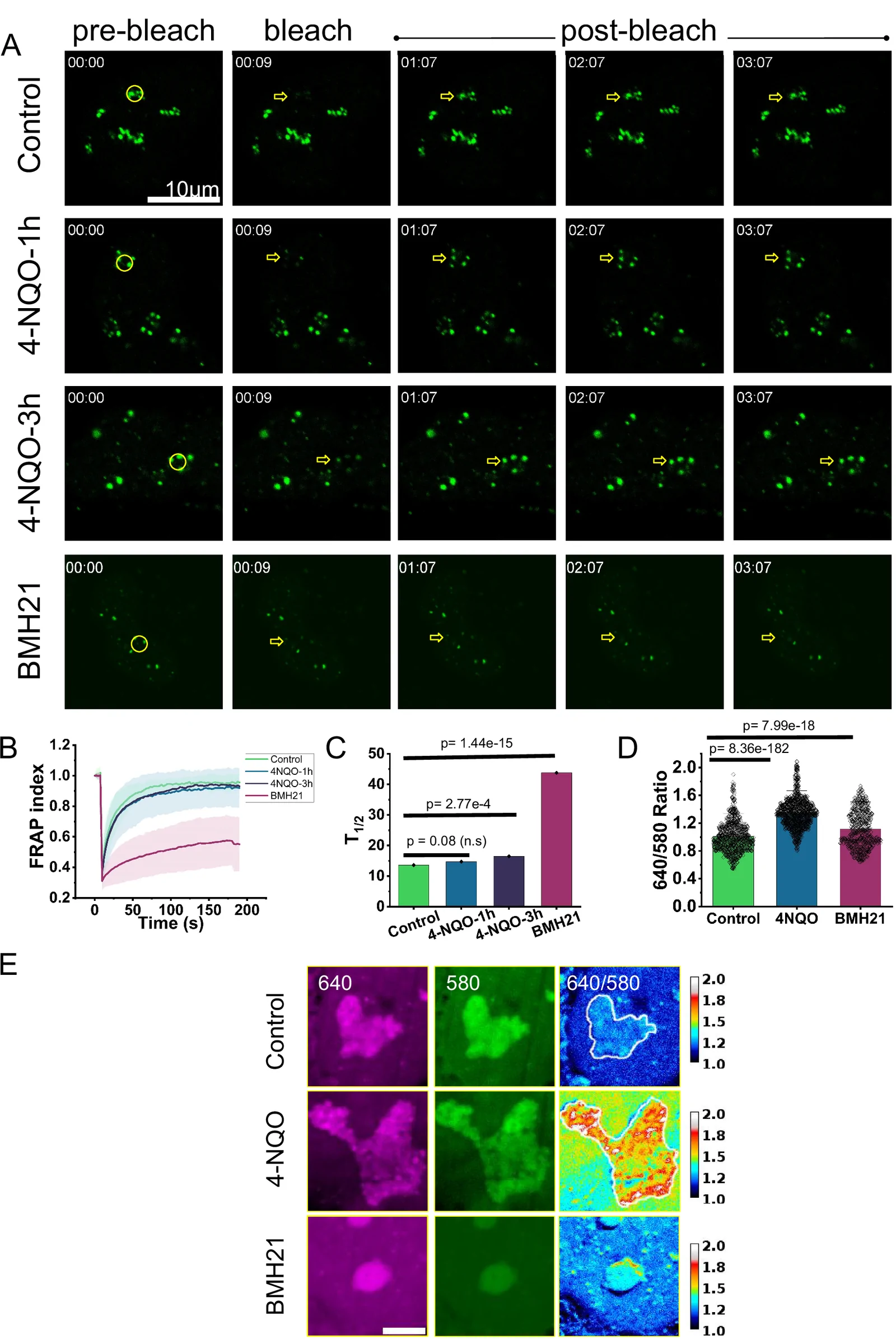
The biophysical properties of the condensates alter in response to repressed transcription, leading to the reorganized bipartite arrangement of the nucleolus. (**A**) Representative images of time-lapse of U2OS cells transiently transfected with GFP-UBF with a single nucleolus photobleached for every cell using a 488 laser, in control, and compared with cells treated with 1 µg/ml 4-NQO for 1 h and 3 h, or 2 µM BMH21 for 3 h, respectively, and (**B**) FRAP curve for the same experiment. Scale bar is 10 microns. Data represents mean ± SEM. *n* > 20 cells, from 3 independent experiments. (**C**) T-half (T_1/2_) in seconds is calculated from (**B**). (**D**) 640/580 ratios calculated using the experiment from (**E**) which shows U2OS cells incubated with 7.5 µm SNARF-4F for 30 min. The images show a single nucleolus from each treatment of 1 µg/ml 4-NQO for 1 h or 2 µM BMH21 for 3 h, respectively compared to the control cells. Scale bar is 5 microns. The samples were excited with 561 nm and emission was collected at 640 and 580 nm. Data represents mean ± SEM, *n* > 100 nucleoli, from 3 independent experiments. Statistical significance calculated using the K.S. test. Source data are available online for this figure.

Biomolecular condensates formed by RNA, proteins, or both, are seeded when increasing molecular concentration and binding affinity trigger phase separation (Berry et al, 2015; Brangwynne et al, 2011; Rai et al, 2023). These are altered in various stress conditions, and even though the various condensates still remain, changes in their composition can lead to perturbed dynamic properties of these biomolecular condensates. Therefore, we investigated the temporal dynamics of the FC-marker protein UBF1, which regulates active transcription within the nucleoli, using tools of fluorescent recovery after photobleaching (FRAP). Nucleoli in U2OS cells transiently expressing GFP-UBF (Dundr et al, 2002) were photobleached under normal conditions and upon both DNA damage (by 4-NQO) and transcriptional inhibition (by BMH21), using a 488 nm laser, and the recovery dynamics of the fluorescent fusion protein was captured for 3 min (Fig. 6A). Previous studies have shown that treatment with Actinomycin-D can cause reduction in the FRAP recovery of GFP-UBF as compared to other GC proteins like NCL, NPM1 and ribosomal protein S5, suggesting change in the FC protein dynamics which reorganizes into caps upon RNAPI-mediated transcription inhibition by Actinomycin-D (Chen and Huang, 2001). In our study, we observed a very modest effect on the GFP-UBF recovery upon DNA damage over 1 and 3 h (half time: T_1/2_ changed from 13.59 s ± 0.34 s in controls to 14.73 s ± 0.48 s (1 h) or 16.46 s ± 0.57 s (3 h) post 4-NQO incubation), while transcriptional inhibition (by BMH21) showed significant loss in recovery at the photobleached site (T_1/2_ = 43.77 ± 1.72 s) (Fig. 6B,C). The region of photobleaching was mainly restricted to one entire nucleolus per cell, wherein any recovery expected is mostly from the nucleoplasm. It is interesting to note that while both the DNA damage conditions and the RNAPI inhibition cause reorganization as evidenced by the flipping of the nucleolar compartments (Fig. 4A), there is still residual rRNA transcription under both the 4NQO damage conditions used (Fig. 5A,B,D); but once the reorganization is complete with total transcriptional inhibition by BMH21, the dynamics of UBF also changes in a pronounced manner (Fig. 6A–C). In-vitro studies have shown that loss in recovery of biomolecular droplets suggests a shift in the phases of these condensates (Lafontaine et al, 2021; Zhu, 2019; Sheu-Gruttadauria et al, 2026). Transcriptional inhibition is known to cause changes in the viscoelastic properties of the nucleolus (Riback et al, 2023). Therefore, we infer that the loss in recovery of GFP-UBF indicates transition of these condensates to a low-dynamics potential solid-like state compared to their original state when nucleolar transcription was active.

**Figure EV5:**
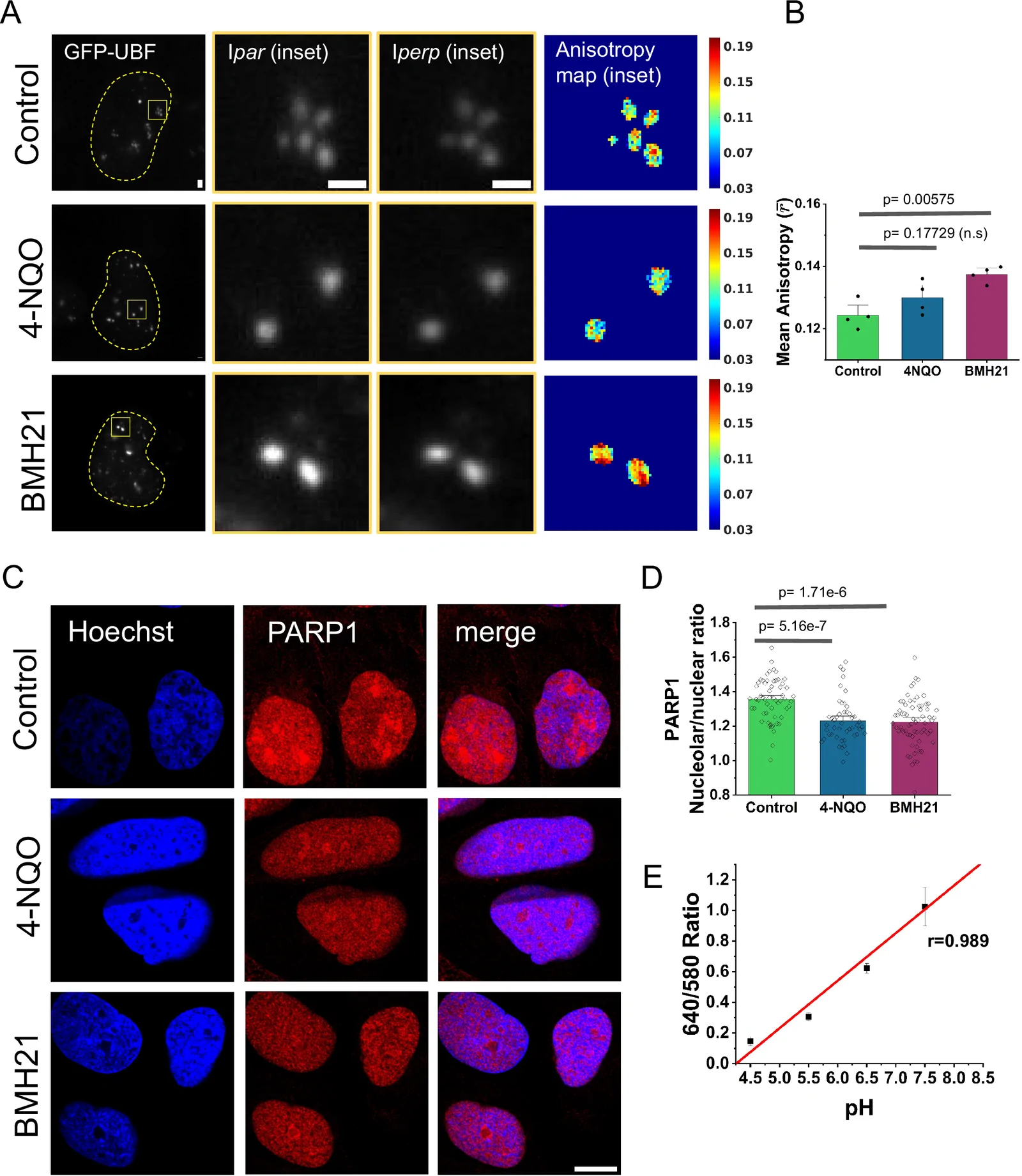
Rotational dynamics of GFP-UBF and stress-induced relocalization of PARP1 upon DNA damage and transcriptional inhibition. (**A**) U2OS cells were transfected with GFP-UBF. Representative nuclei from cells treated with 4-NQO (1 µg/ml, 1 h) and BMH21 (2 µM, 3 h) compared to control nuclei. Compared to control nuclei are shown in the leftmost column. The nuclear boundaries are drawn with a dotted line, and zoomed-in images of the nucleoli in the inset boxes are shown on the right. The parallel (Ipar) and perpendicular (Iperp) components of the emission light are collected and used to compute the anisotropy maps. Anisotropy maps of the representative nucleoli are shown in the rightmost columns. (**B**) Average of the mean anisotropy values of nucleolar GFP-UBF foci. Data represents mean ± SEM, *n* ≥ 200 (225; 217; 230) nucleoli over four independent experiments. Statistical significance calculated using a *t*-test. Scale bar is 10 µm. (**C**) U2OS cells stably transfected with a PARP1-chromobody-RFP (red) and stained with Hoechst (blue) allows the visualization of endogenous PARP1. Higher nucleolar intensity is seen in control cells, which is lost following 4-NQO and BMH21 treatment. (**D**)Ratio of mean nucleolar intensity to nucleoplasmic intensity of PARP1-chromobody-RFP are shown, *n* = 57, 47, 66 nuclei over three independent experiments. Data represents mean ± SEM, with statistical significance tested using the K.S test. Scale bar is 10 µm. (**E**) pH calibration curve for SNARF-4F. Data represents mean ± standard deviation (SD), *n* ≥ =277 nucleoli over three independent experiments. Source data are available online for this figure.

To further understand the biophysical properties of the FC condensates, we performed fluorescence anisotropy imaging (FAI) of GFP-UBF to probe the local order, viscosity and rotational constraints on the labeled proteins. FAI reports on rotational diffusion of fluorophore molecules and can provide insight into their local order within biological systems (Kesavan et al, 2020; Lakowicz, 2006; Saha et al, 2016). We measured the rotational diffusion of GFP-UBF fusion proteins transiently transfected in U2OS cells, by exciting the sample using polarized light. This light maximally excites GFP molecules with excitation dipoles aligned parallel to its polarization axis. The rotational movement of these proteins can be determined by the degree of depolarization in the emitted light. Therefore, if transition to an ordered solid-like state occurs, we could expect anisotropy values greater than those obtained from a random orientation of fluorophores. At the very least, an increase in anisotropy indicates reduced rotational mobility (Kesavan et al, 2020; Lakowicz, 2006). We observed that cells treated with BMH21 has significantly higher anisotropy values than control cells (Fig. EV5A,B), while cells treated with 4-NQO show only a modest and non-significant increase in anisotropy values compared to control cells. This result is in alignment with the FRAP data. BMH21, thus, leads to the disassembly of the RNAPI machinery, trapping UBF on the rDNA, which is more immobile. Additionally, a reduction in rRNA can cause a change in the molecular balance in the structures that form the signature sub-compartments (Caragine et al, 2019; Farley et al, 2015; Lafontaine et al, 2021; Lawrimore et al, 2021). This is because rRNA can serve as an ideal polyanionic scaffold for multivalent interaction among the glycine-arginine (GAR) rich proteins residing inside nucleolus. In the absence of the rRNA and hence negative charge, the IDRs of the GAR proteins can undergo homotypic self-association, thereby increase the crowding and potentially drive a significant phase transition of the nucleolar matrix (Feric et al, 2016; Riback et al, 2023).

The nucleolar proteins, along with their GAR-rich domains also often contain Intrinsically Disordered Regions (IDRs) enriched in charged and polar amino acids that promote multivalent, electrostatic, dipole–dipole and hydrogen bonding interactions with the newly synthesized rRNA (Dogra and Kriwacki, 2025). This facilitates an intrinsic pH gradient and helps maintain different chemical microenvironments simultaneously within the cell. The nucleolus thus inherently shows a lower pH than the rest of the nucleoplasm (Aryan et al, 2023; King et al, 2024; Martin et al, 2015). To further understand how the nucleolar environments can be affected with morphological remodeling of the tripartite condensate, we tested the effect of DNA damage and transcriptional downregulation on the pH of the cell nucleoli. To do this, we used the cell-permeant ratio-metric pH sensor Seminaphtharhodafluor 5-(and-6)-carboxylic acid, acetoxymethyl ester, acetate (SNARF-4F, AM ester), as done in previous studies (Aryan et al, 2023; King et al, 2024). This sensor when excited with 561 nm emits in both 580 and 640 nm range. The extent of protonation-deprotonation of the dye varies with cellular pH, which is reflected in the emissions collected at the two emission wavelengths (Marcotte and Brouwer, 2005). The ratio of the emissions at the two wavelengths is a proxy for the pH recorded inside the cells. Higher 640/580 ratios indicate higher pH and thus deacidification of the cell or organelle of interest. We observed that the 640/580 ratio increases significantly upon DNA damage and more modestly but significantly with RNAPI inhibition (Fig. 6D,E). Beyond just reducing rRNA levels, DNA damage can cause translocation of nucleolar proteins like NCL and NPM1 (Daniely et al, 2002b; Yang et al, 2016), and also PARP1, which synthesizes negatively charged poly-ADP-ribose (PAR) chains essential for rRNA processing (Aryan et al, 2023; Boamah et al, 2012). We have shown loss of nascent rRNA in our study—this can be a significant source of deacidification. But also as discussed, other sources of deacidification exist: one could be PAR (Poly-ADP-ribose) loss as PARP1 translocates from the nucleolus under conditions of stress (Rancourt and Satoh, 2009); yet another could be the translocation of DE tract proteins like NPM, NCL, and Ki-67 occupying the GC region and the nucleolar rim, which can contribute to deacidification.

To specifically see if these are indeed reasonable speculations, we performed experiments where we detected PARP1 with a chromobody that detects endogenous PARP1 in live cells (PARP1-cb-RFP). PARP1 is known to have nucleolar functions where it acts as a scaffolding platform for the various nucleolar proteins, aiding the ribosomal biogenesis (Boamah et al, 2012). We transfect U2OS cells with a plasmid containing the chromobody and then treat the cells with 4-NQO and BMH21 and fix it. We find that cells treated with 4-NQO and BMH21 show lower nucleolar PARP1 intensity as compared to the control (Fig. EV5C,D). This can certainly result in lower baseline nucleolar PARylation and thus contribute to deacidification.

Further, we also present data about the translocation of the DE tract proteins. We show that among the three, NPM shows a moderate decrease in nucleolar intensity, along with lower nucleolar to nucleoplasmic ratios, indicating that it shuttles out into the nucleoplasm upon direct inhibition of rRNA synthesis by BMH21 (Fig. EV3D,F,I). But NCL and Ki-67 shown significant loss of nucleolar pool (Fig. EV3G,H) and increased nucleoplasmic pool (Fig. EV3J,K) upon DNA damage by 4-NQO and inhibition of nascent rRNA synthesis by BMH21, adding to the evidence of local deacidification of the nucleolar core. Under homeostatic conditions, these proteins are in multi-protein complexes with nascent pre-rRNA, to maintain a low-pH microenvironment. Upon DNA damage or transcriptional inhibition, the loss of rRNA results in deprotonation of these proteins due to electrostatic repulsion, resulting in their outflux into the nucleoplasm. Together, loss of rRNA and PAR levels and translocation of the DE tract proteins help address the point about nucleolar deacidification more comprehensively beyond just speculation. These different sources of deacidification may also explain the differences in the degree of deacidification between 4NQO and BMH21.

Together, the data from immunofluorescence and smFISH (Figs. 4 and 5) and FRAP (Fig. 6) studies suggest that a decline in rRNA synthesis can alter the structural organization and material properties of the nucleolus. Further in the context of DNA damage, shuttling of nucleolar proteins, potential loss of nucleolar PAR chains, in addition to reduced rRNA levels, may alter the molecular profile of the nucleolus, potentially leading to a more pronounced nucleolar deacidification. The GFP-UBF FRAP (Fig. 6A) and pH studies (Fig. 6D,E), show that while there are common features, different stresses can alter the biophysical properties of sub-nucleolar condensates in more nuanced ways than just broad reorganization.

## Discussion

**Figure 7:**
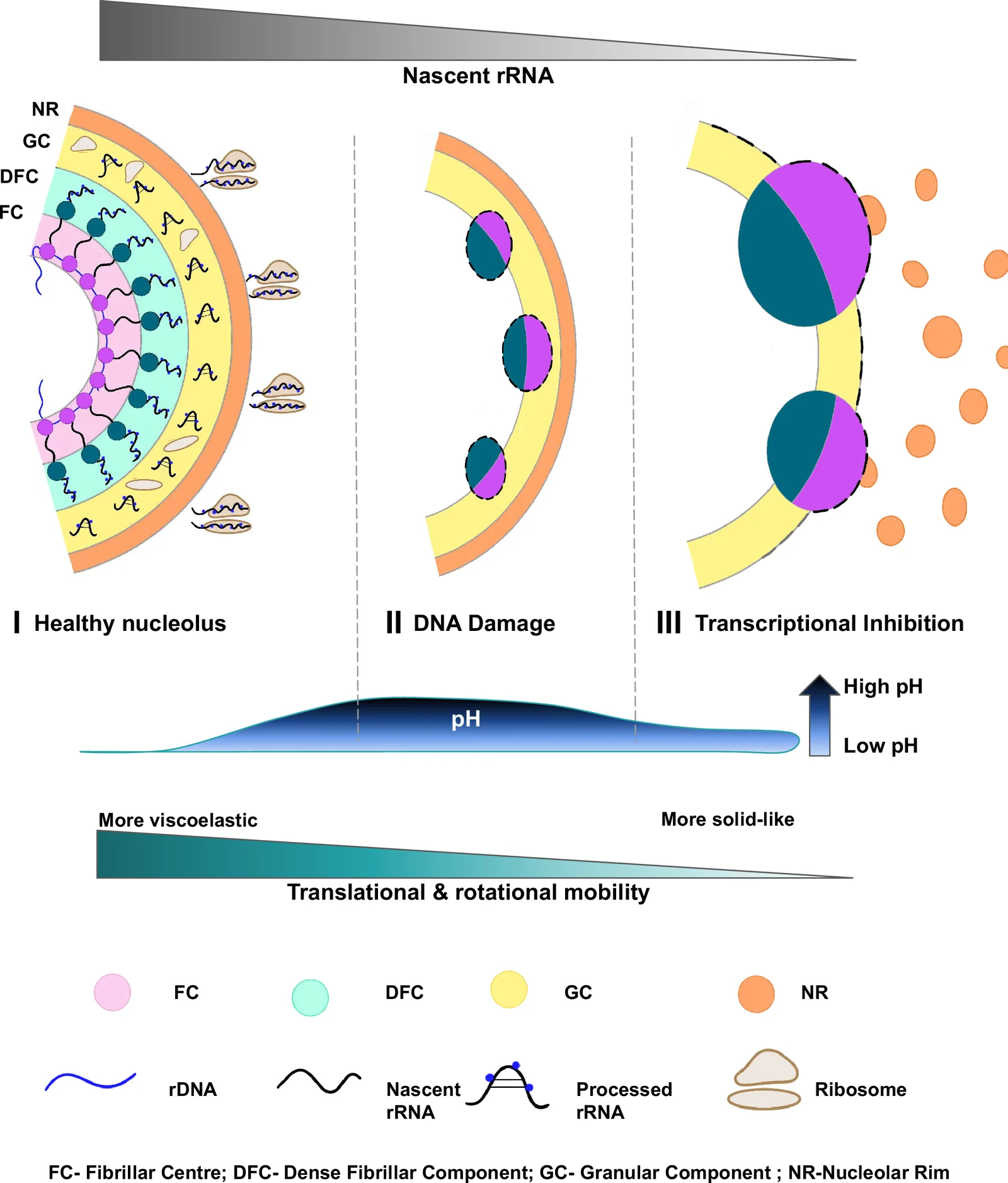
Schematic model for stress-induced nucleolar reorganization. (I) In a healthy homeostatic state, the nucleolus is characterized by a liquid-like tripartite architecture comprising multiple subdomains which aid in the transcription of rRNA for the formation of mature ribosomes. The FCs (marked in light pink, with magenta marking UBF) and DFCs (light green, with FIB labeled in dark green) within the nucleolus forms ring-like structure embedded within a pool of granular components. A fourth compartment called the nucleolar rim forms a ring around the nucleolar periphery. The rDNA (blue) is actively transcribed into nascent pre-rRNA (black) at the FC-DFC interface (which in itself looks like a nested ring within) and co-transcriptionally modified, forming mature ribosomes for directional export out into the nucleoplasm after assembly. This liquid-like state exhibits high molecular mobility and a balanced internal pH, maintained by rRNA molecules and charged disordered nucleolar proteins acting as essential proton carriers. A section of a single FC is shown in the cartoon for control cells. Upon global DNA damage, such as exposure to 4-NQO or CPT (II), the rRNA production is attenuated, initiating a nucleolar reorganization that leads to FC and DFC foci disrupting their ring-like organization. The GC pool of proteins, like NCL, which aid in DNA repair processes, shuttle outside into the nucleoplasm. This leads to a change in the pool of proteins within the nucleoli with a missing scaffold of rRNA, shifting the interaction paradigm between the proteins and rRNA which now no longer are available to serve as proton carriers, leading to an increase in nucleolar pH. Loss of DE tract proteins and nucleolar PARP1 can further contribute to deacidification upon DNA damage. (III) Total abrogation of rRNA levels due to inhibition of RNAPI-mediated transcription leads to flipping out of the tripartite organization of the FC-DFC-GC into a bipartite structure. In this final stage of pronounced reduction in nascent rRNA, the nucleolus undergoes a phase transition to a potential solid-like state with diminished protein mobility. This is marked by the structural inversion, where the FCs completely move to the nucleolar periphery facing outwards, whereas the FIB is at the periphery facing the nucleolar interior. Ultimately, the model illustrates that a graded reduction in nascent rRNA levels dictates the transition of the nucleolus from a dynamic biosynthetic hub to a rigid, segregated body with fundamentally altered physical and biochemical properties.

The nucleolus is perhaps the best-known phase-separated sub-organelle of the cell. Despite that, its dynamic organization inside the cell, especially under conditions of environmental stress is underappreciated still, and recent studies have opened new avenues for studying this organelle with improved spatial resolution (Maiser et al, 2020; Yao et al, 2019). In this study, we investigated nucleolar organization at a higher spatial resolution to understand its structure-function correlation better. We first used the tools of expansion microscopy to study the nucleolar sub-compartments (Fig. 1), with specific resident proteins for the sub-compartments as a proxy to label them. We found that the innermost compartment protein, UBF1, itself shows a shell-like appearance with a pool of multiple such rRNA-making factories occupying the entire nucleolus. Shells of the DFC surrounded these and are embedded in the GC (Fig. 1E). Nucleolar reorganization with DNA damage or transcriptional stress (Fig. 2) could be followed beyond just simple cap formation. Shape and size parameters of nucleoli could be related to rRNA production activity. We further wanted to understand how nucleolar RNA is spatially arranged within the cell nucleolus. Probing the cells with both mature and nascent pre-rRNA (Fig. 3) we find that rRNA is synthesized in the FC-DFC boundary and then gradually progresses to the GC potentially undergoing modifications and cleavage in line with previous studies (Yao et al, 2019). The mature rRNA then gets assembled at GC as seen by the homogenous staining of 28S rRNA probe across the entire GC with a small zone of low intensity, which marks the core of the FC marked with UBF (Fig. 3B). This corroborates that mature rRNA is mainly excluded from the FC. The nascent pre-rRNA, on the other hand, forms an intermediate shell that partially overlaps with the DFC region marked by FIB (Fig. 3F). The cells, upon stress undergoes reorganization of the nucleolus that leads to a switch from the tripartite to the bipartite arrangement wherein the inner compartment flips outside the nucleolar periphery, and the DFC forms a clustered condensate inwards. We observe this upon treatment with DNA-damaging 4-NQO and RNAPI transcription-inhibiting BMH21 (Figs. 2 and 4A). Although the 28S rRNA levels showed divergent trends upon the aforementioned dosage of DNA damage, and RNAPI inhibition, its correlation with the nascent rRNA levels reduced dramatically with decreasing synthesis of pre-rRNA (Fig. 4A). The nucleolar proteins show a bipartite arrangement as seen by earlier studies(Al-Baker et al, 2004; Boulon et al, 2010). But what was interesting to note is that upon DNA damage with 4NQO, rRNA levels do not get abrogated entirely, and nascent rRNA levels are a predictor of nucleolar reorganization (Figs. 4 and 5). Other DNA-damaging agents may cause different nucleolar reorganization phenotypes, correlated with rRNA level reductions (Fig. EV4D,E). In our experiments, the reduction in rRNA synthesis leads to coalescence of the remaining FC-DFC modules, the size of the FC-DFC units increases, while the nucleolar number remains stable. This is most likely because, although the NOR-associated “factory sites” prevail, their functional capacity to produce mature ribosomes is diminished due to stress. The prevailing nascent rRNA transcripts then decorate these coalesced foci, initiating the cap formation, which is seen as rings of 5’ETS rRNA upon treatment with 4-NQO (Figs. 4A and 5A). We designed a metric to study the nucleolar reorganization at the level of individual nucleoli across cells, based on the distance of the UBF1 foci from the nucleolar periphery. We simultaneously measure the nucleolar nascent rRNA levels in the same nucleoli. Nucleolar reorganization emerges to be strongly correlated to rRNA levels (Fig. 5B). Persisting damage for longer periods or transcriptional inhibition shows further reduction in nascent rRNA synthesis, which progressively remodels the nucleoli, to reorganize its proteins and compartments, gradually disrupting the RNA–protein interactions within the nucleoli which was essential for the integrity of the tripartite structure. Eventually the compartments form the nucleolar caps observed by a shift from positive to negative scoring of the distance metric between UBF1 foci and the nucleolar periphery (Fig. 5B). Several GC-resident proteins like NCL and NPM shuttle out to nucleoplasm to aid in the DNA repair process by p53-dependent pathway (Al-Baker et al, 2004; Daniely et al, 2002b; Yang et al, 2016). There is a concomitant reduction in rRNA synthesis, which in turn leads to increased protein–protein interaction due to higher affinity and missing rRNA, which generally scaffolds multivalent interaction among different types of proteins. We thus observed a liquid-to-low mobility, potential solid-like transition in one of the sub-nucleolar condensates as evidenced by reduced GFP-UBF dynamics using FRAP and FAI (Figs. 6A and EV5A,B). A limitation is that in the FRAP and FAI experiments intensities from exogenously-expressed GFP-tagged UBF was measured and the endogenous pools of UBF, but relative changes with stress could still be inferred. To minimize this ambiguity, we have used cells with low to medium levels of expression and confirmed that the mobility trends are consistent across the range of expression levels analyzed. Nonetheless, this caveat of exogenous expression and competition with unlabeled endogenous protein should be taken into consideration while interpreting the results, and *GFP* knock-ins at endogenous *UBF* gene loci may serve towards improved mobility assays in the future. The GFP-UBF foci show reduced FRAP recovery upon total loss of rRNA with direct RNAPI inhibition by BMH21 (Fig. 6B,C). The 4NQO doses still showed residual rRNA and hence far less pronounced reductions in GFP-UBF dynamics. The FAI also showed a corresponding increase in anisotropy upon 4NQO and BMH21 treatments, suggesting reduced rotational mobility upon DNA damage and transcriptional inhibition. Concomitantly, the pH gradient within the nucleolus thus shifts towards higher pH values due to reduced negative charge from synthesizing rRNA (Fig. 6D,E). Interestingly, 4NQO shows a more pronounced effect here than BMH21. This may not be unreasonable, as there are determinants of the pH gradient beyond just rRNA. The alteration in pH is due to a change in molecular association underlying the phase separation of nucleoli due to differences in local protein concentration, ionic strength, and hydrogen bonding (Detres et al, 2025; Dogra and Kriwacki, 2025). A recent study has shown that one of the chemical signaling components responsible for the deacidification of the nucleolus upon stress or transcriptional decline, is poly-ADP-ribose-polymerase1 (PARP1) (Aryan et al, 2023). It is seen that in response to stress nucleolar PARP1 pool delocalizes to the nucleoplasm (Fig. EV5C,D). The enzymatic activity of PARP1 causes poly-ADP-ribosylation (PARylation) of multiple nucleolar proteins, which help maintain the nucleolar scaffold during the rRNA synthesis. This is perturbed during stress, leading to a change in the proton gradient and altering the pH of the nucleoli (Aryan et al, 2023). PAR chains are negatively charged and loss of nucleolar pools and nucleoplasmic recruitment of PARP1 with DNA damage, can cause more pronounced deacidification with loss of both nascent rRNA and PAR. DNA damage also causes the translocation of nucleolar proteins rich in DE tracts (Feric et al, 2016; Yang et al, 2016) (Fig. EV3D–K). Together nascent rRNA reduction and alteration of the biophysical properties of the nucleolus, marks initiation of formation of nucleolar caps, as the rRNA now gradually forms a hemi ring due to reduced available networks (Fig. 4A). Finally, the cap formation is complete when persistent damage or RNAPI inhibition leads to significant reduction in nascent transcripts and the nucleolar proteins forming the FC-DFC (UBF1, and FIB in this study). This eventually leads to complete transformation of the viscoelastic sub-organelle, to a low-dynamics multiphase condensate with increased solid-like properties in the FCs (Fig. 5A–C). Altogether, this study highlights how the ongoing nascent rRNA synthesis regulates the physical and chemical properties of the multiphase nucleolus and its reorganization upon stress (Fig. 6), which can eventually lead to repair of damaged DNA lesions within the nucleoli, if any. So instead of active DNA damage signaling for rDNA repair, nucleolar reorganization can arise naturally from the biophysical changes to the nucleolus under stress (Fig. 7). Given that nucleolus plays a critical role in health and diseases, further investigation of how individual segments of rRNA, nascent or mature, and other noncoding RNA (modulating rRNA modification processes and ribosome assembly), or nucleolar PARylation and pH gradients, can affect the physicochemical properties of the sub-organelle will be important for fundamental understanding of nucleolar function.

## Methods

**Table:** Reagents and tools table

Reagent/resource	Reference or source	Identifier or catalog number		
**Experimental models**				
**U2OS**	**ATCC**	**HTB-96, lot no. 70025046**		
**Recombinant DNA**				
**GFP-UBF**	**Addgene**	**Cat #17656**		
**PARP1 Chromobody (TagRFP)**	**Chromotek**	**Cat # xcr**		
**Antibodies**				
**Rabbit UBF1 (1:1000 / 1:500)**	**Abcam**	**ab24487**		
**Rabbit UBF1 (1:1000/ 1:500)**	**Invitrogen**	**PA5-82245**		
**Rabbit NPM1 (1:1000/1:500)**	**Abcam**	**ab183340/15440**		
**Rabbit POLR1A (1:1000/1:500)**	**Abcam**	**ab222065**		
**Mouse NCL (1:1000/1:500)**	**CST**	**E5M7K**		
**Chicken FIB (1:500)**	**Invitrogen**	**PA5-143565**		
**Rabbit Ki-67 (1:500)**	**Abcam**	**ab16667**		
**Mouse H2AX(Ser139) (1:1000)**	**Merck Millipore**	**JWB301**		
**Rabbit secondary 488 (1:1000/ 1:500)**	**Thermo**	**A11034**		
**Rabbit secondary 568 (1:1000/ 1:500)**	**Thermo**	**A11011**		
**Rabbit secondary 647 (1:1000/ 1:500)**	**Thermo**	**A21245**		
**Rabbit secondary atto 647 (1:500)**	**CST**	**40839**		
**Mouse secondary 488 (1:1000/ 1:500)**	**Thermo**	**A11029**		
**Mouse secondary atto 647 (1:500)**	**CST**	**50185**		
**Chicken secondary 488 (1:1000/ 1:500)**	**Thermo**	**A11039**		
**Chicken secondary 647 (1:1000)**	**Thermo**	**A21449**		
**Oligonucleotides and other sequence-based reagents**				
**5’ ETS -atto 488**	**Pixel Biotech** 5’ETS rRNA (44 20 -nucleotide DNA oligos)	**Probe #**	**Probe (5'-> 3')**	
		5ETS_1	ccagaggacagcgtgtcagc	
		5ETS_2	tcggacgcgcgagagaacag	
		5ETS_3	gagacgagaacgcctgacac	
		5ETS_4	gggaaggggcggcggacaac	
		5ETS_5	gcggagcgagaaggacggtc	
		5ETS_6	tgtctgccgcgtcagaggac	
		5ETS_7	caagtcgacaaccactggag	
		5ETS_8	cgagagggcagcacgacggg	
		5ETS_9	gagccgacgctcgcgcaaac	
		5ETS_10	tcgggcacctgagggacaac	
		5ETS_11	gaacgacacaccaccgttcg	
		5ETS_12	tcgaaccccacaccgacgag	
		5ETS_13	tcgtctcgtctcactcaaac	
		5ETS_14	agaggggagcacgggacgtg	
		5ETS_15	ggtcggaggcagaacggcag	
		5ETS_16	cgaagtcaacccacacacga	
		5ETS_17	ctcgagcgttcgcgttcagg	
		5ETS_18	ctgcggtacgaggaaacacc	
		5ETS_19	cgagggacgcctggggaagg	
		5ETS_20	tagagaaggcttttctcacc	
		5ETS_21	caaggcacgcctctcagatc	
		5ETS_22	aggcagagacagaggcggcg	
		5ETS_23	tacggcagcgctaccataac	
		5ETS_24	tcgagggggaagcggaggag	
		5ETS_25	cagtaggcgacgagccgcac	
		5ETS_26	cgactcggaagggggaggcg	
		5ETS_27	gtcacgactcggcacgggag	
		5ETS_28	cacgcaaacgcggtcgtcgg	
		5ETS_29	gctcgtgattctcgtccatc	
		5ETS_30	gagcgaccgcagccacgaac	
		5ETS_31	cacagacacgagggcggtcc	
		5ETS_32	cgggccaccaggaaaacacg	
		5ETS_33	cggggagaaacctcaggcac	
		5ETS_34	cggggaagagggcacagacg	
		5ETS_35	tcggggggaagaagaggatc	
		5ETS_36	aacggacgtgaagccggtga	
		5ETS_37	acggggaaggcgcgagaaag	
		5ETS_38	gagaagacgagagaccacgg	
		5ETS_39	cgacccggttcggaagagcg	
		5ETS_40	acacgtcgcacgaacgcctg	
		5ETS_41	caaggcggaggtcgacccac	
		5ETS_42	cacacgacggccaagggagg	
		5ETS_43	aagcgaggaggacgacggac	
		5ETS_44	gtaaggtagagcgcggcgag	
**18S rRNA -atto 488**	**Pixel Biotech** 18S rRNA (48 20-nucleotide DNA oligos)	**Probe #**	**Probe (5'-> 3')**	
		18S_1	tatgctactggcaggatcaa	
		18S_2	acttagacatgcatggctta	
		18S_3	cattcgcagtttcactgtac	
		18S_4	gagcgaccaaaggaaccata	
		18S_5	accacagttatccaagtagg	
		18S_6	tcggcatgtattagctctag	
		18S_7	gggttggttttgatctgata	
		18S_8	tatctagagtcaccaaagcc	
		18S_9	gatagggcagacgttcgaat	
		18S_10	acggcgactaccatcgaaag	
		18S_11	ggtcgggagtgggtaatttg	
		18S_12	tatttttcgtcactacctcc	
		18S_13	cctcgaaagagtcctgtatt	
		18S_14	gtggactcattccaattaca	
		18S_15	tccaatggatcctcgttaaa	
		18S_16	tacgctattggagctggaat	
		18S_17	acgagctttttaactgcagc	
		18S_18	acactcagctaagagcatcg	
		18S_19	tcaaagtaaacgcttcgggc	
		18S_20	gcctgctttgaacactctaa	
		18S_21	attattcctagctgcggtat	
		18S_22	acaaaatagaaccgcggtcc	
		18S_23	cttaatcatggcctcagttc	
		18S_24	gtccaagaatttcacctcta	
		18S_25	ttcttggcaaatgctttcgc	
		18S_26	ccgactttcgttcttgatta	
		18S_27	ggtatctgatcgtcttcgaa	
		18S_28	catcgtttatggtcggaact	
		18S_29	ggaacccaaagactttggtt	
		18S_30	tcagctttgcaaccatactc	
		18S_31	cttccgtcaattcctttaag	
		18S_32	tcccgtgttgagtcaaatta	
		18S_33	atcaatctgtcaatcctgtc	
		18S_34	cacccacggaatcgagaaag	
		18S_35	aactaagaacggccatgcac	
		18S_36	taaccagacaaatcgctcca	
		18S_37	cagagtctcgttcgttatcg	
		18S_38	gtcgcgtaactagttagcat	
		18S_39	cttgtccctctaagaagttg	
		18S_40	tgttattgctcaatctcggg	
		18S_41	acatctaagggcatcacaga	
		18S_42	aatggggttcaacgggttac	
		18S_43	cctcgttcatggggaataat	
		18S_44	atcaacgcaagcttatgacc	
		18S_45	tgtgtacaaagggcagggac	
		18S_46	cactaaaccatccaatcggt	
		18S_47	agatagtcaagttcgaccgt	
		18S_48	cacctacggaaaccttgtta	
**28S rRNA -atto 565**	**Pixel Biotech** 28S rRNA (48 20 -nucleotide DNA oligos)	**Probe #**	**Probe (5'-> 3')**	
		28S_1	atgcttaaattcagcgggtc	
		28S_2	aatcctggttagtttcttct	
		28S_3	ctcgatcagaaggacttggg	
		28S_4	cggtatttagccttagatgg	
		28S_5	acttgttgactatcggtctc	
		28S_6	cttttcaactttcccttacg	
		28S_7	tacctcttaacggtttcacg	
		28S_8	agggaaccagctactagatg	
		28S_9	agctatcctgagggaaactt	
		28S_10	aatcattcgctttaccggat	
		28S_11	ggcttcttacccatttaaag	
		28S_12	ttctgcttaccaaaagtggc	
		28S_13	gtctatatcaaccaacacct	
		28S_14	gagttgttacacactcctta	
		28S_15	cattttcagggctagttgat	
		28S_16	tttgctactaccaccaagat	
		28S_17	ccttcaaagttctcgtttga	
		28S_18	ttcaactgctgttcacatgg	
		28S_19	cctgcccttcacaaagaaaa	
		28S_20	cttacctacattgttccaac	
		28S_21	ttatcccgaagttacggatc	
		28S_22	agagagagagagagagggcg	
		28S_23	cttcgcgatgctttgtttta	
		28S_24	agagcactgggcagaaatca	
		28S_25	cgcgcttcattgaatttctt	
		28S_26	gcatttggctaccttaagag	
		28S_27	attcatgcgcgtcactaatt	
		28S_28	acagtgggaatctcgttcat	
		28S_29	tttcgctggatagtaggtag	
		28S_30	tattctacacctctcatgtc	
		28S_31	agagtagtggtatttcaccg	
		28S_32	ctcaccgggtcagtgaaaaa	
		28S_33	ttaccgtttgacaggtgtac	
		28S_34	tttcacggtctgtattcgta	
		28S_35	aggtcagaaggatcgtgagg	
		28S_36	gtggtaacttttctgacacc	
		28S_37	aagcgacgtcgctatgaacg	
		28S_38	aggaagagccgacatcgaag	
		28S_39	cttggtgaattctgcttcac	
		28S_40	ccctattagtgggtgaacaa	
		28S_41	ctgtctcacgacggtctaaa	
		28S_42	ctcgtactgagcaggattac	
		28S_43	aaatgtctgaacctgcggtt	
		28S_44	tcataatcccacagatggta	
		28S_45	attctgacttagaggcgttc	
		28S_46	ggtcgtctacgaatggttta	
		28S_47	tgctctgctacgtacgaaac	
		28S_48	tcgagggctgactttcaata	
**Chemicals, enzymes, and other reagents**				
**McCoy 5A media**	**Sigma**	**M4892**		
**FBS**	**Gibco**	**16000-044**		
**PenStrep-glutamine**	**Gibco**	**10378-016**		
**FluoroBrite media**	**Gibco**	**A1896701**		
**X-tremeGENE™ HP DNA**	**Roche**	**6.4E+09**		
**BMH21**	**Abcam**	**ab219384**		
**5-Ethynyl uridine (5-EU)**	**Jena Biosciences**	**CLK-N002-10**		
**Tris pH ~8**	**Sigma**	**T6066**		
**Camptothecin**	**Sigma**	**C9911**		
**THPTA (tris-hydroxypropyltriazolylmethylamine)**	**Sigma**	**762342**		
**CuSO4**	**Sigma**	**C1297**		
**Tamra azide**	**Lumiprobe**	**A7130**		
**Sodium ascorbate**	**Sigma**	**11140**		
**PBS-EDTA (ethylenediaminetetraacetic acid)**	**Sigma**	**E6758**		
**Paraformaldehyde (PFA)**	**Sigma**	**P6148**		
**PBS (Phosphate-buffered saline)**	**Sigma**	**P5493**		
**Triton X-100**	**Sigma**	**T8787**		
**Bovine serum albumin (BSA)**	**Himedia**	**TC545**		
**Glutaraldehyde**	**Sigma**	**G6257**		
**Acryloyl-X SE (AcX-SE)**	**Invitrogen**	**A20770**		
**APS**	**Sigma**	**A3678**		
**TEMED**	**Sigma**	**T7024**		
**Formamide**	**Ambion**	**AM9344**		
**Hybridization buffer**	**Stellaris**	**SMF-HB1-10**		
**NGS (normal goat serum)**	**Thermo-fisher**	**5425S**		
**Nuclease-free water**	**Ambion**	**AM9932**		
**SSC**	**Ambion**	**AM9770**		
**10X PBS (For RNA work)**	**Ambion**	**AM9625**		
**Antifade mounting medium**	**Vectashield**	**H-1000**		
**SNARF-4F**	**Invitrogen**	**S23921**		
**pH calibration kit**	**Thermo-fisher**	**P35379**		
**Fluorescein sodium salt**	**Sigma**	**46960**		
**Sodium Acrylate**	**Sigma**	**408220**		
**Acrylamide**	**Sigma**	**A4058**		
*N*,*N***-Methylenebisacrylamide**	**Sigma**	**M1533**		
**Sodium chloride**	**Sigma**	**746398**		
**PBS**	**Sigma**	**P5493**		
**Triton X-100**	**Sigma**	**T8787**		
**EDTA**	**Sigma**	**E6758**		
**Tris-Cl**	**Sigma**	**T6066**		
**Sodium chloride**	**Sigma**	**746398**		
**Proteinase-K**	**Sigma**	**P2308**		
**Details of expansion microscopy**		**Monomer Solution**	**Stock Solution**	**Final concentration (g/100ml)**
		Sodium Acrylate	38g/100ml	8.6
		Acrylamide	40g/100ml	2.5
		N,N-Methylenebisacrylamide	2g/100ml	0.15
		Sodium chloride	5M	11.7
		PBS	10X	1X
		Water		
		**Digestion buffer**	**Stock solution**	**Final concentration (g/100ml)**
		Triton X-100	-	0.50g
		EDTA	0.5M, pH-8	0.2ml
		Tris-Cl	1M, pH-8	5ml
		Sodium chloride	-	4.67g
		Water	-	-
		Proteinase-K	800U/ml	8U/ml
**Software**				
**MATLAB**	**MathWorks Inc**. https://www.mathworks.com			
**FIJI**	https://doi.org/10.1038/nmeth.2019			
**IMARIS**	**licensed B7y4-du9y-5k5j-76bn**			
**ORIGIN (Pro)**	**OriginLab Corporation, Northampton, MA, USA**			
**Imaging Hardware**				
**Nikon Eclipse Ti2 E with Yokogawa CSU W1 So-Ra**	**Nikon**			
**Olympus FV3000 laser scanning confocal microscope**	**Olympus**			
**Abberior STEDYCON**	**Abberior**			
**Optosplit II**	**Cairn Research, Kent, England**			

### Cell culture and treatments

U2OS cells were grown in McCoy 5A media (Sigma, M4892) supplemented with 10% FBS (Gibco, 16000-044) and 1% PenStrep-glutamine (Gibco, 10378-016). Cells were grown in a T-25 flasks (Tarsons, 950040) and incubated in a CO_2_ incubator (Eppendorf Galaxy, 170S) set to 37 °C and 5% CO_2_. For experiments and imaging, the cells were plated in a glass-bottom dish (Genetix, 200350) at least 24 h before the experiments. For live-cell experiments, the cells were imaged directly in a supplemented FluoroBrite medium (Gibco, A1896701). The plates were placed in the live-cell chamber and maintained at 37 °C. The cells are equilibrated for 15 min prior to imaging. Transfection was performed using the X-tremeGENE™ HP DNA Transfection Reagent (Roche, 6366236001) following the manufacturer’s protocol. For inhibition of RNA Polymerase-I, BMH21 (ab219384) was used at 2 uM for 3 h. For global DNA damage to the cells 4-nitroquinoline-*N*-oxide (4-NQO) was used at 1 µg/ml for 1 or 3 h. For labeling the RNA, the cells were pulse labeled with 5-Ethynyl uridine (5-EU, CLK-N002-10, Jena Biosciences) for 1 mM for 30 min and then click chemistry was performed to visualize them. The protocol was adapted from previously described protocols (Jao and Salic, 2008; Loschberger et al, 2014). Briefly, after fixation and permeabilization, the cells were washed with Tris-buffered saline (TBS) buffer. The cells were then incubated with click reaction cocktail (CRC) for 30 min. A 500 µl solution of CRC was prepared as follows: 400 µl of 100 mM Tris (pH ~8, T6066, Sigma), 10 µl of 100 mM CuSO_4_ (C1297, Sigma), 39 µl of 50 mM THPTA (tris-hydroxypropyltriazolylmethylamine) (762342, Sigma), 1 µl of 6.2 mM azide dye (Tamra azide, A7130, Lumiprobe), 50 µl of 1 M sodium ascorbate (11140, Sigma). The cells are then washed with PBS-EDTA (ethylenediaminetetraacetic acid, E6758, Sigma 10 mM with 1X PBS) for 10 min and followed by two washes with 1X PBS, and the immunofluorescence assay can be continued as mentioned below.

### Immunofluorescence (IF)

The cells were fixed in 4% paraformaldehyde (PFA, Sigma P6148) in 1X PBS (phosphate-buffered saline, Sigma P5493) for 20 minutes at room temperature. The cells were washed twice with 1X PBS, followed by permeabilization with 0.3% Triton X -100 (Sigma, T8787) in 1X PBS for 10 min at room temperature. The cells were washed twice with 1X PBS and blocked with 5% Bovine serum albumin (BSA, Himedia TC545) for an hour. The cells were incubated with the primary antibodies in 5% BSA, at 4 °C overnight. After two washes in 1X PBS, the cells were incubated with the secondary antibodies in 5% BSA, for 2 h, at room temperature, in the dark. The cells were washed thrice in 1X PBS and treated with Hoechst (1 µg/ml) in 1X PBS for 10 min.

### Gelation, digestion, and expansion

For expansion microscopy, we adapted from previously described protocols (Asano et al, 2018; Cho et al, 2018; Gao et al, 2017; Tillberg, 2016; Wassie et al, 2019). Cells were grown on coverslips placed in plastic bottom dishes; to fix and stain as mentioned above, the cells were re-fixed with 0.25% glutaraldehyde (G6257, Sigma) for 10 min. The biomolecules were then anchored overnight using 0.1 mg/ml Acryloyl-X SE (AcX-SE, A20770, Invitrogen) in PBS overnight at room temperature. The cells were washed twice with PBS for 15 minutes each. Meanwhile, the gelling components are thawed in ice namely, monomer solution (constituents in Reagents and Tools Table), APS (A3678, Sigma) and TEMED (T7024, Sigma) stock solutions. The gelling solution was then made using monomer solution, water, 10% TEMED solution, 10% APS in 47:1:1:1 ratio, in this order and vortexed for a few seconds. Immediately after, PBS was removed, the glass coverslips were removed from the plastic bottom dish using forceps. About 200 μl of gelling solution was added to a parafilm kept in a humidified chamber, and the coverslip was inverted (with the cells facing downwards) on the gelling solution. The chamber was then kept for polymerization for an hour at 37 °C. The gel is trimmed using a razor blade. The gel attached to the coverslip is then placed in a six-well plate with a digestion buffer (2–3 ml, constituents in Reagents and Tools Table) prepared beforehand, with freshly added proteinase-K (8 U/ml). The gel is left immersed in the digestion buffer overnight (12–16 h) at room temperature (28–30 °C) in the dark. Once the gel got detached from the coverslip post digestion, it was transferred to a petri dish using a paintbrush or another coverslip. The petri dish is filled with water to expand the gel. The water is exchanged thrice every 20 min. Excess water was then removed and the gel was cut into smaller pieces and placed on a poly-D- lysine-coated coverslip for imaging.

### rRNA FISH

For RNA FISH, we adapted from previously described protocols (Dhuppar and Mazumder, 2018, 2020; Mazumder et al, 2013; Pasnuri et al, 2023). Briefly, cells were fixed with 4% PFA in nuclease-free (NF) PBS for 20 min at room temperature, followed by permeabilization with 0.3% Triton X-100 in NF PBS for 10 min at room temperature. The cells were then washed twice with PBS, followed by blocking (if combined with IF) using 1% NF BSA and 2% NGS (normal goat serum) for an hour. The cells were then rinsed once each with 1X PBS and wash buffer. The cells were then incubated with a hybridization mix consisting of hybridization buffer (Stellaris), 10% formamide (AM9344, Ambion), RNA FISH probes and primary antibody (optional, if combined with IF), overnight at 37°C in a dark, humidified chamber. The next day, the cells were washed with wash buffer twice for 30 min each at 37°C, in a humidified chamber. The cells were then briefly washed with 2X SSC and then incubated in secondary antibodies in 2% NGS in 1X PBS at room temperature, in the dark. The cells were then rinsed in 1X PBS, and stained with 1 µg/ml Hoechst dye for 10 min. Given the high density of rRNA within nucleoli, individual rRNA molecules are not distinguished like mRNA in the previous studies. But the distinct spatial localization of 5’ETS and 28S are clear. Intensities of each species are measured at a single nucleolar level for quantification.

### pH measurements using SNARF-4F

SNARF-4F (S23921, Invitrogen) stock were made in DMSO, at 5 mM. About 7.5 µM was used in live, confluent U2OS cells seeded on a culture dish, and incubated for 30 min. In case of treatments using 4-NQO and BMH21, SNARF-4F was added in the last 30 min of their respective incubation times. SNARF-4F was aspirated, and the cells were imaged in Fluorobrite along with the treatment or vehicular control (DMSO). The control plates were imaged first, and then the pH calibration was done using pH calibration kit (P35379, Thermo Fisher) according to the manufacturer's protocol. Similar results were obtained using a different pH sensor dye called pHrodo-AM where cells were incubated in pHrodo -AM (1:1000) for 30 min and imaged using 488 nm laser (Appendix Fig. S3).

### Fluorescence anisotropy

For anisotropy experiments protocols were adapted from previous work (Kesavan et al, 2020). Briefly, U2OS cells were seeded on confocal dishes, and then transiently transfected with GFP-UBF after 24 h. Fresh media is replenished in the dishes 7 h post transfection, and imaged approximately, after 42 h from transfection. The cells were either treated with BMH21 or 4-NQO or DMSO (as control) in imaging media (Fluorobrite, Invitrogen). Before imaging, the cells are equilibrated for 15 min and then imaged with the inhibitors in the media.

The parallel and perpendicular channels were segmented out and aligned using a customized code on MATLAB. Using the most focussed plane, individual nucleoli were cropped out with a 60 × 60 square ROI, the parallel and perpendicular channels were registered. The UBF foci were segmented after contrast adjustment, using intensity-based Otsu thresholding and a constant value added to the code-generated threshold value, and an applied area filter, using the parallel channel. It is to be noted that the contrast adjustments were used only for segmentation while the raw intensity values were used for anisotropy calculation. For each nucleolus, the average I_par_ and I_perp_ for all UBF1 foci were computed. The Anisotropy, *r*, was calculated using the formula $r=\frac{\left(Ipar-{G\; I}{{perp}}\right)}{\left(I{par}+2\,\times G\times I{perp}\right)}$, where *I*_par_ is the intensity of the parallel channel and *I*_perp_ the intensity of the perpendicular channels. The anisotropy map is generated only for the UBF foci by annotating the rest of the pixel value as zero. *G* is the correction factor for any bias in observation between the parallel and perpendicular channels for the microscopy setup, calculated with fluorescein dissolved in aqueous solution (1 µM, pH = 10), where $G=\frac{(I{par})}{(I{perp})}$ such that r = 0 in this case. A mean of multiple rectangular ROIs per image in individual I_par_ and I_perp_ channels was used to compute the value G, averaged across experiments.

### Microscopy and image acquisition

The images were acquired in Nikon Eclipse Ti2 E, with the additional module of Yokogawa CSU W1 So-Ra, with 60X (Plan-Apo N 60X, NA-1.42, oil). The FRAP images were acquired in a 60X oil objective (Plan-Apo N 60X oil, NA −1.42, oil, Olympus), optical zoom 4.5X, mounted on an Olympus IX83 inverted microscope with an attached module for laser scanning confocal - FV3000 with 488 solid state lasers used at 30% power over a circular ROI of 1 μm^2^, at 1024 × 1024 pixels. The STED images were acquired using 60X oil objective (Plan-Apo N 60X oil, NA −1.42, oil, Olympus IX83) with an attached Abberior STEDYCON system. The images for Anisotropy were acquired on an Olympus IX83 (Tokyo, Japan) with a 100X (PlanS-Apo, 1.4 NA, oil) objective and an Andor Zyla 4.2 sCMOS camera. CoolLED pE-4000 was used as the light source. An Okolab UNO (Naples, Italy) live-cell chamber supplied with humidified CO_2_. A linear sheet polarizer was placed after the excitation filter in the filter cube, aligned to polarize light in the vertical direction. An Optosplit II (Cairn Research, Kent, England) with a polarizing beam splitter was attached to the left port of the IX83 frame. The field of view was reduced using apertures and the parallel and perpendicular components of the emission light were projected on two halves of the camera chip, allowing simultaneous recording.

For pH measurements, samples were excited using a 561 nm laser, and emission was collected at 640 (700/75) and 580 nm (595/40) using the mentioned filter wheels. Since the Nikon spinning disc had a single camera, the sample was excited twice with the same laser, and the emissions were collected sequentially. The exposure setting for the excitation were 500 ms (em-640) and 3 s (em-580). These exposure settings and order of acquisition was kept constant across treatments, experiments and pH calibration.

### Image and data statistical analysis

The line profiles were made in Fiji across single plane images of the respective channels used during imaging. All analyses except the ratio measurements in the nucleolar pH assay, FRAP analysis and intensity measurement of Ki-67 were done on MATLAB using custom-made codes. Binary nuclear masks of a single plane were made using the Hoechst images, while nucleolar masks from a single plane were extracted from the 28S rRNA images (Appendix Fig. S1A). Nucleoli that were over- or under-segmented were eliminated from the analysis. The 5’ETS intensity levels were calculated using the 28S rRNA masks itself, along with the nucleolar area. The nucleolar protein intensities of UBF1 were calculated after automated segmentation of the masks created from the individual images itself since taking the nuclear mask would lead to underestimation of the mean protein levels due their sub-compartmentalization. Circularity was calculated using the in-built MATLAB function. Nucleoli per cell was calculated manually.

The Ki-67 and PARP1-chromobody-RFP intensity levels were manually calculated using customized code on MATLAB for a single plane, with freehand marking of the nucleoli (size ≥ ~1 µm diameter) to make a binary mask, using the Hoechst channel. The rest of the nucleoplasm was also calculated using Hoechst to make a binary mask of the nuclei, and then subtracting the nucleolar mask from the nuclear mask. The ratio of mean nucleolar intensity per nuclei to its corresponding nucleoplasmic intensity, and the mean nucleolar intensity was then computed.

The NPM and NCL intensity levels were calculated using a customized code on MATLAB for a single plane, where the nucleolar mask was obtained using the NPM channel and the nuclear mask was obtained from the Hoechst channel (Appendix Fig. S1B). The nucleoplasm binary mask was calculated by subtracting the nucleolar mask from the nuclear mask. The ratio of mean nucleolar intensity per nuclei to its corresponding nucleoplasmic intensity, and the mean nucleolar intensity was then computed.

The nuclear γH2AX intensity levels were calculated using a customized code on MATLAB for maximum intensity projected images, using the Hoechst channel to segment the nuclear masks and then overlaying it over the γH2AX channel, to obtain the mean nuclear γH2AX intensity.

For calculating the ring-like structure of 5’ETS and the UBF, binary masks were created from UBF images, and an annulus mask was created around the UBF mask. The intensity of UBF was calculated within the foci and at the annulus. Similarly, 5’ETS intensity in the foci and the annulus was also calculated similarly, and its ratio was plotted.

The nucleolar reorganization was quantified using a metric we designed. The cell nucleoli from the most focused plane were manually segmented from the Hoechst image, and their perimeter was marked, and perimeter coordinates were extracted. The nascent rRNA intensity levels were measured within the marked nucleoli. The FC centers marked by UBF1 in a nucleolus were segmented using the UBF images itself, and the centroid was calculated for each. The shortest distance between the individual UBF1 foci and the nucleolar periphery was then calculated. The distance thus calculated from the UBF centroid to the nucleolar periphery was annotated as positive if the foci was within the nucleolar perimeter and negative if the centroid of the foci lay outside the nucleolar periphery (Appendix Fig. S2). The nascent rRNA levels is then plotted against the average distance of the UBF1 foci from the nucleolar periphery of a given nucleolus.

For FRAP analysis, a constant circular ROI is used to mark the photobleached region and the intensity was calculated for each timepoint before, and after bleaching. The entire nucleus was marked with a freehand ROI and its intensity was also calculated similar to the bleached region. A constant background (bg) value is subtracted from the photobleached ROI (ROI-bg = I) and also from the nucleus (Nuc-bg = total). Then the FRAP index was calculated as follows: (I_(t)_/I _(0)_ * total _(t)_/total _(0)_): I_(t)_ is intensity of the bleached ROI at each timepoint, I _(0)_ is the intensity of the ROI before bleaching, total _(t)_ is the intensity of the nucleus at each timepoint and total _(0)_ is the intensity of the nucleus before bleaching). For calculation of T_1/2_, the FRAP recovery curves were plotted, and the fitting of the exponential part of the curve after the photobleaching was set to zero on Origin software, from which the values of T_1/2_ were obtained.

The 3D renders of the FCs marked by UBF in the expansion microscopy experiments were obtained using IMARIS software.

For the pH measurements, individual channels were taken after constant background subtraction and manual marking of the nucleoli was done using the freehand tool, on Cy5 channel which showed clear nucleolar signal, and the intensities were recorded using Fiji. All graphs and statistical measurements were made on Origin. To compare means, quantification was done using data from at least three independent replicates unless mentioned otherwise. The bar graphs represent the mean and standard error of the mean (SEM) unless mentioned otherwise. For single-cell distributions, a non-parametric test (Kolmogorov–Smirnov test) was employed as it does not make assumptions regarding the normality of the underlying data. Single-cell distributions may not follow a normal distribution, and a non- parametric test is more suited for these cases. *p* values below 0.05 were considered statistically significant.

### Antibodies, FISH probes, and chemicals used

The dilutions of primary and secondary antibodies used for IF are given in the Reagents and Tools Table. For certain antibodies, a higher concentration was used for expansion microscopy experiments. GFP-tagged UBF was obtained from Addgene (Addgene plasmid #17656). Probe sequences targeting rRNA were procured from Pixel Biotech GmbH with required fluorophores (Atto 488 for 5’ETS probes or Atto 565 for 28S probes, and 18S probes). The probe sequences are presented in the Reagents and Tools Table. Multiple probes were used against each target sequence. Each probe carried a single fluorophore. Details of chemical concentration and stocks used are also part of the Reagents and tools table.

## Supplementary information

Appendix

Peer Review File

Source data Fig. 1

Source data Fig. 3

Source data Fig. 4

Source data Fig. 5

Source data Fig. 6

Figure EV1 Source Data

Figure EV3 Source Data

Figure EV4 Source Data

Figure EV5 Source Data

Appendix Figure S3 Source Data

Expanded View Figures

## Data Availability

This study includes no data deposited in external repositories. The source data of this paper are collected in the following database record: biostudies:S-SCDT-10_1038-S44319-026-00932-z.
