# Supplementary material for: Nucleolar reorganization on stress depends on physicochemical changes due to reduced nascent rRNA synthesis
Source: EMBO Rep. 2026 Sep 22;27(21):6305–41. doi: 10.1038/s44319-026-00932-z (PMC13743745; doi:10.1038/s44319-026-00932-z)
Supplement: Supplementary file 1 [file 44319_2026_932_MOESM13_ESM.pdf]

Expanded View Figures

Figure EV1. Structural characterization of nucleolar sub-compartments using Expansion Microscopy and STED.

(**A**) Graph for nuclear area in unexpanded and expanded nuclei of U2OS cells. Data represents mean ± SEM, $n = 3$ for ≥60 cells, over three independent experiments. (**B**) 3D renders of expanded U2OS cell nucleoli showing three different orthogonal slices (left to right) of XY, YZ, XZ of two FC modules marked by UBF1 (magenta). Scale bar is 5 µm. (**C**) STED images of cells marking UBF1 for a single nucleolus, with 4FC centers marked in magenta boxes, and the insets shown on its right. Scale bar is 0.5 µm. (**D**) U2OS cell nucleoli after expansion, marked with Ki-67, RNAPI, NPM1 (top to bottom respectively, in magenta) co-stained with NPM1 (yellow) in each case, and merge of the two compartments on the rightmost panel. Scale bar is 10 µm. (**E**) Line profile for the merged images from (**D**). Statistical significance calculated using K.S test. Source data are available online for this figure.

▶

 © The Author(s)

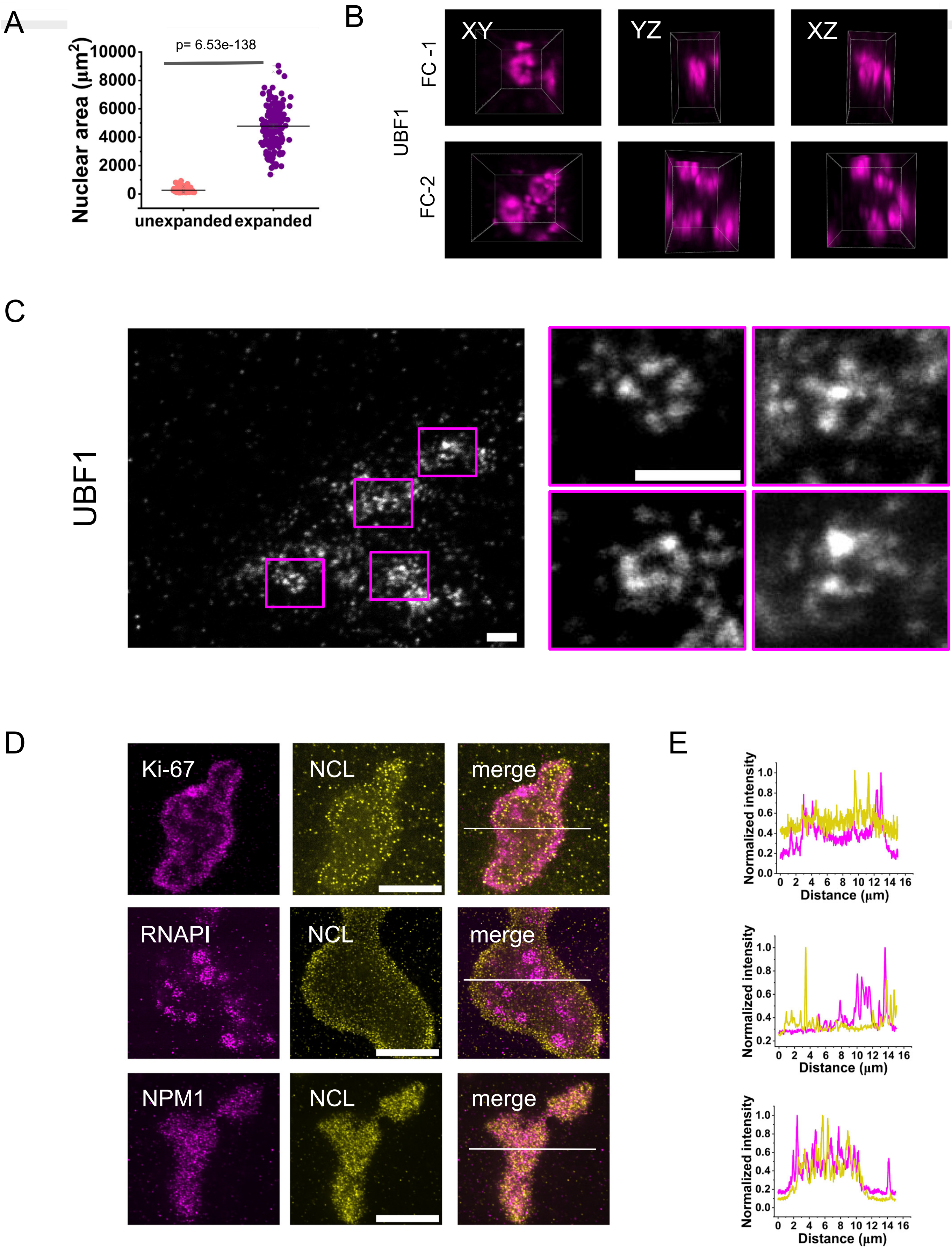
A
p= 6.53e-138
Nuclear area (µm²)
10000
8000
6000
4000
2000
0
unexpanded
expanded
B
XY
YZ
XZ
FC -1
FC-2
UBF1
C
UBF1
D
Ki-67
NCL
merge
RNAPI
NCL
merge
NPM1
NCL
merge
E
Normalized intensity
Distance (µm)

© The Author(s)

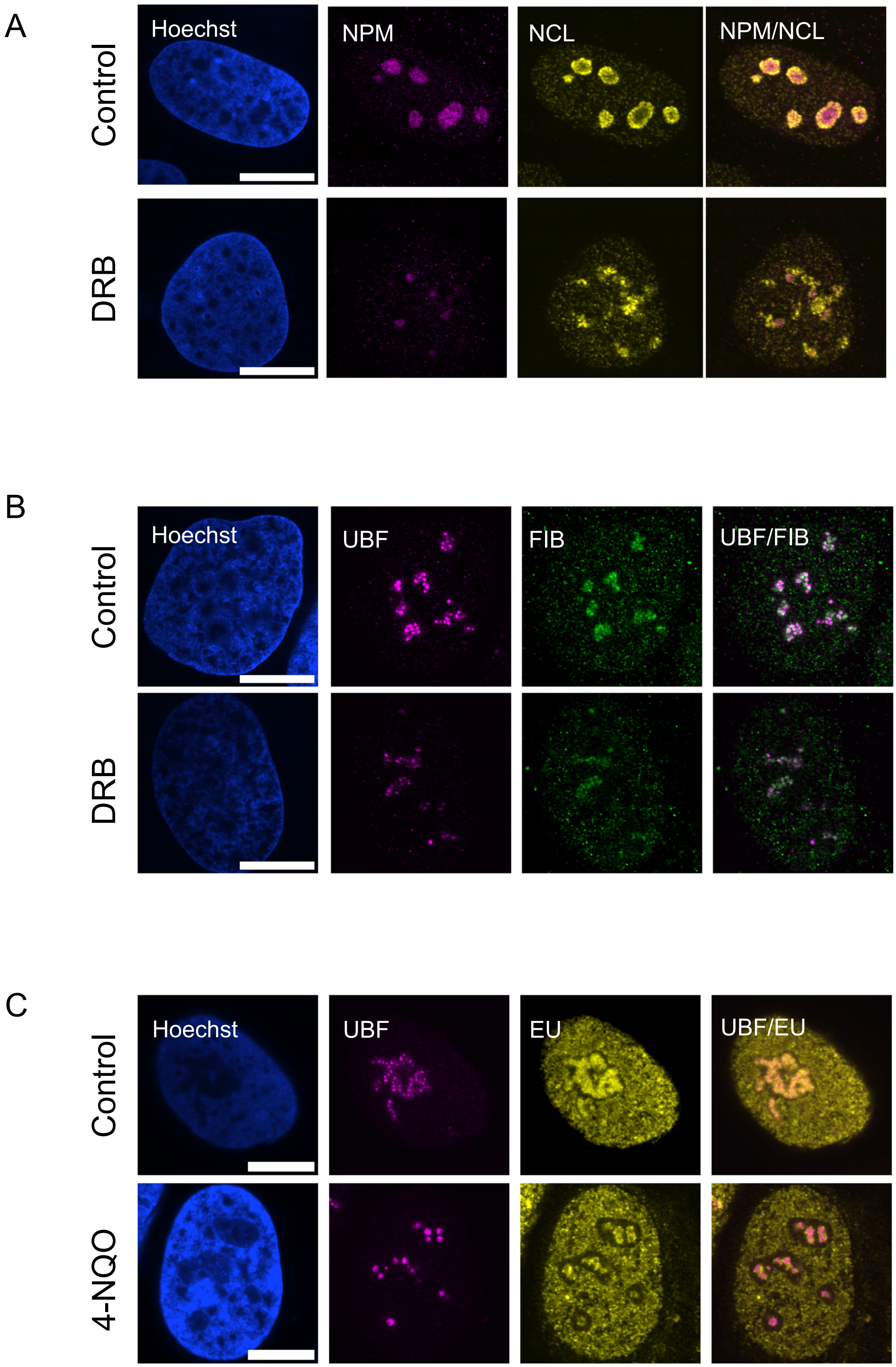
A
Control
DRB
Hoechst
NPM
NCL
NPM/NCL
B
Control
DRB
Hoechst
UBF
FIB
UBF/FIB
C
Control
4-NQO
Hoechst
UBF
EU
UBF/EU

 © The Author(s)

◀ **Figure EV2. Spatial reorganization of nucleolar proteins upon RNA Polymerase II inhibition by DRB, and rRNA reorganization upon DNA damage by 4-NQO.**

(**A**) U2OS cells treated with DMSO (control) and DRB at 60 μM, for 1 h, stained with Hoechst (1 μg/ml) and nucleoli marked with NPM (magenta), NCL (yellow), and their merge. (**B**) U2OS cells treated with DMSO (control) and DRB at 60 μM, for 1 h, stained with Hoechst (1 μg/ml) and nucleoli marked with UBF (magenta), FIB (green), and their merge. (**C**) shows cells treated with DMSO (control) and 4-NQO (1 μg/ml) for 1 h with 5-EU pulsed for the last 30 min to detect nascent transcription. It is then stained with Hoechst (1 μg/ml) and nucleoli marked with UBF (magenta), and 5-EU labeled with TAMRA azide (yellow). Scale bar is 10 μm.

© The Author(s)

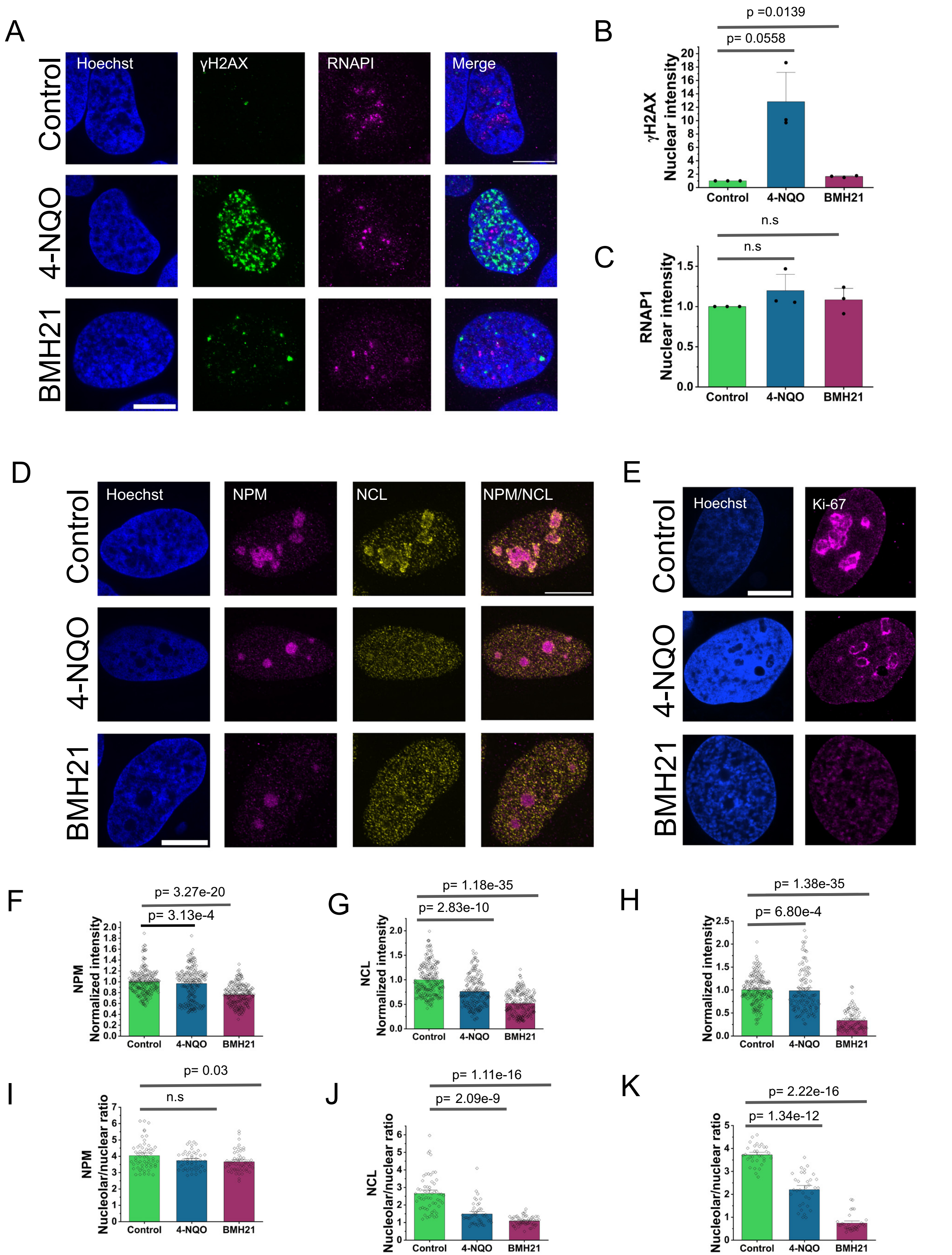
A
Control
4-NQO
BMH21
Hoechst
γH2AX
RNAPI
Merge
B
p =0.0139
p= 0.0558
γH2AX
Nuclear intensity
Control
4-NQO
BMH21
C
n.s
n.s
RNAP1
Nuclear intensity
Control
4-NQO
BMH21
D
Control
4-NQO
BMH21
Hoechst
NPM
NCL
NPM/NCL
E
Control
4-NQO
BMH21
Hoechst
Ki-67
F
p= 3.27e-20
p= 3.13e-4
NPM
Normalized intensity
Control
4-NQO
BMH21
G
p= 1.18e-35
p= 2.83e-10
NCL
Normalized intensity
Control
4-NQO
BMH21
H
p= 1.38e-35
p= 6.80e-4
Normalized intensity
Control
4-NQO
BMH21
I
p= 0.03
n.s
NPM
Nucleolar/nuclear ratio
Control
4-NQO
BMH21
J
p= 1.11e-16
p= 2.09e-9
NCL
Nucleolar/nuclear ratio
Control
4-NQO
BMH21
K
p= 2.22e-16
p= 1.34e-12
Nucleolar/nuclear ratio
Control
4-NQO
BMH21

 © The Author(s)

Figure EV3. Effects of DNA damage by 4-NQO and transcriptional inhibition by BMH21 on RNA Polymerase I levels and nucleolar protein translocation.

U2OS cell nuclei from cells treated with 4-NQO (1 μg/ml) for 1 h and BMH21 (2 μM) for 3 h, compared to control nuclei. (**A**) representative cell nuclei from cells stained with Hoechst (blue, 1 μg/ml), γH2AX (green) and RNAPI (magenta) and their merge. (**B**) Mean nuclear intensity of γH2AX and (**C**) RNAPI plotted from three independent experiments. Each dot represents the average of the individual experiments (each experiment normalized to its control). Statistical significance calculated using a *t*-test. The significance for (**C**) shows higher between control and 4-NQO due to the higher standard deviation among the experiments, despite the mean being significantly different. (**D**) shows nuclei from cells treated with 4-NQO (1 μg/ml) for 1 h and BMH21 (2 μM) for 3 h, compared to control nuclei. Cells are stained with Hoechst (blue, 1 μg/ml), NPM (magenta) and NCL (yellow) and their merge. (**E**) shows nuclei from cells treated with 4-NQO (1 μg/ml) for 1 h and BMH21 (2 μM) for 3 h, compared to control nuclei. Cells are stained with Hoechst (blue, 1 μg/ml) and an antibody against Ki-67 (magenta). (**F**) Normalized nucleolar intensity of NPM, (**G**) NCL and (**H**) Ki-67 plotted. Graph plotted for the ratio between average nucleolar to nuclear (nucleoplasmic) intensity of (**I**) NPM, (**J**) NCL, and (**K**) Ki-67 per cell. Data represents mean ± SEM, with $n = 58; 48; 57$ nuclei (**D**, **F**, **G**, **I**, **J**) and $n = 32; 34; 33$ nuclei (**E**, **H**, **K**) over three independent experiments. Statistical significance calculated using the K.S test. Scale bar is 10 μm. Source data are available online for this figure.

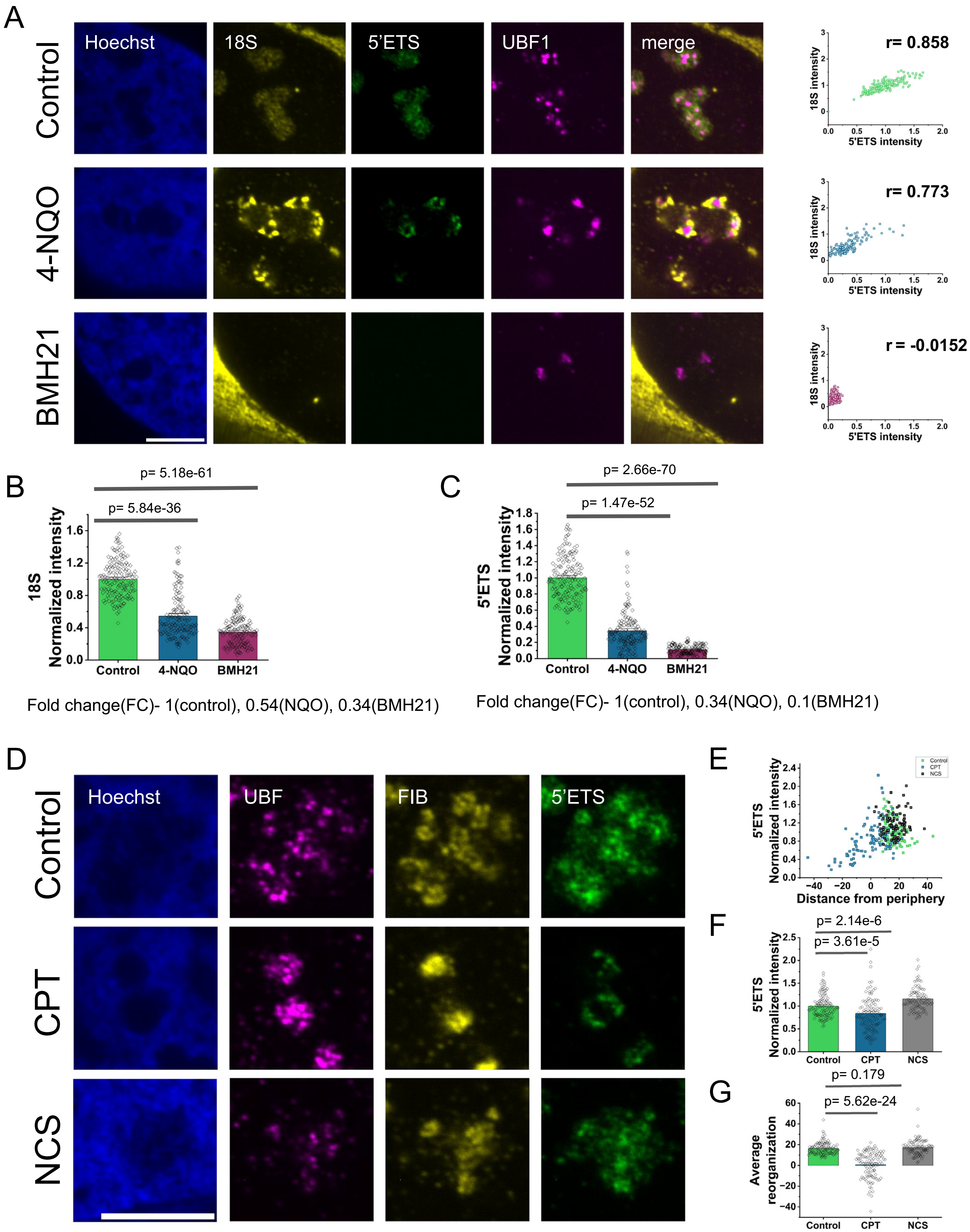

Fold change(FC)- 1(control), 0.54(NQO), 0.34(BMH21)

Fold change(FC)- 1(control), 0.34(NQO), 0.1(BMH21)

© The Author(s)

Figure EV4. Changes to SSU rRNA levels and nucleolar reorganization in response to different stressing agents.

U2OS cell nuclei from cells treated with 4-NQO (1 μg/ml) for 1 h and BMH21 (2 μM) for 3 h, compared to control nuclei. (**A**) representative cell nuclei from cells stained with Hoechst (blue, 1 μg/ml), probed with 18S rRNA(yellow), 5′ETS rRNA(green), and stained with an antibody against UBF1 (magenta) and merged, with a scatter plot for correlation between 18S rRNA and 5′ETS, showing the normalized intensity for each treatment beside it. Pearson's correlation coefficient (r) is mentioned on the graph. The correlation is significant for control and 4-NQO and non- significant for BMH21 at $p < 0.0001$. (**B**) Normalized intensity of 18S rRNA. (**C**) Normalized intensity of 5′ETS rRNA. Data represents mean ± SEM, with mean labeled for each condition. $n \geq 150$ (165; 150; 158) nucleoli over three independent experiments. Fold changes (FC) with respect to control is 0.54 for 4-NQO and 0.34 for BMH21 in (**B**), while it is 0.34 for 4-NQO and 0.1 for BMH21 in (**C**). (**D**) Cells treated with camptothecin (CPT, 25 μM, 1 h), and neocarzinostatin (NCS, 1 μg/ml, 1 h) compared to control in DMSO. Cells are stained with Hoechst (blue), UBF1 (magenta), Fibrillarin (yellow) and probed for 5′ETS rRNA (green). (**E**) Mean 5′ETS intensity plotted against the mean distance of the UBF1 foci from the nucleolar periphery (in pixel units). Note how CPT data points show both lower 5′ETS intensity and lower distance values indicative of reorganization, while the NCS data points effectively overlap with the control. (**F**) Normalized 5′ETS intensity from (**E**). (**G**) Average distance from the nucleolar periphery from (**B**). Data represents mean ± SEM. Statistical significance calculated using the K.S. test, where $n \geq 95$ nucleoli across three independent experiments. While NCS data also turns out to be significantly different in a K.S test, given the high nucleoli numbers, the mean values are quite comparable to the control cells. This is also seen in the scatter plot. Scale bar is 1 μm. Source data are available online for this figure.

© The Author(s)

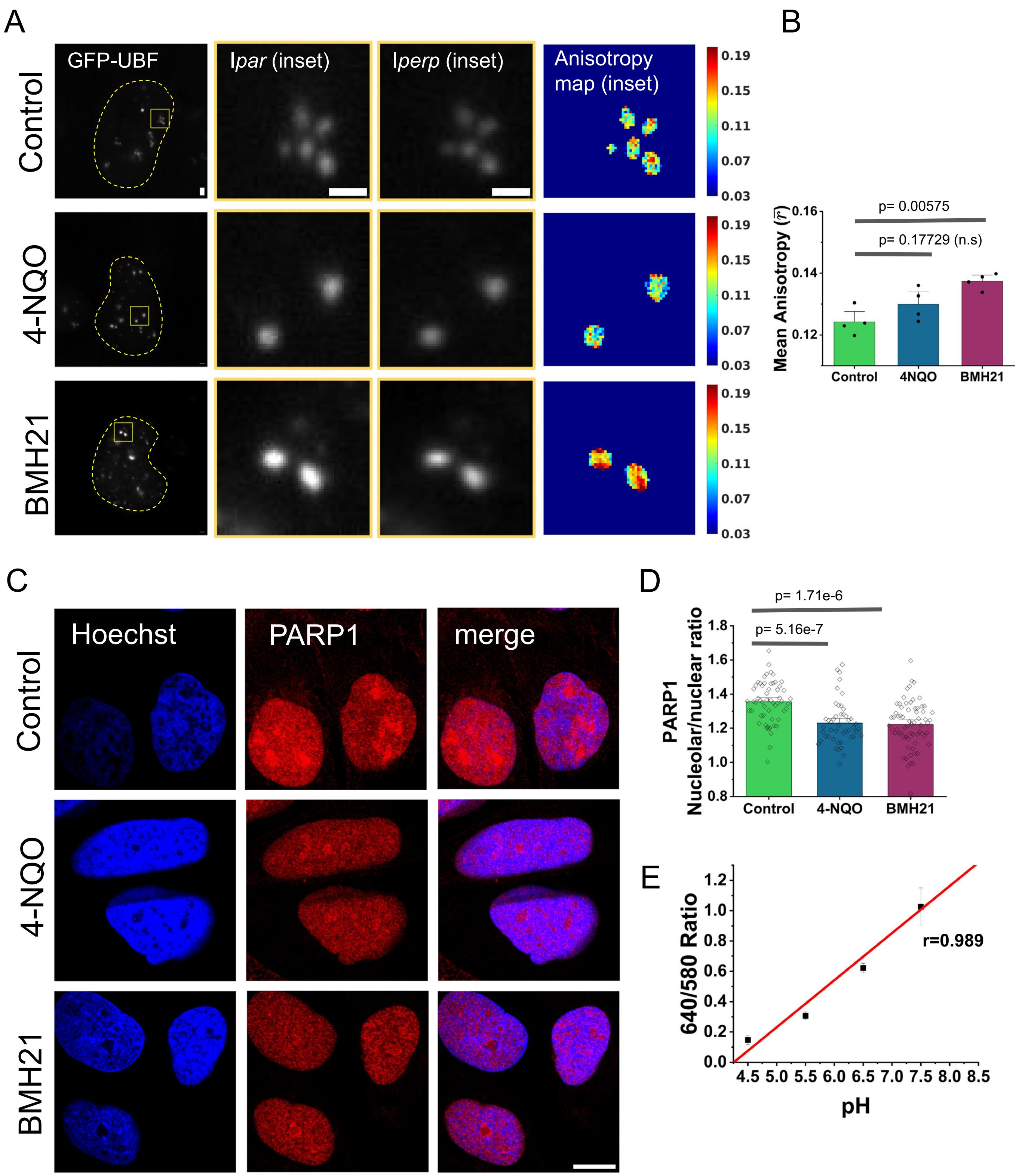
A
Control
4-NQO
BMH21
GFP-UBF
Ipar (inset)
Iperp (inset)
Anisotropy map (inset)
0.19
0.15
0.11
0.07
0.03
B
Mean Anisotropy
p= 0.00575
p= 0.17729 (n.s)
0.16
0.14
0.12
Control
4NQO
BMH21
C
Hoechst
PARP1
merge
Control
4-NQO
BMH21
D
PARP1 Nucleolar/nuclear ratio
p= 1.71e-6
p= 5.16e-7
1.8
1.6
1.4
1.2
1.0
0.8
Control
4-NQO
BMH21
E
640/580 Ratio
r=0.989
1.2
1.0
0.8
0.6
0.4
0.2
0.0
4.5 5.0 5.5 6.0 6.5 7.0 7.5 8.0 8.5
pH

 © The Author(s)

Figure EV5. Rotational dynamics of GFP-UBF and stress-induced relocalization of PARP1 upon DNA damage and transcriptional inhibition.

(**A**) U2OS cells were transfected with GFP-UBF. Representative nuclei from cells treated with 4-NQO (1 μg/ml, 1 h) and BMH21 (2 μM, 3 h) compared to control nuclei. Compared to control nuclei are shown in the leftmost column. The nuclear boundaries are drawn with a dotted line, and zoomed-in images of the nucleoli in the inset boxes are shown on the right. The parallel (Ipar) and perpendicular (Iperp) components of the emission light are collected and used to compute the anisotropy maps. Anisotropy maps of the representative nucleoli are shown in the rightmost columns. (**B**) Average of the mean anisotropy values of nucleolar GFP-UBF foci. Data represents mean ± SEM, $n \geq 200$ (225; 217; 230) nucleoli over four independent experiments. Statistical significance calculated using a *t*-test. Scale bar is 10 μm. (**C**) U2OS cells stably transfected with a PARP1-chromobody-RFP (red) and stained with Hoechst (blue) allows the visualization of endogenous PARP1. Higher nucleolar intensity is seen in control cells, which is lost following 4-NQO and BMH21 treatment. (**D**)Ratio of mean nucleolar intensity to nucleoplasmic intensity of PARP1-chromobody-RFP are shown, $n = 57$, 47, 66 nuclei over three independent experiments. Data represents mean ± SEM, with statistical significance tested using the K.S test. Scale bar is 10 μm. (**E**) pH calibration curve for SNARF-4F. Data represents mean ± standard deviation (SD), $n \geq =277$ nucleoli over three independent experiments. Source data are available online for this figure.
