# Supplementary material for: Nucleolar reorganization on stress depends on physicochemical changes due to reduced nascent rRNA synthesis
Source: EMBO Rep. 2026 Sep 22;27(21):6305–41. doi: 10.1038/s44319-026-00932-z (PMC13743745; doi:10.1038/s44319-026-00932-z)
Supplement: Supplementary file 2 [file 44319_2026_932_MOESM1_ESM.pdf]

Appendix Figures

Title: Nucleolar reorganization on stress depends on physicochemical changes due to reduced nascent rRNA synthesis

Table of contents

Contents	Page number
Appendix Figure S1	2
Appendix Figure S2	3
Appendix Figure S3	4

Appendix Figure S1

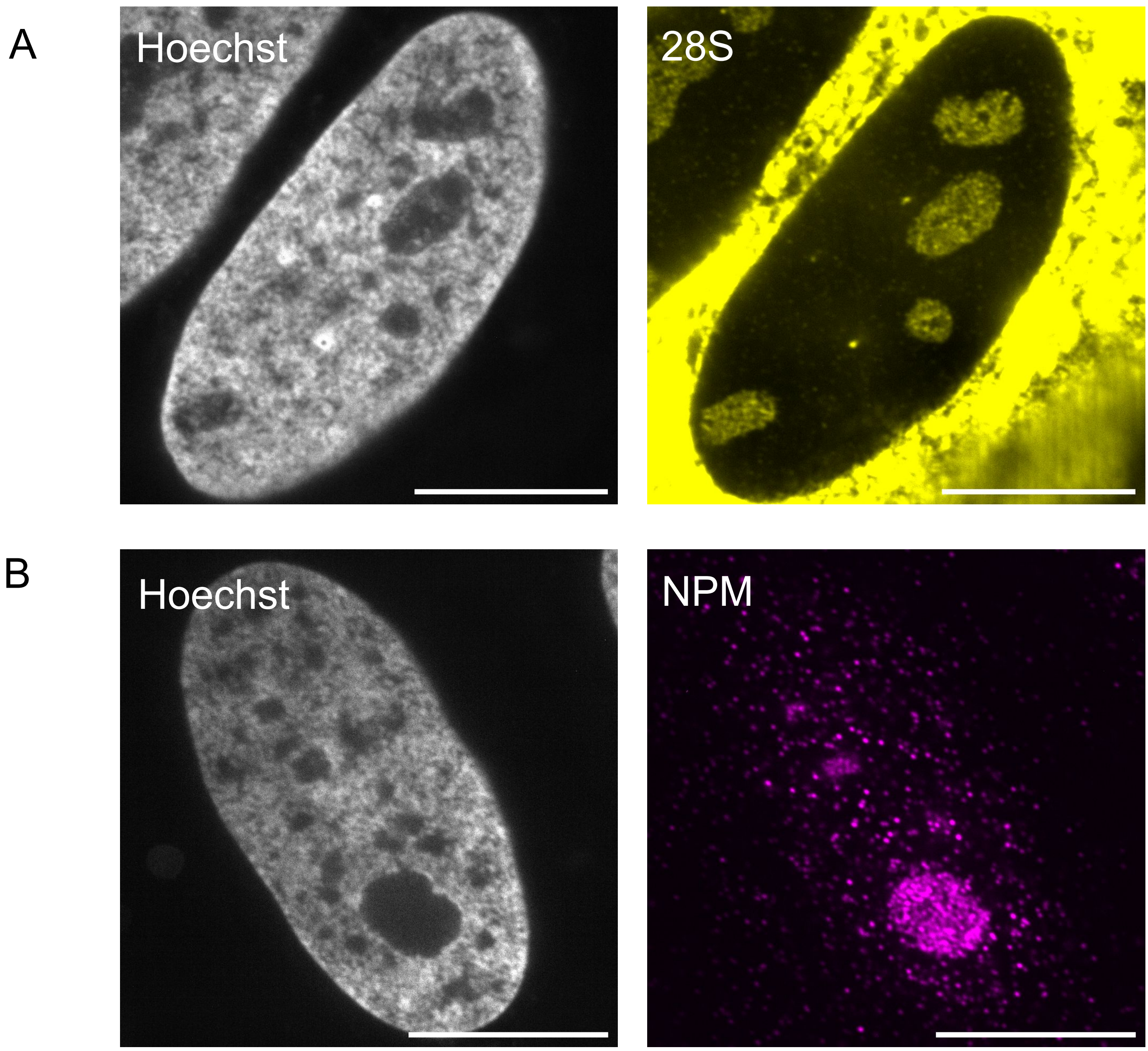

Appendix Figure S1: The Hoechst-depleted regions colocalize with the nucleolar 28S rRNA or NPM marking the GC. For experiments where 28S or NPM is present we use the 28S or NPM channel to segment the nucleolus, else we use Hoechst channel to manually mark the nucleoli using the hoechst depleted zone as a visual marker as shown in appendix figure .Scale bar is 10 μm

Appendix Figure S2

A

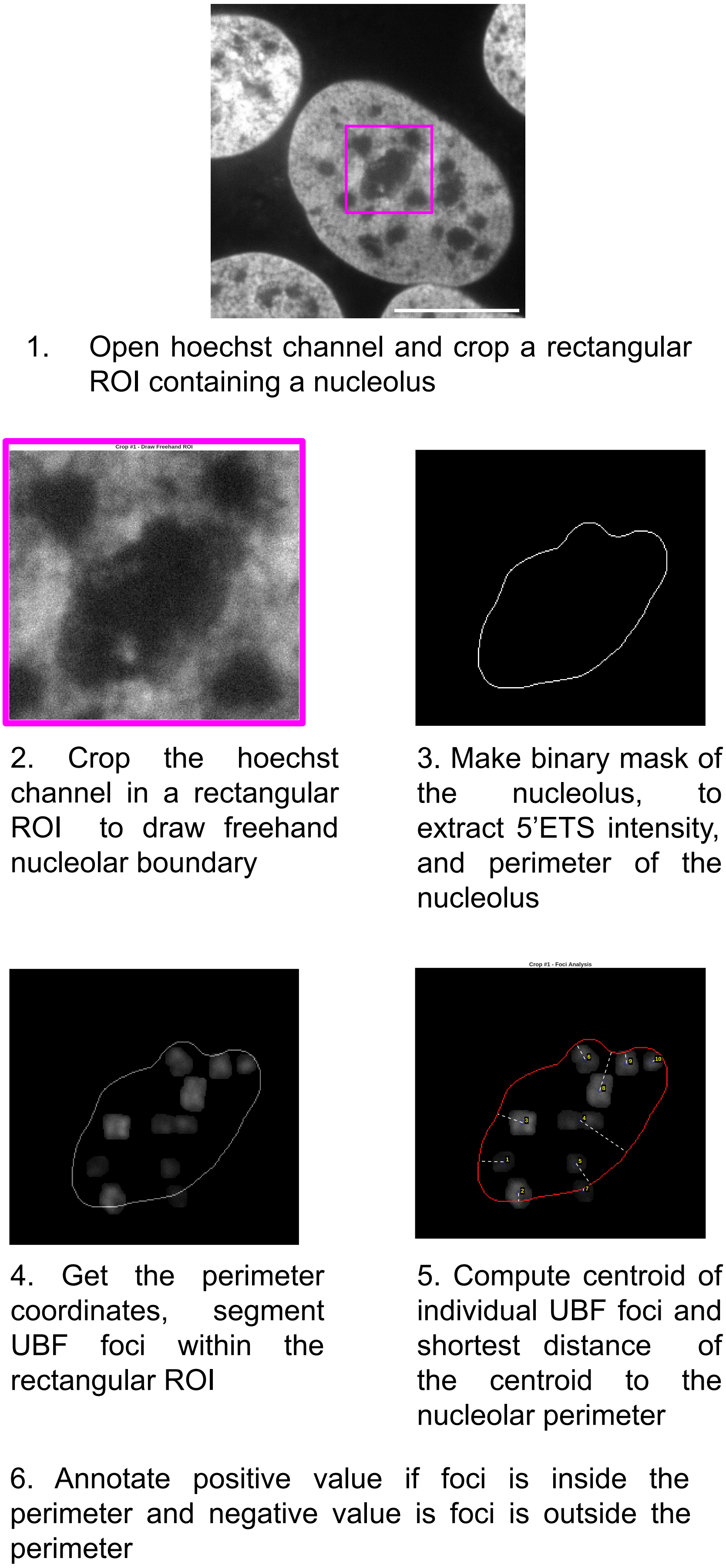

Appendix Figure S2: Analysis pipeline of the reorganization metric for UBF and 5’ETS using custom MATLAB code on the most focused plane of the image stack.(ROI- region of interest). Scale bar in (1) is 10 μm

Appendix Figure S3

A

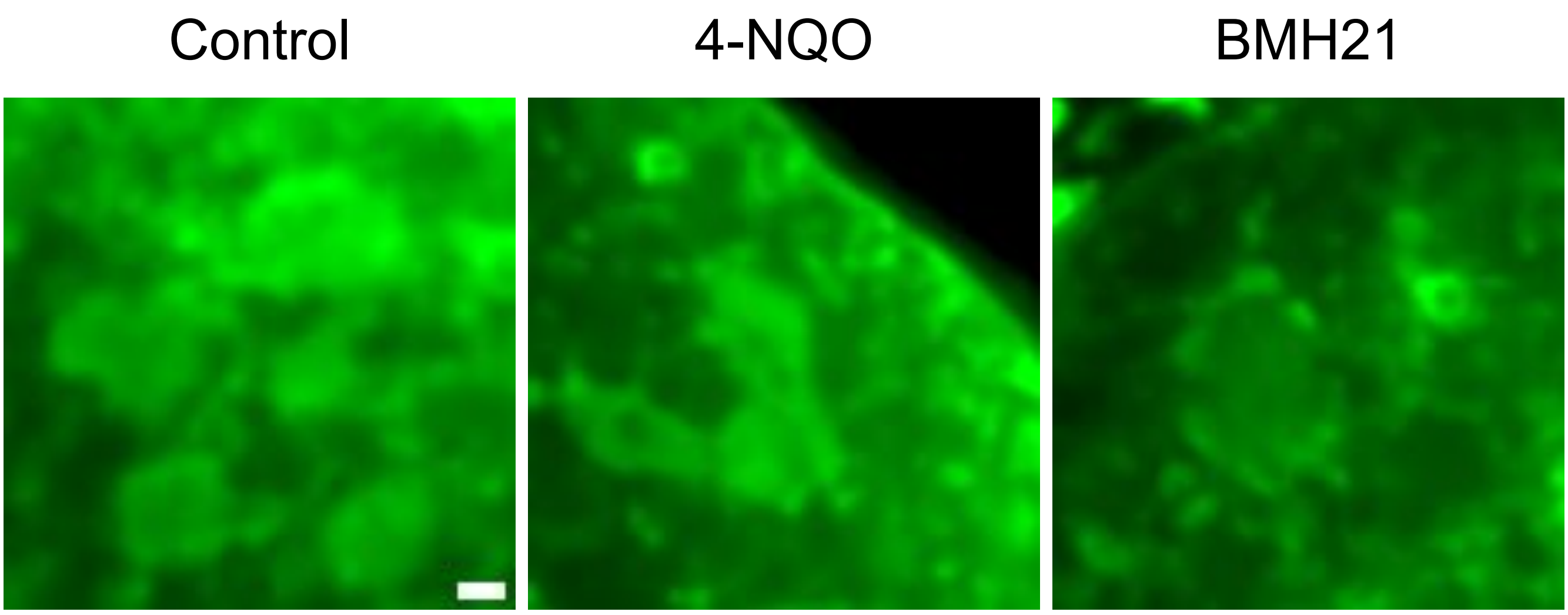

B

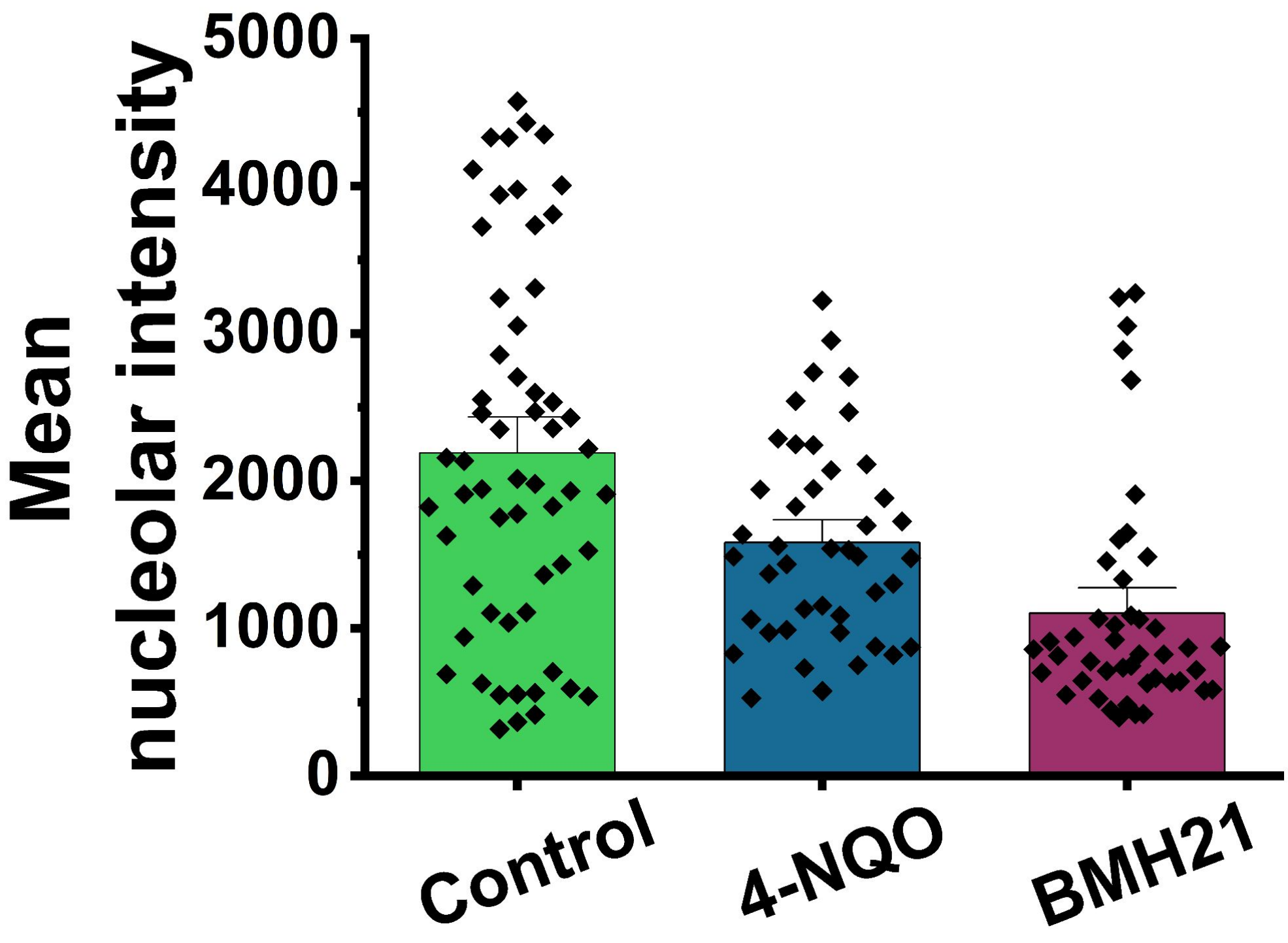

Appendix Figure S3: (A) U2OS cells incubated with pHrodo-AM(1:1000) for 30 minutes. (B) Mean nucleolar intensity of pHrodo-AM. Data represents mean±S.E.M, for a single experiment,with n= 58,43,45 nucleoli.
