# Supplementary material for: Nucleolar reorganization on stress depends on physicochemical changes due to reduced nascent rRNA synthesis
Source: EMBO Rep. 2026 Sep 22;27(21):6305–41. doi: 10.1038/s44319-026-00932-z (PMC13743745; doi:10.1038/s44319-026-00932-z)
Supplement: Supplementary file 3 [file 44319_2026_932_MOESM2_ESM.pdf]

EMBO reports

Nucleolar reorganization upon stress depends on physicochemical changes due to reduced nascent rRNA synthesis

Sinjini Ghosh, Souvik Sen, and Aprotim Mazumder

Corresponding author(s): Aprotim Mazumder (aprotim@tifrh.res.in)

Review Timeline:

Submission Date:	31st Dec 25
Editorial Decision:	24th Feb 26
Appeal Received:	27th Feb 26
Editorial Decision:	10th Mar 26
Revision Received:	30th Jun 26
Editorial Decision:	5th Aug 26
Revision Received:	14th Aug 26
Accepted:	31st Aug 26

Editor: Esther Schnapp

Transaction Report:

(Note: With the exception of the correction of typographical or spelling errors that could be a source of ambiguity, letters and reports are not edited. Depending on transfer agreements, referee reports obtained elsewhere may or may not be included in this compilation. Referee reports are anonymous unless the Referee chooses to sign their reports.)

23rd Feb 2026
Dear Prof. Mazumder,

Thank you for the submission of your manuscript to EMBO reports. We have now received the enclosed referee reports on it. I am sorry to say that the evaluation of your study is not a positive one.

As you will see, while referee 2 is more positive, both referees 1 and 3 point out that the data are not sufficiently novel, and that further insight into how the biophysical parameters contribute to the regulation of nucleolar architecture would be required for the publication of the ms by EMBO reports. The referees also remark that references to previous findings are sometimes incorrect.

Given these comments from 3 experts in the field, I am sorry to say that we cannot offer to publish your ms.

While we cannot pursue this manuscript further, we encourage you to transfer your study to our not-for-profit open-access sister journal, Life Science Alliance (LSA). We shared your manuscript and the accompanying reviews with LSA Executive Editor, Tim Fessenden, who is interested in these findings. He is pleased to offer consideration of this manuscript at LSA pending the following revisions:
- Adjust citations and discussion of prior literature to address the concerns of Reviewers 2 and 3.
- Adjust claims of novelty and situate the present findings more carefully as sought by all reviewers.
- Moderate claims on the role of rRNA by acknowledging the limitations of the RNA probe as sought by Reviewers 1 and 3.
- Provide evidence of DNA damage and a calibration curve for pH measurements sought by Reviewer 2.

We understand that such a revision might need to be re-reviewed, in which case Dr. Fessenden will walk the Reviewers through our transfer process. We encourage you to contact Dr. Fessenden at t.fessenden@life-science-alliance.org to discuss this work and the revisions requested. You may use the link below to immediately transfer your manuscript to LSA. You do not need to revise the manuscript before transferring it to LSA. Once you transfer, you will receive an invitation to revise and resubmit. Again please contact Dr. Fessenden if you have any questions about the LSA journal, the transfer process, or the revisions requested.

I am sorry that I cannot be more positive for EMBO reports this time, and hope that you view the possibility to transfer your study to LSA favourably.

Yours sincerely,

Esther Schnapp, PhD
Senior Editor
EMBO reports

Referee #1:

This manuscript uses expansion microscopy to resolve sub-nucleolar architecture and supports a model in which fibrillar center (FC) and dense fibrillar component (DFC) domains are organized as nested shells that reorganize under DNA damage or RNA polymerase I (RNAPI) inhibition. The authors further propose that stress-induced transcriptional downregulation remodels sub-nucleolar compartments from a viscoelastic state into more solid-like condensates, accompanied by perturbation of the nucleolar pH gradient due to loss of an rRNA scaffold. Based on imaging and quantitative spatial metrics, the authors claim that nucleolar reorganization arises primarily from reduced rRNA levels and consequent biophysical restructuring, rather than from active DNA damage signaling to facilitate rDNA repair.
While the study is technically sound, the methodology has already been used to characterize nucleolar ultrastructure and stress-induced reorganization (PMID: 40391829). In the present work, the use of expansion microscopy is confined to protein imaging and does not include concurrent visualization of RNAs, which may limit the extent of new insights compared with previous reports. Moreover, the conclusion that nascent rRNA levels influence nucleolar reorganization under stress conditions has been explored in previous studies (PMID: 35978024). Finally, while the study presents extensive descriptive data, the mechanistic framework supporting several key conclusions remains incomplete. Additional experiments providing direct evidence would help to strengthen the causal interpretation of the results.

Major concerns:
1. The structural features of FC-DFC organization revealed by expansion microscopy (Figures 1-2), including nested shell-like arrangements, have largely been established previously using various super-resolution approaches (SIM, STED, and related methods). While the imaging quality is high, these observations alone do not substantially advance current understanding of nucleolar architecture. To strengthen the novelty, the authors should either provide additional new structural insights beyond what has already been reported, or temper their claims accordingly.

2. The manuscript treats 4-NQO (DNA damage) and BMH21 (RNAPI inhibition) as perturbations that converge on reduced rRNA

synthesis. However, these are biologically distinct stresses that engage different signaling pathways and cellular responses. The current experimental design does not adequately disentangle effects of DNA damage from those of transcriptional shutdown. Without a cleaner experimental separation (e.g., transcriptional shutdown without DNA damage, or DNA damage that spares transcription), the interpretation remains ambiguous.

3. The FRAP experiments on GFP-UBF are appropriate to probe nucleolar dynamics, and the observed mild recovery changes after 4-NQO versus pronounced recovery loss after BMH21 are consistent with RNA-dependent modulation of condensate fluidity. However, FRAP alone is insufficient to conclusively demonstrate a transition from a viscoelastic to a solid-like state. Additional biophysical evidence would be required to support this claim.

4. It may not be sufficiently rigorous to simply use 28S rRNA probes and 5' ETS probes as direct surrogates for mature and nascent rRNAs, respectively. 28S rRNA probes may also detect unprocessed rRNA precursors, indicating that the imaging signals represent a mixture of both mature and nascent rRNAs. In addition, 5' ETS is a part of SSU pre-rRNA, whereas 28S rRNA is a part of LSU pre-rRNA, reflecting two distinct rRNA maturation pathways. Consequently, changes in signal intensity upon stress treatment cannot be interpreted unambiguously as changes in the absolute abundance of nascent versus mature rRNAs. Instead, these differences may simply reflect altered processing kinetics between SSU and LSU pre-rRNAs. To strengthen the interpretation, it would be necessary to include probes targeting 18S rRNA as an additional control to more appropriately distinguish rRNA maturation states.

5. Both 4-NQO and BMH21 reduce rRNA expression level and induce nucleolar proteins shuttling, with BMH21 exerting even stronger effects. However, only the 4-NQO induces deacidification, the manuscript does not provide a mechanistic explanation for the observed differences in nucleolar pH between these two conditions. More broadly, this study would benefit from a clearer mechanistic explanation linking changes in biophysical properties to nucleolar organization and function. While the presented measurements provide valuable descriptive information, they are largely correlative, and additional mechanistic insight would be required to clarify how these biophysical parameters contribute to the regulation of nucleolar architecture.

Minor concerns:
1. In Figure 3D, the labeling of UBF and rRNA appears to be reversed, which does not match the description in the main text.

2. In Figure 4, panel D is referenced in the text, but no Figure 4D is present.

Referee #2:

In this study, the authors utilize expansion microscopy to resolve changes in nucleolar architecture in response to DNA damage and transcription inhibition. They show that both DNA damage and inhibition of DNA synthesis alter nucleolar structure by reorganizing nucleolar layers and affecting transcriptional activity. In addition, the authors report that DNA damage or transcriptional inhibition is accompanied by an increase in nucleolar pH. Taken together, these results suggest that changes in nucleolar structure are largely driven by alterations in rRNA dynamics.

Overall, the manuscript is well written, and the images are well presented and clearly described. While some of the observations reported here have been previously described in the field, from this reviewer's perspective the data are compelling and provide an orthogonal approach through which models and insights into nucleolar biology can be tested. As such, and regardless of some overlap with existing literature, I strongly support publication of this study in EMBO. However, while the findings are of interest, the overall scope appears more appropriate for a short report than a full article. Overall, this study clarifies and adds important new insights to the field. Although I cannot evaluate the technical details of the expansion microscopy itself, from a nucleolar and cell biology standpoint this study is conceptually significant and merits publication. That said, there are several concerns that should be addressed by the authors prior to publication.

Major Concerns:

- Several statements throughout the manuscript are misleading or inaccurate. While I highlight specific examples below, the authors should exercise greater care in how they present arguments about the field. The fact that a possibility has been proposed in a prior study does not constitute proof. Clear distinctions must be made between what is hypothesized, what is shown, and what is experimentally demonstrated. The presence of a citation alone should not be treated as a proxy for established ground truth.
- the authors claim to perform experiments under conditions of DNA damage; however, the manuscript does not provide direct evidence that DNA damage is actually occurring. At a minimum, the authors should demonstrate induction of γH2AX foci or provide other markers of DNA damage response pathway activation to substantiate these claims.
- The authors should perform a calibration curve for the pH measurements presented in Figure 6 or, alternatively, validate their findings using an independent chemical pH sensor. Such validation is necessary to strengthen confidence in the reported changes in nucleolar pH.

Other Comments:

• Please revised this misleading statement "However, the effects of cellular stress or disrupted transcription on nucleolar protein and RNA organization are still not well understood, limited both by the resolution of light microscopy, and lack of tools for detection of transcript species of interest and complex sample preparation in electron microscopy." Indeed, this an unresolved question. But the issue is resolution in general and not necessarily limited by resolution of light microscopy, the issue is more complex than that.
• Please note that the following statement is inaccurate and misleading "The outflux of newly synthesized rRNA is facilitated by a combined action of K-block and D/E tracts enriched nucleolar proteins. The K-blocks create a scaffold of transient multivalent interactions that aid in nucleolar organization, while the D/E tracts serve as proton carriers that set up a pH gradient, both contributing to the directional export of processed rRNA." The outflux of rRNA is more complex than described and emerging studies suggest that K-block and D/E tracks containing-proteins contribute. However, this statement ignores the multitude of other process, molecules and biochemical reactions that contribute to this process overall. Therefore, this statement needs to be revised to reflect the facts and what is known and what is just a hypothesis. Similarly, it has been suggested that D/E tracks might serve as proton carriers, but this has not been formally proven and so it should not be state as ground truth.
• This statement needs references "suggested that the rRNA transcription is initiated at the interface of FC-DFC with co-transcriptional processing, triggering phase separation and rRNA sorting into the subsequent compartments." This has been the prevailing view in the field for the last decade.
• This statement is completely FALSE/Disproven "It is known that the rRNA leads to the nested tripartite structure of nucleoli(Farley et al, 2015; van Sluis & McStay, 2017) and direct or indirect inhibition of transcription leads to the nucleolar reorganization leading to its segregation or reoriented bipartite structure." The bipartite structure of the nucleolus is defined as the nucleoli structure in non-amniotes organism. It has absolutely nothing to do with the nucleolus under stress. Secondly, three reports have shown that the tripartite structure of the nucleolus is mediated by Treacle.
• This statement is in principle inaccurate please revise "Condensates formed by RNA or protein or both, get seeded by upon increasing concentration and affinity towards each other"
• This statement is inaccurate and misleading please revised "This is because rRNA can scaffold multivalent interaction among the Glycine-Arginine rich (GAR) resident proteins which, when altered, shifts to GAR-GAR interacting condensates making it more viscous or dense in nature"

Referee #3:

In their manuscript "Nucleolar reorganization on stress depends on physicochemical changes due to nascent rRNA synthesis" the authors use advanced fluorescence microscopy techniques (IF, RNA FISH, FRAP, fluorescence pH sensor in combination with expansion microscopy and SoRa super-resolution imaging) to observe changes in the nucleolus, particularly after treatment with 4-NQO and BMH-21. 4-NQO forms covalent adducts at purine bases, triggering a DNA damage response that indirectly leads to Pol I inhibition. BMH-21 acts as an intercalator of GC-rich DNA without a DNA damage response. Unlike 4-NQO and other Pol I inhibitors such as CX-5461, it causes rapid dissociation of Pol I from rDNA and subsequent proteasome-dependent degradation of the catalytic RPA194 subunit within 2 to 3 hours.

Although the topic is interesting, the claims often lack novelty and originality, and in some cases they are supported more convincingly by previously published results than by the authors' own findings (e.g. "Stress-induced transcription inhibition remodels the sub-nucleolar compartments from a viscoelastic state into solid-like condensates thereby perturbing the nucleolar pH gradient due to the missing rRNA scaffold" in the Abstract). The Title and Abstract promise a significant deepening of the mechanistic understanding of nucleolar reorganization under stress conditions. In fact, the general term "stress" mainly encompasses treatments with the two inhibitors (without systematically comparing them with other DDR-inducing or DDR-independent Pol I inhibitors). Monitoring of physicochemical changes is limited to pH measurements. The role of nascent RNA synthesis is extrapolated from FISH localization studies on pre-rRNA. Another important general criticism is that the context provided by the earlier literature is not adequately presented. Essential information available in the cited literature is not always correctly mentioned or is not detailed enough.

Given the complementary nature of the findings to previous studies (in particular these cited works:
Yao et al, 2019, Mol Cell. Nascent Pre-rRNA Sorting via Phase Separation Drives the Assembly of Dense Fibrillar Components in the Human Nucleolus;
Quinodoz et al, 2025, Nature. Mapping and engineering RNA-driven architecture of the multiphase nucleolus - the reference has to be properly formatted in the References;
Alley et al, 2025, ACS Chem Biol.Expansion Microscopy Provides Nanoscale Insight into Nucleolar Reorganization and Nuclear Foci Formation during Nucleolar Stress)
and the lack of in-depth analyses with orthogonal validations, the manuscript would be more suitable for a specialised cell biology journal than for EMBO Reports, which is why I cannot recommend it for publication.

Major points

1. The Introduction section should provide an appropriate introduction to various forms of nucleolar stress and clearly state the stress inducers used in the study (4-NQO and BMH-21) and their mechanism of action. Two recent review articles may be helpful here:
Imamura R, Yasuhara T. Nucleolar Organization in Response to Transcriptional Stress. Cancer Sci 116, 2649-2656 (2025).
Cerezo EL, Henry Y, Henras AK, Romeo Y. Ribosome biogenesis and homeostasis: in the front line to cope with cellular stress. Biochimie 238, 43-56 (2025).
Furthermore, it must be clarified and emphasized that only the first steps of ribosome synthesis take place in the nucleolus and that pre-ribosomal particles undergo further maturation steps in the nucleoplasm and cytoplasm (see Vanden Broeck and Klinge 2024 from the cited literature). Also the statement on page 8 "The modified RNA then diffuses to GC where they assemble with the ribosomal subunits to form mature ribosomal subunits (Dogra & Kriwacki, 2025)." has to be corrected. The multistep process of ribosome assembly is not completed in the GC.

2. The authors state on pages 4 and 7:
"But how the rRNA outward flux is spatially altered within the nucleolar sub-compartments in healthy versus stressed cells is still elusive,..."
"Although it is known that transcriptional inhibition and DNA damage leads to nucleolar reorganization(Boulon et al., 2010; Shav-Tal et al, 2005; Thomas Haaf, (1996); van Sluis & McStay, 2017), how the rRNA synthesis and maturation impact nucleolar phases and gate the outward rRNA flux is poorly understood."

I cannot agree with this, as the publication 'Mapping and engineering RNA-driven architecture of the multiphase nucleolus' by Quinodoz et al. 2025 in Nature revealed a considerable amount of detail using complementary experimental approaches (SRM + NGS) at a very high technical level. They used chemical inhibition of rRNA processing (FVP) and, above all, a series of genetic inhibitions that provide clearer insights into the factors and mechanisms involved.

3. The authors write on page 6:
"A very recent study has indeed reported the use to expansion microscopy to study nucleolar structures (Alley et al., 2025), but beyond just reporting the structural details under normal homeostasis, we further investigated how it is reorganized under stress and the roles of rRNA in such reorganization"

The results related to signal intensity and localization studies of rRNA using RNA-FISH of the 5'-ETS and 28S probes do not explain the role of rRNA in stress-dependent reorganization of the nucleolus. Although they may be related, this also applies to numerous other nucleolar factors that were not investigated in the experiments and could be causal. A particularly interesting candidate would be RPA194, which is rapidly degraded when treated with BMH-21, but not when treated with other Pol I inhibitors.
It should also be emphasized that Alley 2025 also applied stress.: "We investigate time-dependent changes in nucleolar structure and function under nucleolar stress induced by oxaliplatin, actinomycin D, and other platinum-based compounds."

4. On page 9, part of the text is very vague and partly incomprehensible:
"UBF1 is in itself a 3D-shell like structure" probably refers to the 3D structure of individual active rDNA units bound with UBF, rather than the structure of UBF1 itself.
The meaning of this sentence is unclear:
"Co-stained FC-DFC modules (using UBF1 and FIB) along with 5'ETS to understand the spatial localization of the nascent pre-rRNA showed that the pre-rRNA partially
overlapped with FIB seen in the images as well as the line profile (Figure 3E, F)."
The causal relationship between the two parts of the next sentence is not substantiated by the data provided: "The partial interspersion of 5'ETS with FIB signals (Figure 3F) suggested that the rRNA transcription is initiated at the interface of FC-DFC with co-transcriptional processing, triggering phase separation and rRNA sorting into the subsequent compartments."
Thorough analyses using shRNA-mediated depletion of 17 factors and quantitative SIM using the 5'-ETS-1 RNA FISH probe plus mEGFP-RPA194 knock-in as an FC marker by Yao et al. 2019, revealed the emerging 47S pre-rRNA transit from the FC/DFC border to DFCs and the factors involved in much greater detail. Those data suggest "sorting into the subsequent compartments" (transit as they call) more convincingly. The data presented here support these findings well using a simpler experimental setup, without addressing the possible involvement of selected nucleolar proteins in this process through loss-of-function analyses.

5. Page 10 "Earlier reports suggested that the protein reorganization in both silencing of RNAPI-mediated transcription and DNA damage are similar (Franek et al, 2016), but we show that the spatial localization and levels of nascent rRNA can vary in case of DNA damage and transcriptional stress, perhaps related to the level of rRNA abrogation."
Franek et al., 2016 did not use BMH-21, but ActD. As originally described in Peltonen et al., 2014 PMID: 24434211 (should also be cited), BMH-21 causes different changes in nucleolar reorganization compared to ActD. It is not appropriate to cite the study by Franek et al., which uses a less specific RNA Pol I transcription inhibitor, without mentioning this.

6. While the control samples and the 4-NQO samples (1h treatment) in Fig. 4 show clear differences in the UBF and 5'-ETS signals, there is no obvious difference between these settings in Fig. 5, raising questions about the reproducibility of the

treatment effect or the robustness of the analytical method.

7. In the metric used to quantify stress-induced nucleolar reorganization using 5'-ETS RNA-FISH analyses, the Results section does not mention what the marker for the "nucleolar periphery/nucleolar perimeter" is (probably 28S, based on the Methods section, but this should at least be briefly mentioned in the Results section). More importantly, even in the Methods section, it remains unclear how the third dimension (Z-axis) and the unevenness of the "periphery/perimeter" are taken into account. Without clarification of the latter points, the numerical assessments and distance measurements remain ambiguous.

8. The use of transiently overexpressed GFP-UBF raises concerns and severely limits the explanatory power of the otherwise sound FRAP approach. First, the amount of overexpressed UBF strongly influences its localization. Second, the presence of endogenous UBF creates competition for rDNA binding. These problems can be solved most elegantly with an endogenous tagging approach, as used in the study by Yao et al. (2019). In addition, when using BMH-21, the induced degradation of RPA194, an interaction partner of UBF, should also be taken into account. Therefore, it remains unclear whether the effects on UBF localization and dynamics are due to the inhibition of rRNA synthesis, the loss of RPA194, or a combination of both, or something else. The causal relationships are highly speculative and are not sufficiently supported by the FRAP data. They could be discussed to some extent in the "Discussion" section.

9. The pH measurements using the SNARF-4F AM ester as a ratiometric pH sensor provide interesting results, but further investigation would be required to support well-founded conclusions. The cited study by Aryan et al. from 2023 reports very significant deacidification during Pol I inhibition using ActD and CX5461. BMH-21-dependent Pol I inhibition should be compared with ActD/CX5461-dependent Pol I inhibition (and a control measurement of the inhibitory effect should be performed). Orthogonal validation of the pH measurements (e.g., with a genetically encoded sensor with NoLS) would also be important due to the known limitations of SNARF-4F (potential cytotoxicity, low SNR).

Minor points

1. The placement of background information requires improvement: the Results section contains several paragraphs, some of which should be included in the Introduction and others in the Discussion section.

2. With regard to the work of van Sluis and McStay, not only their review article should be cited, but also the authors' primary work PMID: 26019174.

3. On Fig 3A the rDNA unit is not in scale.

4. For better understanding, the rRNA-making factories on page 15 should be termed actively transcribed rDNA units.

5. Page 11 "The cell nucleolus is emerging as a stress sensor within the cells(Yang et al., 2018). It has gained popularity as a potential biomarker for pathological conditions of cancer and neurodegeneration in the past decade."
The fact that enlarged and irregular nucleoli are characteristic features of cancer cells is known since the late 19th century and has been used by pathologist since many decades.

** As a service to authors, EMBO Press provides authors with the ability to transfer a manuscript that one journal cannot offer to publish to another journal, without the author having to upload the manuscript data again. To transfer your manuscript to another EMBO Press journal using this service, please click on Link Not Available

Review response plan – Ghosh & Mazumder

We thank the editor and the reviewers for their thoughtful feedback. Their insights will be invaluable in improving the clarity and rigor of this manuscript. We have addressed each comment below, with plans for revisions highlighted in **blue.**

Referee #1:

This manuscript uses expansion microscopy to resolve sub-nucleolar architecture and supports a model in which fibrillar center (FC) and dense fibrillar component (DFC) domains are organized as nested shells that reorganize under DNA damage or RNA polymerase I (RNAPI) inhibition. The authors further propose that stress-induced transcriptional downregulation remodels sub-nucleolar compartments from a viscoelastic state into more solid-like condensates, accompanied by perturbation of the nucleolar pH gradient due to loss of an rRNA scaffold. Based on imaging and quantitative spatial metrics, the authors claim that nucleolar reorganization arises primarily from reduced rRNA levels and consequent biophysical restructuring, rather than from active DNA damage signaling to facilitate rDNA repair. While the study is technically sound, the methodology has already been used to characterize nucleolar ultrastructure and stress-induced reorganization (PMID: 40391829). In the present work, the use of expansion microscopy is confined to protein imaging and does not include concurrent visualization of RNAs, which may limit the extent of new insights compared with previous reports. Moreover, the conclusion that nascent rRNA levels influence nucleolar reorganization under stress conditions has been explored in previous studies (PMID: 35978024). Finally, while the study presents extensive descriptive data, the mechanistic framework supporting several key conclusions remains incomplete. Additional experiments providing direct evidence would help to strengthen the causal interpretation of the results.

Major concerns:

1. The structural features of FC-DFC organization revealed by expansion microscopy (Figures 1-2), including nested shell-like arrangements, have largely been established previously using various super-resolution approaches (SIM, STED, and related methods). While the imaging quality is high, these observations alone do not substantially advance current understanding of nucleolar architecture. To strengthen the novelty, the authors should either provide additional new structural insights beyond what has already been reported, or temper their claims accordingly.

Author response: We agree that providing a clear distinction between our observations and the existing literature is essential. In accordance with the reviewer's suggestion, we will temper the language in the Abstract and Discussion, and add nuance where needed.

2. The manuscript treats 4-NQO (DNA damage) and BMH21 (RNAPI inhibition) as perturbations that converge on reduced rRNA synthesis. However, these are biologically distinct stresses that engage different signaling pathways and cellular responses. The current experimental design does not adequately disentangle effects of DNA damage from those of transcriptional shutdown. Without a cleaner experimental separation (e.g., transcriptional shutdown without DNA damage, or DNA damage that spares transcription), the interpretation remains ambiguous.

Author response: We agree with the Reviewer that DNA damage and transcriptional inhibition are often coupled, making it difficult to attribute nucleolar remodelling to a single cause. In fact, rRNA transcription downregulation under stress adds to the generality of the response, which in fact is a strength of the conclusions.

To further address this, the following experiments can help decouple the two effects.

> **Transcriptional Shutdown without DNA Damage:** We treated cells with **BMH21**, which inhibit RNA Polymerase I directly without inducing double-strand breaks. This allowed us to observe nucleolar reorganization driven purely by transcriptional arrest. We will add data to show that BMH21 does not trigger DNA damage response. We have verified this ourselves and will present the corresponding data.
>
> **DNA Damage and Transcriptional status:** We will use other DNA damaging agents, especially ones that spare transcription, to show the universality of the response. We will perform targeted laser micro-irradiation at specific sites to induce local DNA damage while monitoring nascent rRNA synthesis via **EU (5-ethynyl uridine) labelling**. This could confirm whether the structural changes to the nucleolus occur concomitant with downregulated transcription or not.

3. The FRAP experiments on GFP-UBF are appropriate to probe nucleolar dynamics, and the observed mild recovery changes after 4-NQO versus pronounced recovery loss after BMH21 are consistent with RNA-dependent modulation of condensate fluidity. However, FRAP alone is insufficient to conclusively demonstrate a transition from a viscoelastic to a solid-like state. Additional biophysical evidence would be required to support this claim.

Author response: We agree that FRAP data, while widely used and indicative of reduced molecular exchange, does not fully define the material state of the nucleolus. To provide more definitive biophysical evidence of a transition to a solid-like state, will perform **fluorescence anisotropy imaging of GFP-UBF** to probe the local order, viscosity and rotational constraints on the labelled proteins.

4. It may not be sufficiently rigorous to simply use 28S rRNA probes and 5' ETS probes as direct surrogates for mature and nascent rRNAs, respectively. 28S rRNA probes may also detect unprocessed rRNA precursors, indicating that the imaging signals represent a mixture of both mature and nascent rRNAs. In addition, 5' ETS is a part of SSU pre-rRNA, whereas 28S rRNA is a part of LSU pre-rRNA, reflecting two distinct rRNA maturation pathways. Consequently, changes in signal intensity upon stress treatment cannot be interpreted unambiguously as changes in the absolute abundance of nascent versus mature rRNAs. Instead, these differences may simply reflect altered processing kinetics between SSU and LSU pre-rRNAs. To strengthen the interpretation, it would be necessary to include probes targeting 18S rRNA as an additional control to more appropriately distinguish rRNA maturation states.

Author response: The 47S pre-rRNA transcript contains both the 5' ETS and the 28S sequences, but only the 28S is a part of a mature ribosome. Because both are parts of the same pre-RNA initially, we have used them as proxies for nascent and mature rRNA as done in previous studies (*Yao et. al 2019, Quinodoz et.al 2025*). We also agree with the reviewer that stress can differentially affect the maturation kinetics of the SSU (containing 18S) and the LSU (containing 28S). If needed we can attempt detection of 18S rRNA to compare the nascent and mature rRNA involved in the same processing pathway as suggested by the reviewer.

5. Both 4-NQO and BMH21 reduce rRNA expression level and induce nucleolar proteins shuttling, with BMH21 exerting even stronger effects. However, only the 4-NQO induces deacidification, the manuscript does not provide a mechanistic explanation for the observed differences in nucleolar pH between these two conditions. More broadly, this study would benefit from a clearer mechanistic explanation linking changes in biophysical properties to nucleolar organization and function. While the presented measurements provide valuable descriptive information, they are largely correlative, and additional mechanistic insight would be required to clarify how these biophysical parameters contribute to the regulation of nucleolar architecture.

Author response: We agree that providing a mechanistic framework linking pH, transcriptional activity, and biophysical states is essential for moving beyond a purely descriptive study. We will therefore investigate the changes in PAR (Poly-ADP-ribose) levels in reference to an earlier work (PMID: 37979585) under conditions of stress induced by 4-NQO and BMH21 to address the link between pH changes and deacidification of the organelle. We will also present data about the translocation of the DE tract proteins, which can contribute to deacidification. Together rRNA and PAR levels and translocation of the DE tract proteins will address this point comprehensively beyond just speculation.

Minor concerns:

1. In Figure 3D, the labeling of UBF and rRNA appears to be reversed, which does not match the description in the main text.

2. In Figure 4, panel D is referenced in the text, but no Figure 4D is present.

Author response: We apologize for these oversights, and will rectify the same.

Referee #2:

In this study, the authors utilize expansion microscopy to resolve changes in nucleolar architecture in response to DNA damage and transcription inhibition. They show that both DNA damage and inhibition of DNA synthesis alter nucleolar structure by reorganizing nucleolar layers and affecting transcriptional activity. In addition, the authors report that DNA damage or transcriptional inhibition is accompanied by an increase in nucleolar pH. Taken together, these results suggest that changes in nucleolar structure are largely driven by alterations in rRNA dynamics. Overall, the manuscript is well written, and the images are well presented and clearly described. While some of the observations reported here have been previously described in the field, from this reviewer's perspective the data are compelling and provide an orthogonal approach through which models and insights into nucleolar biology can be tested. As such, and regardless of some overlap with existing literature, I strongly support publication of this study in EMBO. However, while the findings are of interest, the overall scope appears more appropriate for a short report than a full article. Overall, this study clarifies and adds important new insights to the field. Although I cannot evaluate the technical details of the expansion microscopy itself, from a nucleolar and cell biology standpoint this study is conceptually significant and merits publication. That said, there are several concerns that should be addressed by the authors prior to publication.

Major Concerns:

1• Several statements throughout the manuscript are misleading or inaccurate. While I highlight specific examples below, the authors should exercise greater care in how they present arguments about the field. The fact that a possibility has been proposed in a prior study does not constitute proof. Clear distinctions must be made between what is hypothesized, what is shown, and what is experimentally demonstrated. The presence of a citation alone should not be treated as a proxy for established ground truth.

Author response: We take this feedback seriously and appreciate the Reviewer's call for greater precision in our scientific communication. We will review the manuscript and recalibrate our language to clearly distinguish between hypothesized models and proven facts.

2• the authors claim to perform experiments under conditions of DNA damage; however, the manuscript does not provide direct evidence that DNA damage is actually occurring. At a minimum, the authors should demonstrate induction of γH2AX foci or provide other markers of DNA damage response pathway activation to substantiate these claims.

Author response: We agree that direct validation of DNA damage is essential to support our claims regarding the nucleolar response to genotoxic stress. To substantiate that 4-NQO indeed induces DNA damage under our experimental conditions, we will add experiments which show γH2AX immunofluorescence in cells treated with 4-NQO to confirm the induction of DNA damage. In addition, we will explore other DNA damaging agents too and present that data.

3• The authors should perform a calibration curve for the pH measurements presented in Figure 6 or, alternatively, validate their findings using an independent chemical pH sensor. Such validation is necessary to strengthen confidence in the reported changes in nucleolar pH.

Author response: We apologize for this; we have performed the pH calibration but did not present that as previous studies have just reported relative changes. We will add the pH calibration curve to show our validation of the pH sensor.

Other Comments:

1• Please revised this misleading statement "However, the effects of cellular stress or disrupted transcription on nucleolar protein and RNA organization are still not well understood, limited both by the resolution of light microscopy, and lack of tools for detection of transcript species of interest and complex sample preparation in electron microscopy." Indeed, this an unresolved question. But the issue is resolution in general and not necessarily limited by resolution of light microscopy, the issue is more complex than that.

2• Please note that the following statement is inaccurate and misleading "The outflux of newly synthesized rRNA is facilitated by a combined action of K-block and D/E tracts enriched nucleolar proteins. The K-blocks create a scaffold of transient multivalent interactions that aid in nucleolar organization, while the D/E tracts serve as proton carriers that set up a pH gradient, both contributing to the directional export of processed rRNA." The outflux of rRNA is more complex than described and emerging studies suggest that K-block and D/E tracks containing-proteins contribute. However, this statement ignores the multitude of other process, molecules and biochemical reactions that contribute to this process overall. Therefore, this statement needs to be revised to reflect the facts and what is known and what is just a hypothesis. Similarly, it has been suggested that D/E tracks might serve as proton carriers, but this has not been formally proven and so it should not be state as ground truth.

3• This statement needs references "suggested that the rRNA transcription is initiated at the interface of FC-DFC with co-transcriptional processing, triggering phase separation and rRNA sorting into the subsequent compartments." This has been the prevailing view in the field for the last decade.

4• This statement is completely FALSE/Disproven "It is known that the rRNA leads to the nested tripartite structure of nucleoli (Farley et al, 2015; van Sluis & McStay, 2017) and direct or indirect inhibition of transcription leads to the nucleolar reorganization leading to its segregation or reoriented bipartite structure." The bipartite structure of the nucleolus is defined as the nucleoli structure in non-amniotes organism. It has absolutely nothing to do with the nucleolus under stress. Secondly, three reports have shown that the tripartite structure of the nucleolus is mediated by Treacle.

5• This statement is in principle inaccurate please revise "Condensates formed by RNA or protein or both, get seeded by upon increasing concentration and affinity towards each other"

6• This statement is inaccurate and misleading please revised "This is because rRNA can scaffold multivalent interaction among the Glycine-Arginine rich (GAR) resident proteins which, when altered, shifts to GAR-GAR interacting condensates making it more viscous or dense in nature"

Author response: We take this feedback seriously and appreciate the Reviewer's call for greater precision in our scientific communication. We do not mean to misrepresent the literature in the field. In our attempt to provide context, we may have inadvertently fallen short to convey the facts with precision and accuracy. We will comprehensively review the manuscript, rectify our mistakes and rightly redefine the existing premise, evidences and hypotheses.

Referee #3:

In their manuscript "Nucleolar reorganization on stress depends on physicochemical changes due to nascent rRNA synthesis" the authors use advanced fluorescence microscopy techniques (IF, RNA FISH, FRAP, fluorescence pH sensor in combination with expansion microscopy and SoRa super-resolution imaging) to observe changes in the nucleolus, particularly after treatment with 4-NQO and BMH-21. 4-NQO forms covalent adducts at purine bases, triggering a DNA damage response that indirectly leads to Pol I inhibition. BMH-21 acts as an intercalator of GC-rich DNA without a DNA damage response. Unlike 4-NQO and other Pol I inhibitors such as CX-5461, it causes rapid dissociation of Pol I from rDNA and subsequent proteasome-dependent degradation of the catalytic RPA194 subunit within 2 to 3 hours.

Although the topic is interesting, the claims often lack novelty and originality, and in some cases they are supported more convincingly by previously published results than by the authors' own findings (e.g. "Stress-induced transcription inhibition remodels the sub-nucleolar compartments from a viscoelastic state into solid-like condensates thereby perturbing the nucleolar pH gradient due to the missing rRNA scaffold" in the Abstract). The Title and Abstract promise a significant deepening of the mechanistic understanding of nucleolar reorganization under stress conditions. In fact, the general term "stress" mainly encompasses treatments with the two inhibitors (without systematically comparing them with other DDR-inducing or DDR-independent Pol I inhibitors). Monitoring of physicochemical changes is limited to pH measurements. The role of nascent RNA synthesis is extrapolated from FISH localization studies on pre-rRNA. Another important general criticism is that the context provided by the earlier literature is not adequately presented. Essential information available in the cited literature is not always correctly mentioned or is not detailed enough.

Given the complementary nature of the findings to previous studies (in particular these cited works: Yao et al, 2019, Mol Cell. Nascent Pre-rRNA Sorting via Phase Separation Drives the Assembly of Dense Fibrillar Components in the Human Nucleolus; Quinodoz et al, 2025, Nature. Mapping and engineering RNA-driven architecture of the multiphase nucleolus - the reference has to be properly formatted in the References; Alley et al, 2025, ACS Chem Biol.Expansion Microscopy Provides Nanoscale Insight into Nucleolar Reorganization and Nuclear Foci Formation during Nucleolar Stress) and the lack of in-depth analyses with orthogonal validations, the manuscript would be more suitable for a specialised cell biology journal than for EMBO Reports, which is why I cannot recommend it for publication.

Author response: We take cognizance of the reviewer's comments and we will attempt to restructure the manuscript to bring out the novelty of our results with respect to the recent studies.

Major points

1. The Introduction section should provide an appropriate introduction to various forms of nucleolar stress and clearly state the stress inducers used in the study (4-NQO and BMH-21) and their mechanism of action. Two recent review articles may be helpful here: Imamura R, Yasuhara T. Nucleolar Organization in Response to Transcriptional Stress. Cancer Sci 116, 2649-2656 (2025). Cerezo EL, Henry Y, Henras AK, Romeo Y. Ribosome biogenesis and homeostasis: in the front line to cope with cellular stress. Biochimie 238, 43-56 (2025). Furthermore, it must be clarified and emphasized that only the first steps of ribosome synthesis take place in the nucleolus and that pre-ribosomal particles undergo further maturation steps in the nucleoplasm and cytoplasm (see Vanden Broeck and Klinge 2024 from the cited literature). Also the statement on page 8 "The modified RNA then diffuses to GC where they assemble with the ribosomal subunits to form mature ribosomal subunits (Dogra & Kriwacki, 2025)." has to be corrected. The multistep process of ribosome assembly is not completed in the GC.

Author response: We thank the Reviewer for these specific suggestions to improve the accuracy and depth of our Introduction and Results. We agree that a more nuanced discussion of nucleolar stress and the spatial constraints of ribosome maturation is necessary. We will expand

the Introduction on details of nucleolar Stress and provide a clearer rationale for our choice of stress inducers and their function. We will further clarify on the ribosome biogenesis stages and assembly site. We will also investigate the effects of other DNA damage inducers to provide more evidence to substantiate our claims.

2. The authors state on pages 4 and 7: "But how the rRNA outward flux is spatially altered within the nucleolar sub-compartments in healthy versus stressed cells is still elusive,..." "Although it is known that transcriptional inhibition and DNA damage leads to nucleolar reorganization(Boulon et al., 2010; Shav-Tal et al, 2005; Thomas Haaf, (1996); van Sluis & McStay, 2017), how the rRNA synthesis and maturation impact nucleolar phases and gate the outward rRNA flux is poorly understood." I cannot agree with this, as the publication 'Mapping and engineering RNA-driven architecture of the multiphase nucleolus' by Quinodoz et al. 2025 in Nature revealed a considerable amount of detail using complementary experimental approaches (SRM + NGS) at a very high technical level. They used chemical inhibition of rRNA processing (FVP) and, above all, a series of genetic inhibitions that provide clearer insights into the factors and mechanisms involved.

Author response: We appreciate the Reviewer's concern regarding the distinction between published work and our data. We specifically wanted to highlight that the correlation between spatially altered rRNA outflux in the context of damage is less studied. But we will review the language and argument to make the narrative more technically sound.

3. The authors write on page 6: "A very recent study has indeed reported the use to expansion microscopy to study nucleolar structures (Alley et al., 2025), but beyond just reporting the structural details under normal homeostasis, we further investigated how it is reorganized under stress and the roles of rRNA in such reorganization" The results related to signal intensity and localization studies of rRNA using RNA-FISH of the 5'-ETS and 28S probes do not explain the role of rRNA in stress-dependent reorganization of the nucleolus. Although they may be related, this also applies to numerous other nucleolar factors that were not investigated in the experiments and could be causal. A particularly interesting candidate would be RPA194, which is rapidly degraded when treated with BMH-21, but not when treated with other Pol I inhibitors. It should also be emphasized that Alley 2025 also applied stress.: "We investigate time-dependent changes in nucleolar structure and function under nucleolar stress induced by oxaliplatin, actinomycin D, and other platinum-based compounds."

Author response: We thank the Reviewer for this critical perspective on the causality of nucleolar reorganization. We agree that while rRNA depletion is a hallmark of the observed structural changes, it is essential to consider the role of the transcriptional machinery itself, specifically the RNA Polymerase I complex. Therefore, we will investigate the roles of nucleolar factors like RPA194 to provide a well-rounded approach to substantiate our claims. Though even an effect on RPA194 and hence RNA Pol I will result ultimately in reduced rRNA levels.

We will also revise our manuscript to properly credit Alley 2025 and discuss how our approach complements their findings. What we meant was that there was no detection of specific rRNA species relating to stress induced nucleolar reorganization in that study. We apologize that this did not come across and will modify our manuscript to reflect this.

4. On page 9, part of the text is very vague and partly incomprehensible: "UBF1 is in itself a 3D-shell like structure" probably refers to the 3D structure of individual active rDNA units bound with UBF, rather than the structure of UBF1 itself. The meaning of this sentence is unclear: "Co-stained FC-DFC modules (using UBF1 and FIB) along with 5'ETS to understand the spatial localization of the nascent pre-rRNA showed that the pre-rRNA partially overlapped with FIB seen in the images as well as the line profile (Figure 3E, F)." The causal relationship between the two parts of the next sentence is not substantiated by the data provided: "The partial interspersion of 5'ETS with FIB signals (Figure 3F) suggested that the rRNA transcription is initiated at the interface of FC-DFC with co-transcriptional processing, triggering phase separation and rRNA sorting into the subsequent compartments." Thorough analyses using shRNA-mediated depletion of 17 factors and quantitative SIM using the 5'-ETS-1 RNA FISH probe plus mEGFP-RPA194 knock-in as an FC marker by Yao et al. 2019, revealed the emerging 47S pre-rRNA transit from the FC/DFC border to DFCs and the factors involved in much greater detail. Those data suggest "sorting into the subsequent compartments" (transit as they call) more convincingly. The data presented here support these findings well using a simpler experimental setup, without addressing the possible involvement of selected nucleolar proteins in this process through loss-of-function analyses.

Author response: We thank the Reviewer for their feedback on the language. In our attempt to provide context, we may have inadvertently fallen short to convey the message with precision. We will ensure that the roles of other nucleolar proteins are also discussed. We will comprehensively review the manuscript, and refine our causal claims with evidence and references.

5. Page 10 "Earlier reports suggested that the protein reorganization in both silencing of RNAPI-mediated transcription and DNA damage are similar (Franek et al, 2016), but we show that the spatial localization and levels of nascent rRNA can vary in case of DNA damage and transcriptional stress, perhaps related to the level of rRNA abrogation." Franek et al., 2016 did not use BMH-21, but ActD. As originally described in Peltonen et al., 2014 PMID: 24434211 (should also be cited), BMH-21 causes different changes in nucleolar reorganization compared to ActD. It is not appropriate to cite the study by Franek et al., which uses a less specific RNA Pol I transcription inhibitor, without mentioning this.

Author response: We appreciate the Reviewer's correction regarding the citation of **Franek et al. (2016)**. We acknowledge that the nucleolar reorganization induced by Actinomycin D (ActD) and BMH21 is mechanistically and morphologically distinct due to their different modes of action. We will revise the manuscript to clarify that Franek et al. utilized ActD. We will provide citations to explicitly distinguish between the DNA-intercalating effects of ActD and the proteasome-mediated degradation of the RPA190 subunit induced by BMH21.

6. While the control samples and the 4-NQO samples (1h treatment) in Fig. 4 show clear differences in the UBF and 5'-ETS signals, there is no obvious difference between these settings in Fig. 5, raising questions about the reproducibility of the treatment effect or the robustness of the analytical method.

Author response: We take the Reviewer's concerns seriously will revise the manuscript to rectify for any perceived discrepancies between the images and quantification that can arise because of the specific image chosen.

7. In the metric used to quantify stress-induced nucleolar reorganization using 5'-ETS RNA-FISH analyses, the Results section does not mention what the marker for the "nucleolar periphery/nucleolar perimeter" is (probably 28S, based on the Methods section, but this should at least be briefly mentioned in the Results section). More importantly, even in the Methods section, it remains unclear how the third dimension (Z-axis) and the unevenness of the "periphery/perimeter" are taken into account. Without clarification of the latter points, the numerical assessments and distance measurements remain ambiguous.

Author response: We acknowledge the Reviewer's concern regarding the detailing of the methods. We will revise the manuscript to provide the analysis greater details and clarity.

8. The use of transiently overexpressed GFP-UBF raises concerns and severely limits the explanatory power of the otherwise sound FRAP approach. First, the amount of overexpressed UBF strongly influences its localization. Second, the presence of endogenous UBF creates competition for rDNA binding. These problems can be solved most elegantly with an endogenous tagging approach, as used in the study by Yao et al. (2019). In addition, when using BMH-21, the induced degradation of RPA194, an interaction partner of UBF, should also be taken into account. Therefore, it remains unclear whether the effects on UBF localization and dynamics are due to the inhibition of rRNA synthesis, the loss of RPA194, or a combination of both, or something else. The causal relationships are highly speculative and are not sufficiently supported by the FRAP data. They could be discussed to some extent in the "Discussion" section.

Author response: We agree that FRAP data of GFP-UBF, while indicative of reduced molecular exchange, alone does not fully define the material state of the nucleolus. We agree that endogenous tagging (as demonstrated by **Yao et al. 2019**) is the gold standard for avoiding artifacts related to protein stoichiometry and competition for rDNA binding sites. However, we are comparing among similarly transfected cells, so our results still hold. Cells with similar expression were chosen and we will show this explicitly by further analysing our FRAP data. We agree that the degradation of RPA194 by BMH21 complicates the interpretation. If UBF's scaffold (the Pol I complex) is degraded, its dynamics will inevitably change, but should in fact become faster. We will update the manuscript to add that the data may be a combined effect of both loss of nascent rRNA and the physical dissolution of the Pol I-RPA194 scaffold. Further to provide more definitive biophysical evidence of a transition to a solid-like state, we plan to perform new experiments of fluorescence anisotropy imaging to investigate local order.

9. The pH measurements using the SNARF-4F AM ester as a ratiometric pH sensor provide interesting results, but further investigation would be required to support well-founded conclusions. The cited study by Aryan et al. from 2023 reports very significant deacidification during Pol I inhibition using ActD and CX5461. BMH-21-dependent Pol I inhibition should be compared with ActD/CX5461-dependent Pol I inhibition (and a control measurement of the inhibitory effect should be performed). Orthogonal validation of the pH measurements (e.g.,

with a genetically encoded sensor with NoLS) would also be important due to the known limitations of SNARF-4F (potential cytotoxicity, low SNR).

Author response: We thank the Reviewer for this rigorous assessment of our pH measurements. The distinction between different Pol I inhibitors is critical, and will therefore validating the SNARF-4F results with established benchmarks and orthogonal methods is necessary for a well-founded conclusion. Previous studies have reported relative changes, but SNARF fluorescence ratios can be also calibrated to absolute pH values. We have ourselves verified this, and will present that data in the revised manuscript.

Minor points

1. The placement of background information requires improvement: the Results section contains several paragraphs, some of which should be included in the Introduction and others in the Discussion section.

2. With regard to the work of van Sluis and McStay, not only their review article should be cited, but also the authors' primary work PMID: 26019174.

3. On Fig 3A the rDNA unit is not in scale.

4. For better understanding, the rRNA-making factories on page 15 should be termed actively transcribed rDNA units.

5. Page 11 "The cell nucleolus is emerging as a stress sensor within the cells (Yang et al., 2018). It has gained popularity as a potential biomarker for pathological conditions of cancer and neurodegeneration in the past decade." The fact that enlarged and irregular nucleoli are characteristic features of cancer cells is known since the late 19th century and has been used by pathologist since many decades.

Author response: We will address these issues comprehensively in the addressed manuscript.

Dear Aprotim,

Thank you for your email asking us to reconsider our decision on your ms, and for your proposed revision plan. I have read through your plan now and also shared it with referee 1, who agrees that a suitably revised ms could be a good fit for EMBO reports.

I would thus like to invite you to revise your ms along the lines you suggest.
Please note that the referee concerns must be fully addressed and their suggestions taken on board, and that we cannot predict the outcome of the second round of review. Please address all referee concerns in a complete point-by-point response.

Acceptance of the manuscript will depend on a positive outcome of a second round of review. It is EMBO reports policy to allow a single round of major revision only and acceptance or rejection of the manuscript will therefore depend on the completeness of your responses included in the next, final version of the manuscript.

We realize that it is difficult to revise to a specific deadline. In the interest of protecting the conceptual advance provided by the work, we recommend a revision within 3 months (10th Jun 2026). Please discuss the revision progress ahead of this time with the editor if you require more time to complete the revisions.

IMPORTANT NOTE: we perform an initial quality control of all revised manuscripts before re-review. Your manuscript will FAIL this control and the handling will be DELAYED if the following APPLIES:
1) A data availability section providing access to data deposited in public databases is missing. If you have not deposited any data, please add a sentence to the data availability section that explains that.
2) Your manuscript contains statistics and error bars based on n=2. Please use scatter blots in these cases. No statistics should be calculated if n=2.

When submitting your revised manuscript, please review the submission guidelines (https://link.springer.com/journal/44319/submission-guidelines#cms-Revised-submissions) and carefully review the instructions that follow below. Failure to include requested items will delay the evaluation of your revision.

1) a .docx formatted version of the manuscript text (including legends for main figures, EV figures and tables). Please make sure that the changes are highlighted to be clearly visible.

2) individual production quality figure files as .eps, .tif, .jpg (one file per figure). See hhttps://media.springernature.com/original/springer-cms/rest/v1/content/27825798/data/v1 for more info on how to prepare your figures.

3) We replaced Supplementary Information with Expanded View (EV) Figures and Tables that are collapsible/expandable online. A maximum of 5 EV Figures can be typeset. EV Figures should be cited as 'Figure EV1, Figure EV2" etc... in the text and their respective legends should be included in the main text after the legends of regular figures.

- For the figures that you do NOT wish to display as Expanded View figures, they should be bundled together with their legends in a single PDF file called *Appendix*, which should start with a short Table of Content. Appendix figures should be referred to in the main text as: "Appendix Figure S1, Appendix Figure S2" etc. See 'Expanded View' section of the submission guidelines.

- Additional Tables/Datasets should be labeled and referred to as Table EV1, Dataset EV1, etc. Legends have to be provided in a separate tab in case of .xls files. Alternatively, the legend can be supplied as a separate text file (README) and zipped together with the Table/Dataset file.

4) a .docx formatted letter INCLUDING the reviewers' reports and your detailed point-by-point responses to their comments. As part of the EMBO Press transparent editorial process, the point-by-point response is part of the Review Process File (RPF), which will be published alongside your paper.

5) a complete author checklist, which you can download from our author guidelines. Please insert information in the checklist that is also reflected in the manuscript. The completed author checklist will also be part of the RPF.

6) Please note that all corresponding authors are required to supply an ORCID ID for their name upon submission of a revised manuscript (<https://orcid.org/>), which can be linked to your profile in the manuscript submission system.

7) Before submitting your revision, primary datasets produced in this study need to be deposited in an appropriate public database. Please remember to provide a reviewer password if the datasets are not yet public. The accession numbers and database should be listed in a formal "Data Availability" section placed after Methods. Please note that the Data Availability Section is restricted to new primary data that are part of this study. * Note - All links should resolve to a page where the data can

be accessed. *

If your study has not produced novel datasets, please mention this fact in the Data Availability Section.

8) At EMBO Press we ask authors to provide source data for the main manuscript figures. You will receive a separate email with instructions for providing source data with your revised manuscript, including how to upload and organize the files.

9) Our journal also encourages inclusion of *data citations in the reference list* to directly cite datasets that were re-used and obtained from public databases. Data citations in the article text are distinct from normal bibliographical citations and should directly link to the database records from which the data can be accessed. In the main text, data citations are formatted as follows: "Data ref: Smith et al, 2001" or "Data ref: NCBI Sequence Read Archive PRJNA342805, 2017". In the Reference list, data citations must be labeled with "[DATASET]". A data reference must provide the database name, accession number/identifiers and a resolvable link to the landing page from which the data can be accessed at the end of the reference.

10) Regarding data quantification, the following points must be specified in each figure legend:

- the name of the statistical test used to generate error bars and P values,

- the number (n) of independent experiments (please specify technical or biological replicates) underlying each data point,

- the nature of the bars and error bars (s.d., s.e.m.),

- If the data are obtained from n Program fragment delivered error ``Can't locate object method "less" via package "than" (perhaps you forgot to load "than"?) at //ejpvfs23/sites23b/embor_www/letters/embor_decision_revise_and_review.txt line 54.' 2, use scatter blots showing the individual data points.

Discussion of statistical methodology can be reported in the materials and methods section, but figure legends should contain a basic description of n, P and the test applied.

- Please also include scale bars in all microscopy images.

11) The journal requires a statement specifying whether or not authors have competing interests (defined as all potential or actual interests that could be perceived to influence the presentation or interpretation of an article). In case of competing interests, this must be specified in your disclosure statement.

12) All Materials and Methods need to be described in the main text using our 'Structured Methods' format, which is required for all research articles. According to this format, the Methods section includes a separate Reagents and Tools Table file (listing key reagents, experimental models, software and relevant equipment and including their sources and relevant identifiers) and a Methods and Protocols section describing the methods using a step-by-step protocol format. The aim is to facilitate adoption of the methodologies across labs. More information on how to adhere to this format as well as a downloadable template (.docx) for the Reagents and Tools Table can be found in our author guidelines.

We would also welcome the submission of cover suggestions, or motifs to be used by our Graphics Illustrator in designing a cover.

As part of the EMBO publication's Transparent Editorial Process, EMBO reports publishes online a Review Process File (RPF) to accompany accepted manuscripts. This File will be published in conjunction with your paper and will include the referee reports, your point-by-point response and all pertinent correspondence relating to the manuscript.

You are able to opt out of this by letting the editorial office know (emboreports@embo.org). If you do opt out, the Review Process File link will point to the following statement: "No Review Process File is available with this article, as the authors have chosen not to make the review process public in this case."

I look forward to seeing a revised form of your manuscript when it is ready.

Yours sincerely,

Esther Schnapp, PhD
Senior Editor
EMBO reports

Response to Reviewers' Comments

We thank the editor and the reviewers for their thoughtful feedback. Their insights have been invaluable in improving the clarity and rigor of this manuscript. We have addressed each comment below, with author responses highlighted in **blue.**

Referee#1:

This manuscript uses expansion microscopy to resolve sub-nucleolar architecture and supports a model in which fibrillar center (FC) and dense fibrillar component (DFC) domains are organized as nested shells that reorganize under DNA damage or RNA polymerase I (RNAPI) inhibition. The authors further propose that stress-induced transcriptional downregulation remodels sub-nucleolar compartments from a viscoelastic state into more solid-like condensates, accompanied by perturbation of the nucleolar pH gradient due to loss of an rRNA scaffold. Based on imaging and quantitative spatial metrics, the authors claim that nucleolar reorganization arises primarily from reduced rRNA levels and consequent biophysical restructuring, rather than from active DNA damage signaling to facilitate rDNA repair. While the study is technically sound, the methodology has already been used to characterize nucleolar ultrastructure and stress-induced reorganization (PMID: 40391829). In the present work, the use of expansion microscopy is confined to protein imaging and does not include concurrent visualization of RNAs, which may limit the extent of new insights compared with previous reports. Moreover, the conclusion that nascent rRNA levels influence nucleolar reorganization under stress conditions has been explored in previous studies (PMID: 35978024). Finally, while the study presents extensive descriptive data, the mechanistic framework supporting several key conclusions remains incomplete. Additional experiments providing direct evidence would help to strengthen the causal interpretation of the results.

Author response: We thank the reviewer for their positive comments on our manuscript and address the specific points they have raised, below.

Major concerns:

1. The structural features of FC-DFC organization revealed by expansion microscopy (Figures 1-2), including nested shell-like arrangements, have largely been established previously using various super-resolution approaches (SIM, STED, and related methods). While the imaging quality is high, these observations alone do not substantially advance current understanding of nucleolar architecture. To strengthen the novelty, the authors should either provide additional new structural insights beyond what has already been reported, or temper their claims accordingly.

Author response: We agree that providing a clear distinction between our observations and the existing literature is essential. In accordance with the reviewer's suggestion, we have tempered the language in the manuscript and added nuance where needed. New experiments have been performed to address reviewers' comments and lend additional credence to the claims made.

2. The manuscript treats 4-NQO (DNA damage) and BMH21 (RNAPI inhibition) as perturbations that converge on reduced rRNA synthesis. However, these are biologically distinct stresses that engage different signalling pathways and cellular responses. The current experimental design does not adequately disentangle effects of DNA damage from those of

transcriptional shutdown. Without a cleaner experimental separation (e.g., transcriptional shutdown without DNA damage, or DNA damage that spares transcription), the interpretation remains ambiguous.

Author response: We agree with the reviewer that DNA damage and transcriptional inhibition are often coupled, making it difficult to attribute nucleolar remodelling to a single cause. In fact, rRNA transcription downregulation under conditions of DNA damage or stress generally is well-documented(James *et al*, 2014; Kruhlak *et al*, 2006; Shav-Tal *et al*, 2005). This, to our mind, is a general feature and adds to the strength of the conclusions drawn. It is difficult to cause DNA damage while guaranteeing no effect on rRNA. But the converse experiment can be done. RNA Pol I inhibitors like Actinomycin D also cause DNA damage and replication stress like responses (Ma & Pederson, 2013; Yin *et al*, 2019). But an inhibitor like BMH21 used in our studies is thought not to. To address this, we treated cells with **BMH21**, which inhibit RNA Polymerase I directly without inducing DNA damage. We treated cells with 4-NQO (1µg/ml, 1hr) and BMH21 (2µM,3hrs), and stained them with antibodies against γH2AX and RNAP1. γH2AX is induced by DNA double strand breaks and also by UV, DNA alkylating agents etc. (Ma & Pederson, 2013; Yin *et al.*, 2019) and is used here as a general proxy for a DNA damage response (DDR). This allowed us to observe nucleolar reorganization driven majorly by transcriptional arrest. The data below show that, compared to 4-NQO, BMH21 does not induce γH2AX as seen in the immunofluorescence assay (Figure R1). Thus, nucleolar reorganization may be a general outcome of downregulated rRNA transcription (which also occurs under conditions of DNA damage) as discussed in our study. We have now added this data to the manuscript as Supplementary Figure EV3.

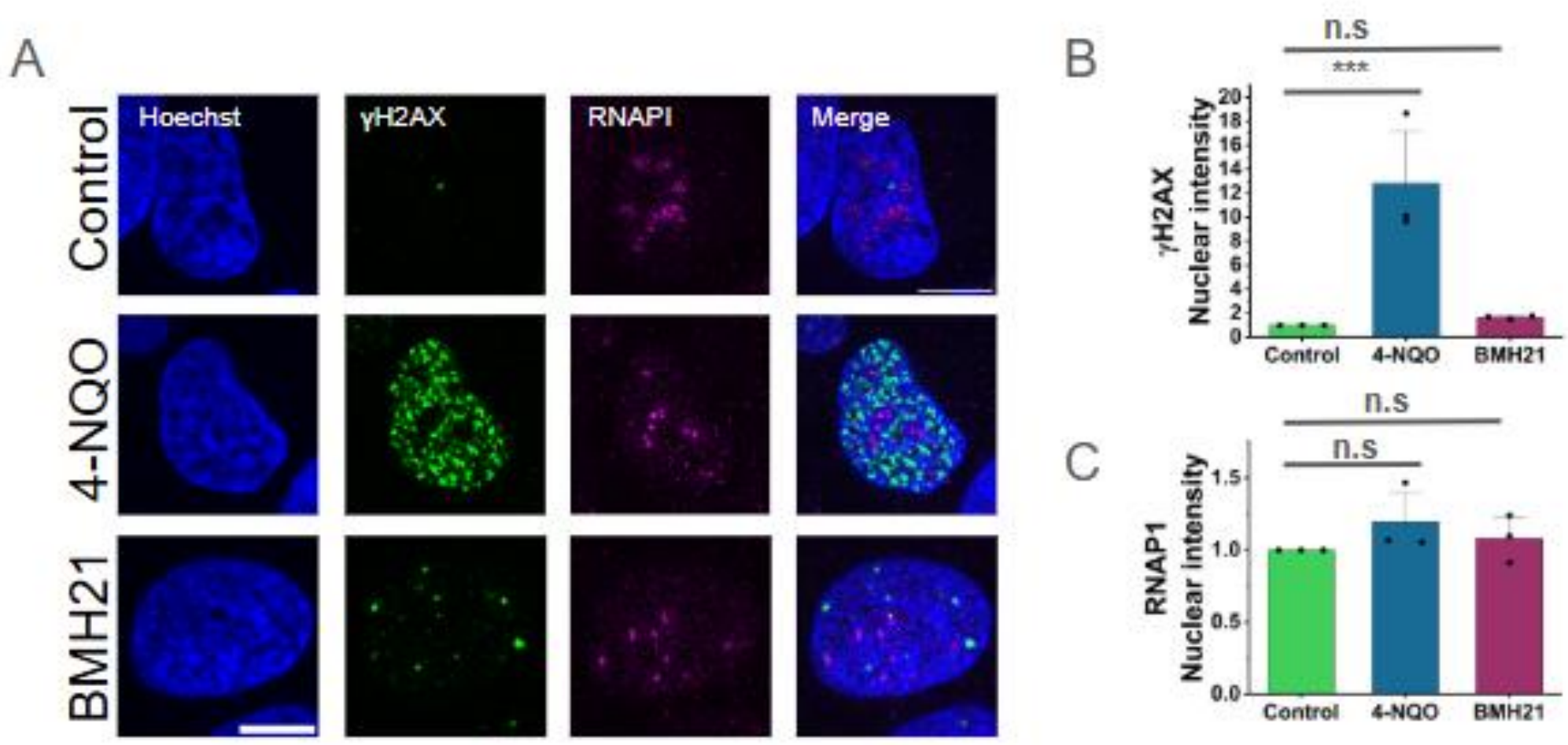

*Figure R1: U2OS cell nuclei from cells treated with 1ug/ml 4NQO for 1hr or 2uM of BMH21 for 3hrs, compared to control nuclei, (A) stained with Hoechst (blue), γH2AX (green) and RNAPI (magenta) (B) Mean nuclear intensity of γH2AX and (C) RNAPI in U2OS cell normalized to the mean of the corresponding control groups. Data represents mean ± S.E.M, n= 60,44,47 nuclei over three independent experiments. Statistical significance calculated using two sample t-test. p<0.0001 is indicated by ***, other comparisons are not significant at p< 0.01 (n.s). Scale bar is 10µm.*

1.The FRAP experiments on GFP-UBF are appropriate to probe nucleolar dynamics, and the observed mild recovery changes after 4-NQO versus pronounced recovery loss after BMH21 are consistent with RNA-dependent modulation of condensate fluidity. However, FRAP alone

is insufficient to conclusively demonstrate a transition from a viscoelastic to a solid-like state. Additional biophysical evidence would be required to support this claim.

Author response: Markedly reduced mobility in FRAP data is widely used as a proxy for transition to solid-like state (Bose *et al*, 2022; Lin *et al*, 2015; Patel *et al*, 2015). However, we agree that FRAP data, while widely used and indicative of reduced molecular exchange and mobility, does not fully define the material state of the nucleolus. It is a reflection of translational mobility of labelled molecules which can go down in solid-like state but can go down with increased viscosity too. To provide further biophysical evidence of a transition to a solid-like state, we performed **fluorescence anisotropy imaging of GFP-UBF** to probe the local order, viscosity and rotational constraints on the labelled proteins. Fluorescence Anisotropy reports on rotational diffusion of molecules and can report on local ordering (Kesavan *et al*, 2020; Lakowicz, 2006; Saha *et al*, 2016). If transition to a solid-like state occurs perhaps we could see values of anisotropy greater than what would be expected from a random orientation of fluorophores. At the very least if anisotropy increases under any condition, it can indicate reduced rotational mobility (Kesavan *et al.*, 2020; Lakowicz, 2006). We observed that cells treated with BMH21 has significantly higher anisotropy values than control cells (Fig. R2), while cells treated with 4-NQO show similar anisotropy values to control cells. This is pretty much in line with the FRAP data. While highly ordered states of GFP-UBF cannot be claimed, this is indeed indicative of slower rotational mobility. BMH21 leads to disassembly of the RNAPI machinery, leading to trapped UBF on the rDNA which is immobile and potentially solid-like. We have now added this data to the manuscript as EV5 and also moderated the claims about a solid-like state.

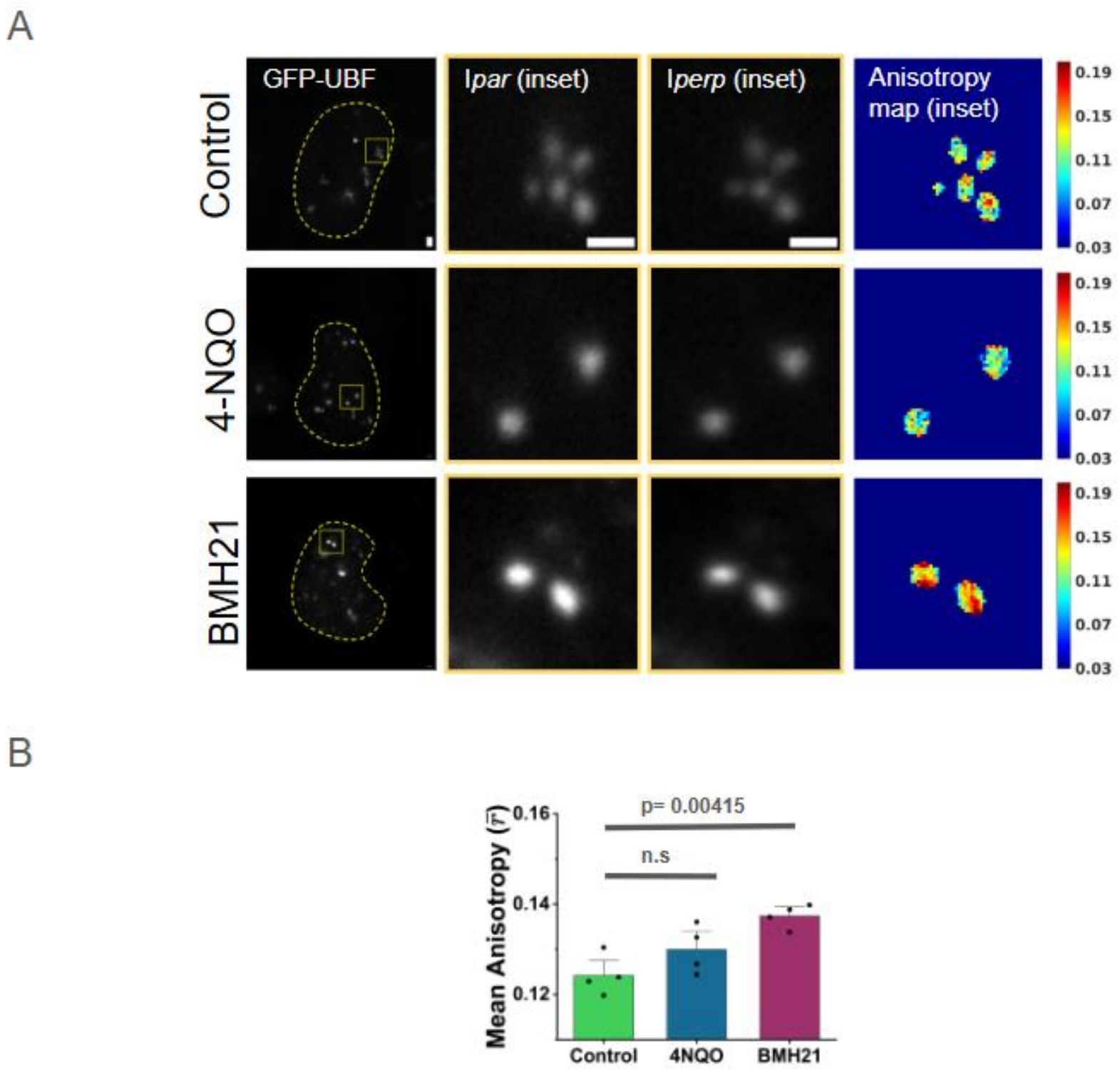

Figure R2: (A) U2OS cells were transfected with GFP-UBF. Representative nuclei from cells treated with 1ug/ml 4NQO for 1hr or 2uM of BMH21 for 3hrs, compared to control nuclei are shown on the leftmost column. The

nuclear boundaries are shown by the dotted line, and zoomed in images of the nucleoli in the inset boxes are shown on the right. The parallel (Ipar) and perpendicular (Iperp) components of the emission light are collected and used to compute the anisotropy maps. Anisotropy maps of the representative nucleoli are shown on the rightmost column. (B) Average of the mean anisotropy values of nucleolar GFP-UBF foci. Data represents mean ± S.E.M, n>=200 (225; 217;230) nucleoli over four independent experiments. Statistical significance calculated using Two-sample t-test. P value (p=0.00415) indicates that the comparison is significant at p<0.005, while the other comparison is non-significant (n.s) at p<0.005. Scale bar is 10µm.

4. It may not be sufficiently rigorous to simply use 28S rRNA probes and 5' ETS probes as direct surrogates for mature and nascent rRNAs, respectively. 28S rRNA probes may also detect unprocessed rRNA precursors, indicating that the imaging signals represent a mixture of both mature and nascent rRNAs. In addition, 5' ETS is a part of SSU pre-rRNA, whereas 28S rRNA is a part of LSU pre-rRNA, reflecting two distinct rRNA maturation pathways. Consequently, changes in signal intensity upon stress treatment cannot be interpreted unambiguously as changes in the absolute abundance of nascent versus mature rRNAs. Instead, these differences may simply reflect altered processing kinetics between SSU and LSU pre-rRNAs. To strengthen the interpretation, it would be necessary to include probes targeting 18S rRNA as an additional control to more appropriately distinguish rRNA maturation states.

Author response: The 47S pre-rRNA transcript contains both the 5' ETS and the 28S sequences, but only the 28S is a part of a mature ribosome. Because both are parts of the same pre-RNA initially, we have used them as proxies for nascent and mature rRNA as done in previous studies (*Yao et. al 2019, Quinodoz et.al 2025*). However, to address the point brought up by the reviewer that stress can differentially affect the maturation kinetics of the SSU (containing 18S) and the LSU (containing 28S), we performed the same experiments with probes now designed to detect the 18S rRNA. We simultaneously visualized 18S rRNA and 5'ETS in the same cells to compare the nascent and mature rRNA involved in the same processing pathway.

Somewhat strikingly we find that both 18S and its corresponding upstream spacer, 5'ETS show progressive decline across treatments of 4-NQO and BMH21 indicating that the small subunit pathway is sensitive to transcriptional alterations, undergoing rapid quality-controlled degradation when transcription is perturbed. Upon stress, ribosomal subunits show differential clearance where 18S containing intermediates are more sensitive to damage, therefore get rapidly degraded via nuclear exosome or non-functional rRNA decay(Coria *et al*, 2025; Ferreira *et al*, 2020). 28S remains partially stable, still retaining fragments within the nucleoli, that take longer to degrade. However, despite the change in 18S levels the relative fold change for the mature 18S is much smaller (0.54 for 4-NQO and 0.34 for BMH21) than for the nascent 5' ETS (0.34 for 4-NQO, 0.1 for BMH21, Figure R3 in this file). Mature 28S rRNA possesses the intrinsic multivalent properties required to organize the multilayered tripartite structure (FC-DFC-GC) during development(Wei *et al*, 2024). But, mature (18S or 28S) or partially processed RNA fragments in itself is insufficient for in vivo homeostatic maintenance of this organization, and cannot substitute for the electrostatic scaffolding provided by actively transcribed nascent rRNA to maintain the liquid like dynamics of these sub-compartments. We thank the reviewer for suggesting this experiment. Because the point about more modest effects on mature rRNA compared to nascent still remains, and as all originate from the same 47S precursor, we have left the 28S data in the main figure and included the 18S data as a Supplementary Figure EV4. We have now discussed this point in the manuscript.

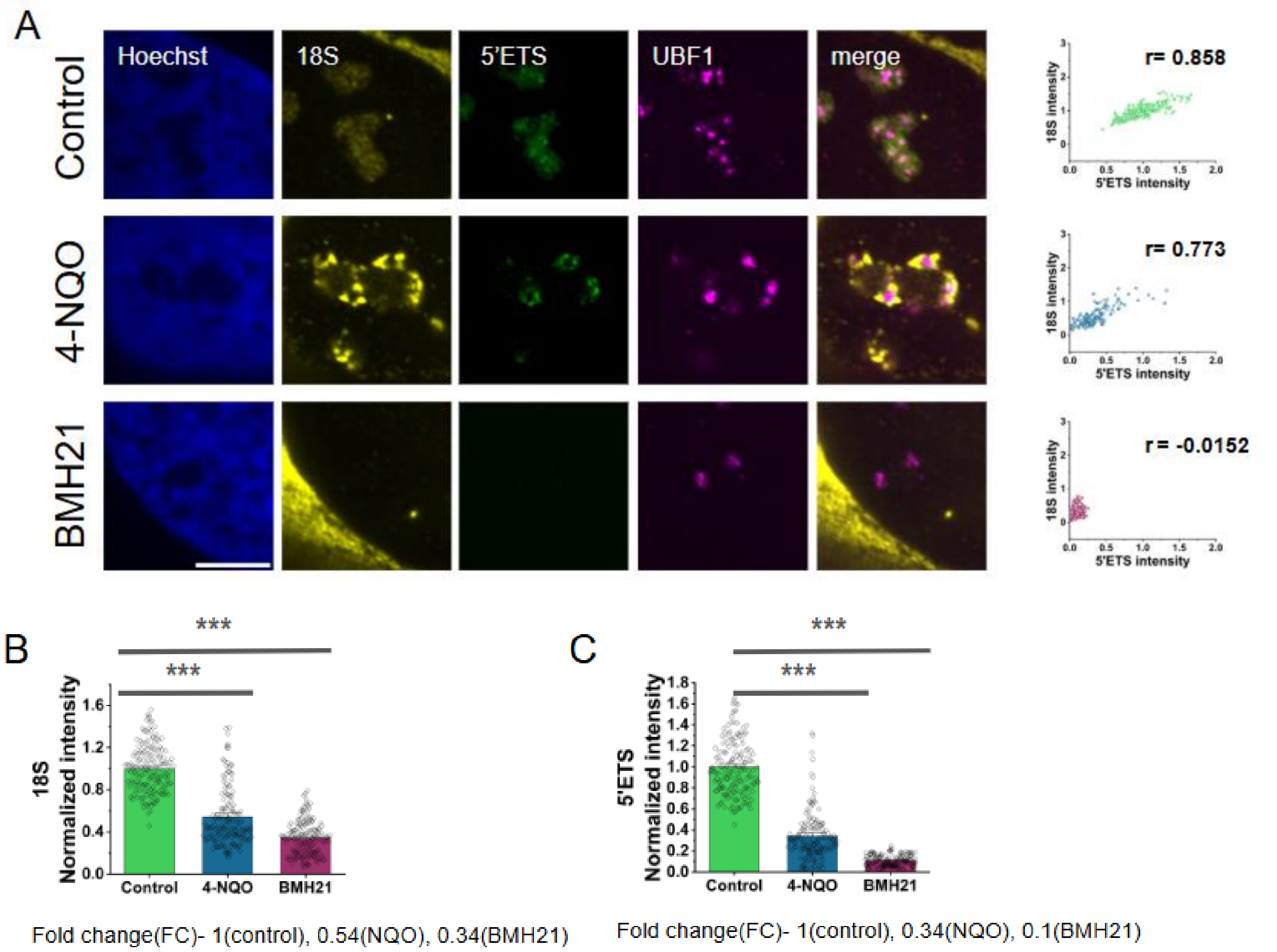

*Figure R3: U2OS cell nuclei from cells treated with 1ug/ml 4NQO for 1hr or 2uM of BMH21 for 3hrs, compared to control nuclei, (A) representative cell nucleoli from cells stained with Hoechst (blue), probed for 18S rRNA (yellow), 5'ETS rRNA (green), UBF1 (magenta), and merge are shown. Scatter plots for correlation between 18S and 5'ETS, showing the normalized intensity for each rRNA species are shown on the right. The Pearson's correlation coefficient (r) is mentioned on the graphs. The correlation is significant for control and 4-NQO and non-significant for BMH21at p< 0.0001. (B) Normalized intensity of 18S rRNA and (C) Normalized intensity of 5'ETS rRNA. Data represents mean ± S.E.M, with the mean labelled for each condition. n > = 150 (165;150;158) nucleoli over three independent experiments. Statistical significance was calculated using a K.S. test for single cells distributions, where *** represents data significant at p<0.0001. Fold changes (FC) with respect to control is 0.54 for 4-NQO and 0.34 for BMH21 in (B), while it is 0.34 for 4-NQO and 0.1 for BMH21 in (C). While raw intensities in arbitrary units were plotted in Figure 4, here in A-C, intensities normalized to the mean of controls in each of the three experiments have been plotted to infer relative fold changes.*

5. Both 4-NQO and BMH21 reduce rRNA expression level and induce nucleolar proteins shuttling, with BMH21 exerting even stronger effects. However, only the 4-NQO induces deacidification, the manuscript does not provide a mechanistic explanation for the observed differences in nucleolar pH between these two conditions. More broadly, this study would benefit from a clearer mechanistic explanation linking changes in biophysical properties to nucleolar organization and function. While the presented measurements provide valuable descriptive information, they are largely correlative, and additional mechanistic insight would be required to clarify how these biophysical parameters contribute to the regulation of nucleolar architecture.

Author response: We agree that providing a mechanistic framework linking pH, transcriptional activity, and biophysical states is essential for moving beyond a purely descriptive study. We have shown loss of nascent rRNA in our study – this can be a significant source of deacidification. But also as discussed, other sources of deacidification also exists; one could be PAR (Poly-ADP-ribose) loss as PARP1 translocates from the nucleolus under conditions of stress(Rancourt & Satoh, 2009); yet another could be the translocation of DE tract proteins like NPM, NCL and Ki-67 occupying the GC region and the nucleolar rim, which can contribute to deacidification.

To specifically see if these are indeed reasonable speculations, we performed experiments where we detect PARP1 with a chromobody that detects endogenous PARP1 in live cells (PARP1-cb-RFP). PARP1 is known to have nucleolar functions where it acts as a scaffolding platform for the various nucleolar proteins aiding the ribosomal biogenesis (Boamah *et al*, 2012) We transfect U2OS cells with plasmid containing the chromobody and then treat the cells with 4-NQO and BMH21 and fix it. We find that cells treated with 4-NQO and BMH21 show lower nucleolar PARP1 intensity as compared to the control (Figure R4A). This can certainly result in lower baseline nucleolar PARylation and thus contribute to deacidification.

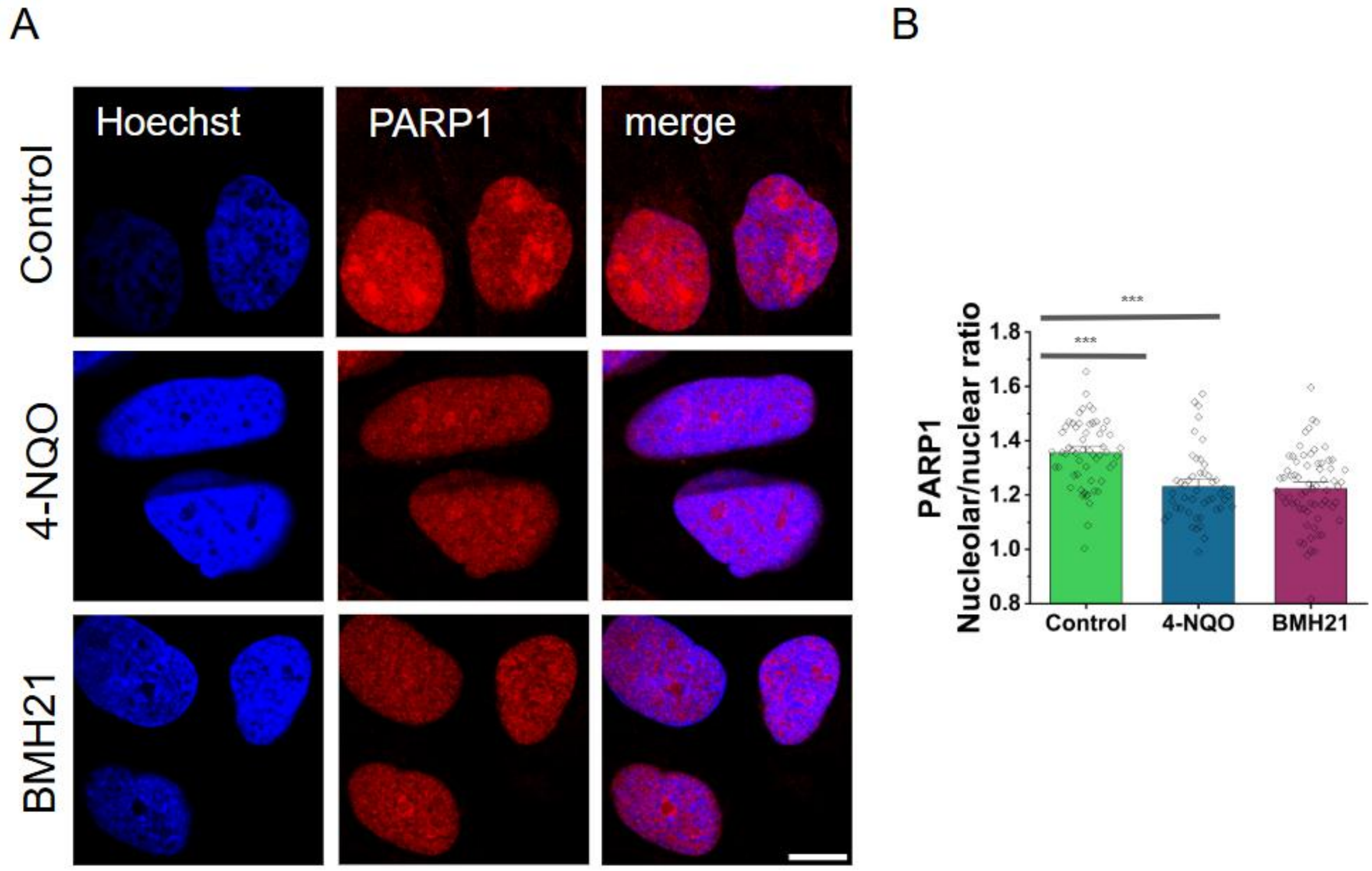

Figure R4: (A) U2OS Cells stably transfected with a PARP1-chromobody-RFP (red) and stained with Hoechst (blue) allows the visualization of endogenous PARP1. Higher nucleolar intensity in seen in control cells, which is lost following 4NQO and BMH21 treatment. (A) Ratio of mean nucleolar intensity to nucleoplasmic intensity of PARP1-chromobody-RFP are shown. n = 57,47,66 nuclei over three independent experiments. Data represents mean±*S.E.M from the three experiments. The comparisons are significant at p<0.0001 tested with a KS test (indicated by ***). Scale bar is 10µm.*

Further, we also present data about the translocation of the DE tract proteins. We show that among the three, NPM shows moderate decrease in nucleolar intensity, along with lower nucleolar to nucleoplasmic ratios indicating that it shuttles out into the nucleoplasm upon direct inhibition rRNA synthesis by BMH21 (Figure R5A-C). But NCL and Ki-67 shown significant

loss of nucleolar pool (Figure R5A, D and F-G) and increased nucleoplasmic pool (Figure R5A, E, F, and H) upon DNA damage by 4-NQO and inhibition of nascent rRNA synthesis by BMH21, adding to the evidence of local deacidification of the nucleolar core. Under homeostatic conditions these proteins are in multi-protein complexes with nascent pre-rRNA, to maintain a low-pH microenvironment. Upon DNA damage or transcriptional inhibition, the loss of rRNA results in deprotonation of these proteins due to electrostatic repulsion resulting in their outflux into the nucleoplasm. Together loss of rRNA and PAR levels and translocation of the DE tract proteins help address the point about nucleolar deacidification more comprehensively beyond just speculation. These different sources of deacidification may also explain the differences in the degree of deacidification between 4NQO and BMH21.

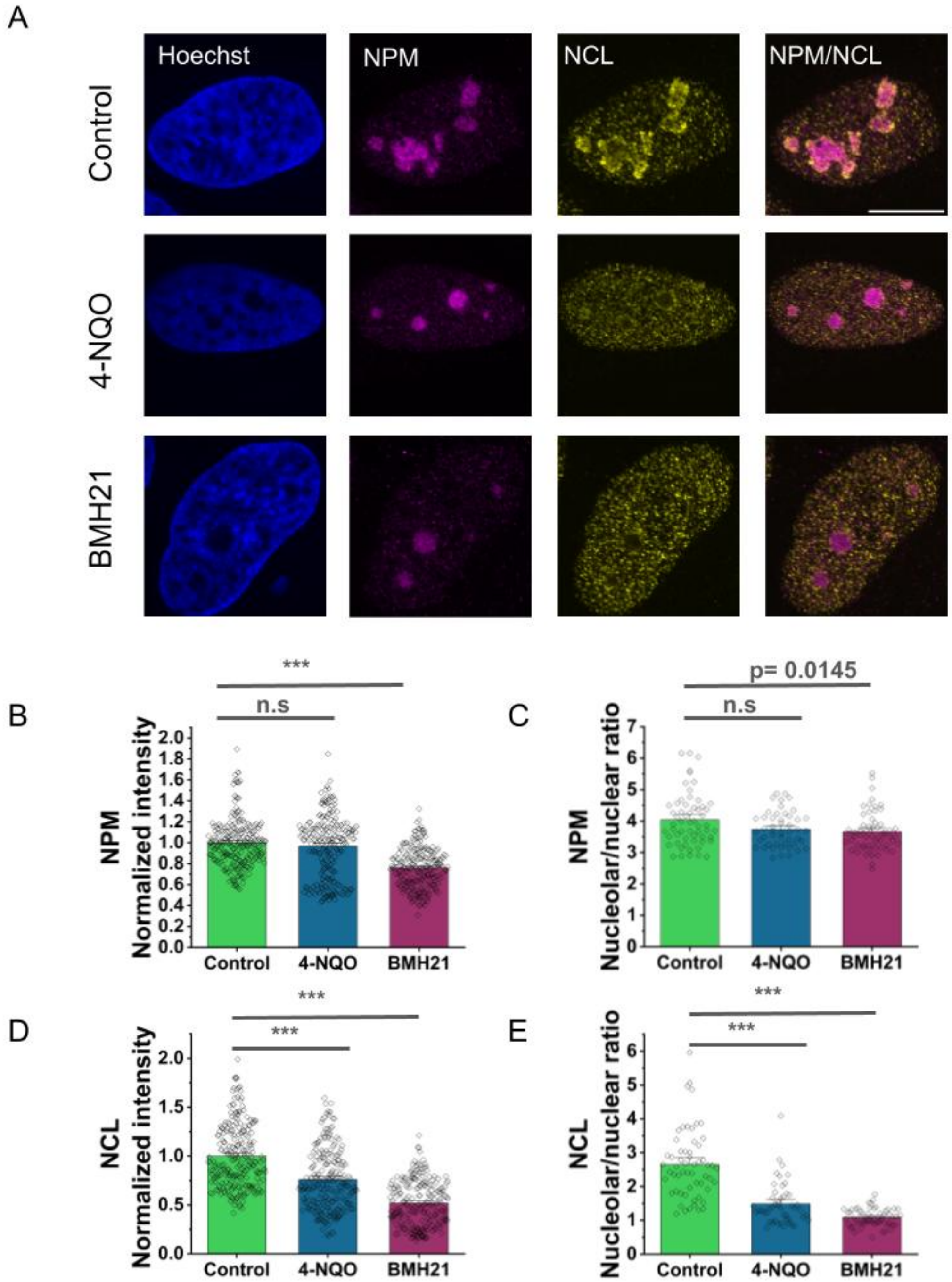

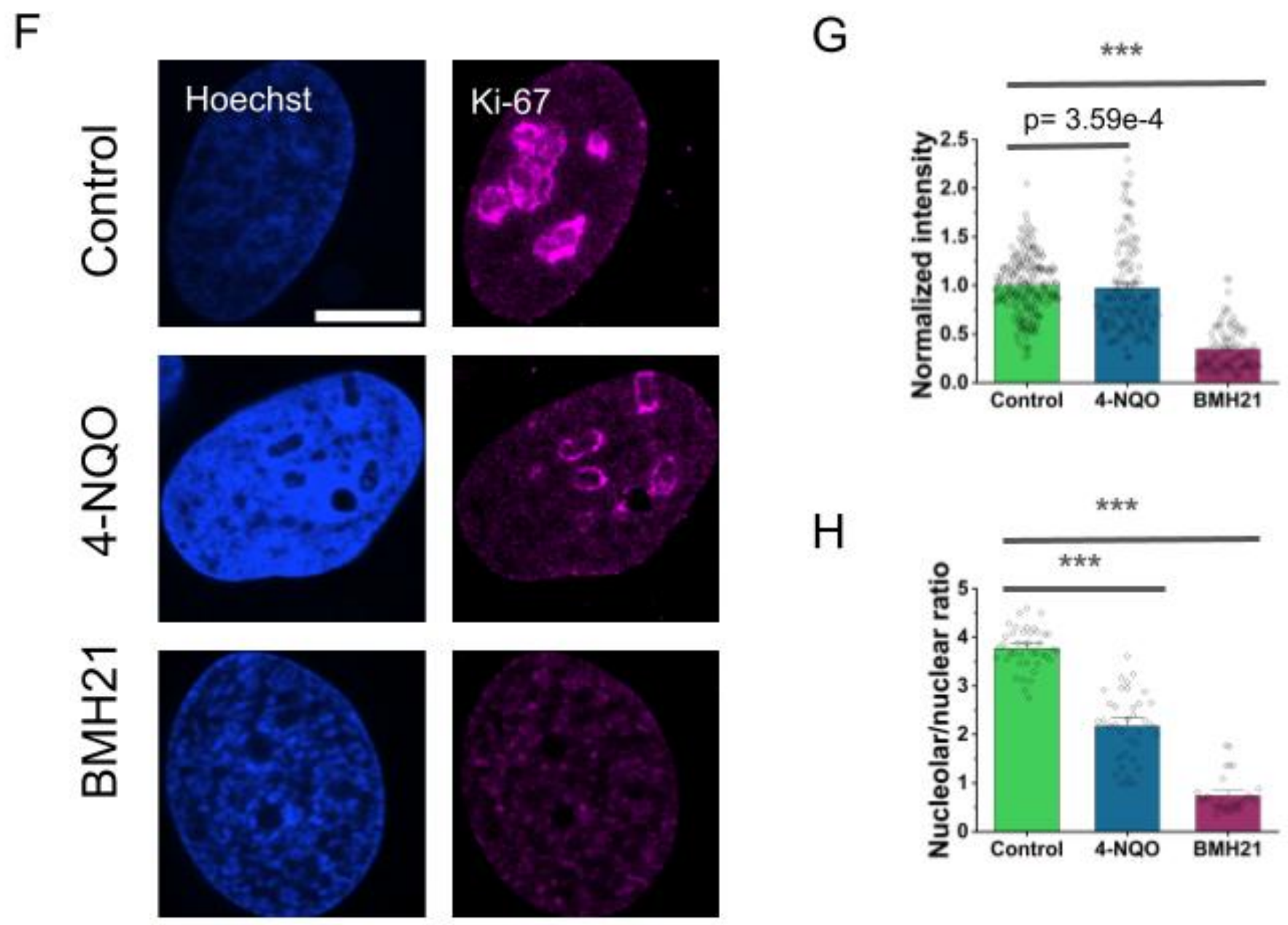

*Figure R5: (A) Nuclei from U2OS cells treated with 1ug/ml 4NQO for 1hr or 2uM of BMH21 for 3hrs, compared to control nuclei. Cells are stained with Hoechst (blue) and antibodies against NPM (magenta) and NCL (yellow). (B) Normalized nucleolar intensity of NPM, (C) Ratio between average nucleolar to nuclear (nucleoplasmic) intensity of NPM per cell. (D) Normalized nucleolar intensity of NCL, (E) Ratio between average nucleolar to nuclear (nucleoplasmic) intensity of NCL per cell. (F) Nuclei from U2OS cells treated with 1ug/ml 4NQO for 1hr or 2uM of BMH21 for 3hrs, compared to control nuclei. Cells are stained with Hoechst (blue) and an antibody against the Nucleolar Rim protein Ki-67 (magenta). The mean intensity of Ki-67 in individual nucleoli is plotted in (G), with the data for each experiment normalized to the mean of its corresponding control. (H) Ratio between average nucleolar to nuclear (nucleoplasmic) intensity per cell. Data represents mean± S.E.M. with n= 58, 48, 58 nuclei (A-E), n= 41, 36, 33 nuclei (F-G) over three independent experiments. Statistical significance calculated using K.S. test (*** represents p<0.0001).*

Minor concerns:

1. In Figure 3D, the labeling of UBF and rRNA appears to be reversed, which does not match the description in the main text.

2. In Figure 4, panel D is referenced in the text, but no Figure 4D is present.

Author response: We apologize for these oversights, and have rectified these.

Referee#2:

In this study, the authors utilize expansion microscopy to resolve changes in nucleolar architecture in response to DNA damage and transcription inhibition. They show that both DNA damage and inhibition of DNA synthesis alter nucleolar structure by reorganizing nucleolar layers and affecting transcriptional activity. In addition, the authors report that DNA damage or transcriptional inhibition is accompanied by an increase in nucleolar pH. Taken together, these results suggest that changes in nucleolar structure are largely driven by alterations in rRNA dynamics. Overall, the manuscript is well written, and the images are well presented and clearly described. While some of the observations reported here have been

previously described in the field, from this reviewer's perspective the data are compelling and provide an orthogonal approach through which models and insights into nucleolar biology can be tested. As such, and regardless of some overlap with existing literature, I strongly support publication of this study in EMBO. However, while the findings are of interest, the overall scope appears more appropriate for a short report than a full article. Overall, this study clarifies and adds important new insights to the field. Although I cannot evaluate the technical details of the expansion microscopy itself, from a nucleolar and cell biology standpoint this study is conceptually significant and merits publication. That said, there are several concerns that should be addressed by the authors prior to publication.

Author response: We thank the reviewer for their positive comments on our manuscript and address the specific points they raise below.

Major Concerns:

1• Several statements throughout the manuscript are misleading or inaccurate. While I highlight specific examples below, the authors should exercise greater care in how they present arguments about the field. The fact that a possibility has been proposed in a prior study does not constitute proof. Clear distinctions must be made between what is hypothesized, what is shown, and what is experimentally demonstrated. The presence of a citation alone should not be treated as a proxy for established ground truth.

Author response: We take this feedback seriously and appreciate the reviewer's call for greater precision in our scientific communication. We have reviewed the manuscript and recalibrated our language to clearly distinguish between hypothesized models and proven facts. We have also attempted to avoid any strawman arguments.

2• the authors claim to perform experiments under conditions of DNA damage; however, the manuscript does not provide direct evidence that DNA damage is actually occurring. At a minimum, the authors should demonstrate induction of γH2AX foci or provide other markers of DNA damage response pathway activation to substantiate these claims.

Author response: We agree that direct validation of DNA damage is essential to support our claims regarding the nucleolar response to genotoxic stress. To substantiate that 4-NQO indeed induces DNA damage under our experimental conditions, we have shown that 4-NQO causes significant induction of γH2AX in comparison to control and BMH21, indicating DNA damage (Figure R1 in this file).

We have also probed for nascent rRNA (5'ETS) multiplexed with DFC (Fibrillarin/FIB) and FC (UBF) proteins under other damage paradigms to score for the reorganization to show that the damage induced nucleolar cap formation of the DFC/FC proteins is indeed correlated to the levels of nascent rRNA. We used Neocarzinostatin (NCS)- a double-strand break inducer, Camptothecin (CPT) – Topoisomerase -I inhibitor eventually forming single and double strand breaks (Figure R6 in this file). At the doses used, CPT showed reduced nascent rRNA levels and increased nucleolar reorganization. For NCS where the rRNA intensity is unperturbed, the reorganization is also absent, further highlighting that rRNA levels affect the reorganization of the nucleolar compartments. We have now added this in the manuscript as a part of supplementary figure EV4.

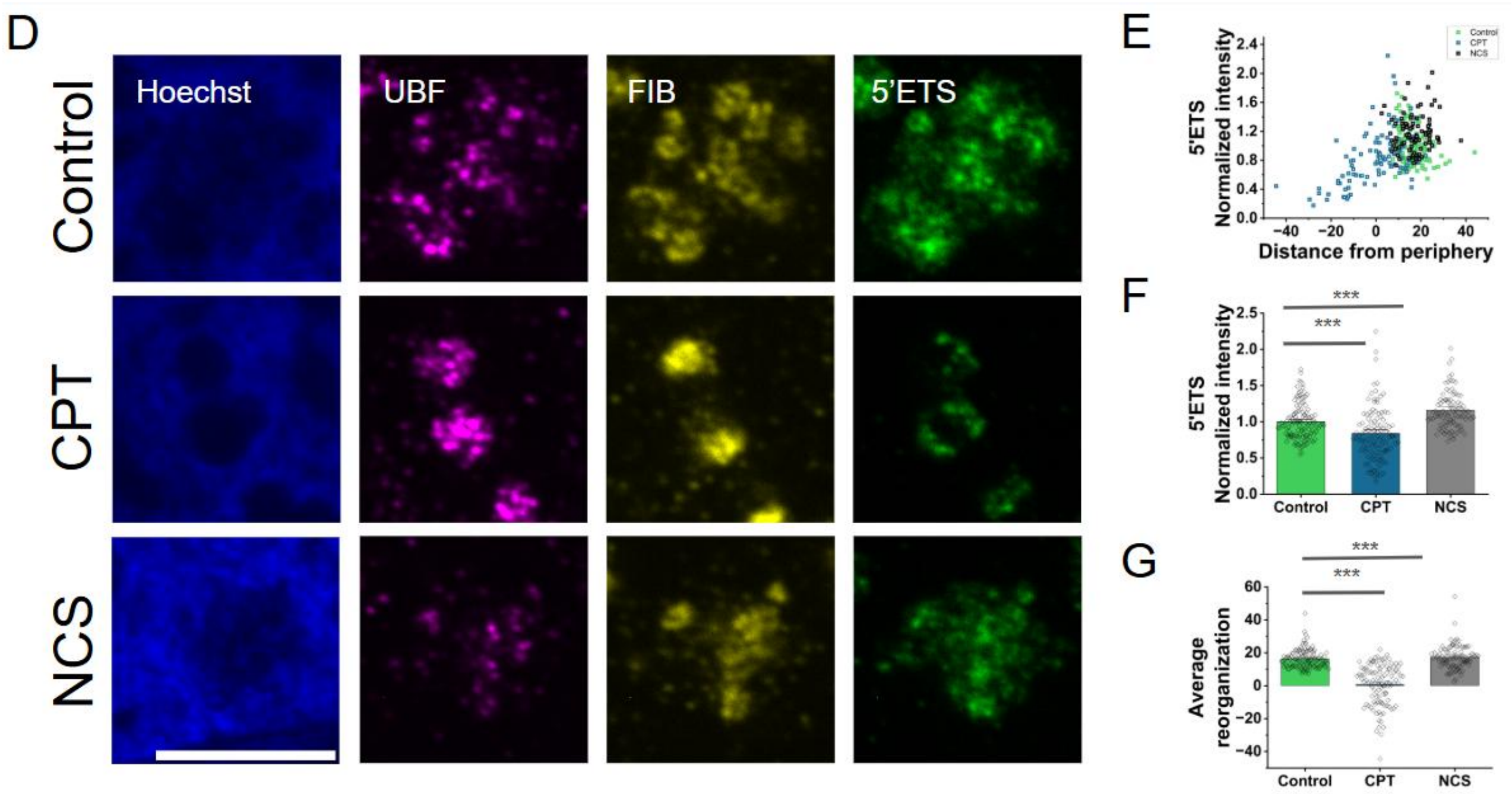

Figure R6: (A) Cells treated with Camptothecin (CPT, 25µM, 1hr), or Neocarzinostatin(NCS, 1µg/ml, 1hr) compared to control in DMSO. Hoechst (blue), UBF (magenta), Fibrillarin (yellow) and 5'ETS (green) signals are shown. (B) Mean 5'ETS intensity plotted against the mean distance of the UBF foci from the nucleolar periphery (in pixel units). Note how CPT datapoints show both lower 5'ETS and lower distance values indicative of reorganization, while the NCS datapoints effectively overlap with the control. (C) Normalized 5'ETS intensity from (B). (D) Average distance from the nucleolar periphery from (B). Data represent mean± S.E.M. Statistical significance calculated using K.S test, where *** is $p<0.0001$($n>=95$ nucleoli across 3 experiments). While the NCS data also turns out to be significantly different in a K.S. test given the high nucleoli numbers, the mean values are quite comparable the control cells. This is also seen in the scatter plot. Scale bar is 1µM.

3• The authors should perform a calibration curve for the pH measurements presented in Figure 6 or, alternatively, validate their findings using an independent chemical pH sensor. Such validation is necessary to strengthen confidence in the reported changes in nucleolar pH.

Author response: We have added the pH calibration curve to show our validation of the pH sensor and have mentioned it in the main manuscript (Figure 6) as well.

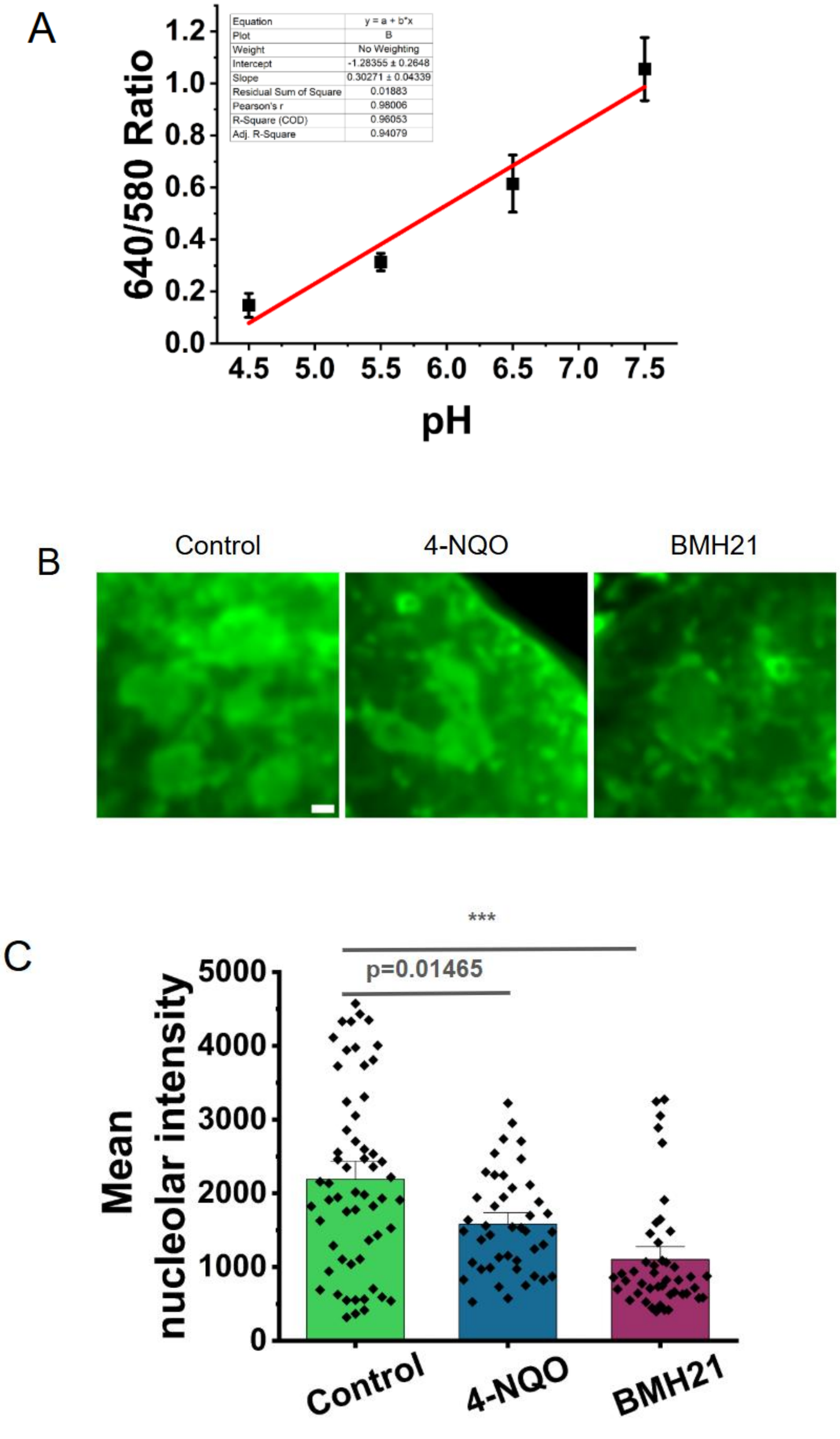

*Figure R7: (A) pH calibration curve for SNARF-4F. Data represents mean± standard deviation (S.D), n>=270 (302;277; 320;373) nucleoli over three independent experiments. (B) pHrodo-AM incubated in U2OS cells (1:1000) for 30 minutes (C) Nucleolar intensity of pHrodo-AM computed over n>=43 nucleoli. Data represents mean ± S.E.M, with significance tested with K.S.test (*** represents p<= 0.0001). Scale bar is 1μm.*

This is in line with the pH calibration data in(Aryan *et al*, 2023) and Pol I inhibition shows a level of deacidification beyond the range of this graph in that study too. We have also used another pH sensor dye called pHrodo-AM which shows higher fluorescence with lower pH. We observe that relative nucleolar intensity decreases upon DNA damage with 4-NQO and transcriptional inhibition by BMH21, suggesting nucleolar deacidification (increasing pH).

Other Comments:

1• Please revised this misleading statement "However, the effects of cellular stress or disrupted transcription on nucleolar protein and RNA organization are still not well understood, limited both by the resolution of light microscopy, and lack of tools for detection of transcript species of interest and complex sample preparation in electron microscopy." Indeed, this an unresolved question. But the issue is resolution in general and not necessarily limited by resolution of light microscopy, the issue is more complex than that.

2• Please note that the following statement is inaccurate and misleading "The outflux of newly synthesized rRNA is facilitated by a combined action of K-block and D/E tracts enriched nucleolar proteins. The K-blocks create a scaffold of transient multivalent interactions that aid in nucleolar organization, while the D/E tracts serve as proton carriers that set up a pH gradient, both contributing to the directional export of processed rRNA." The outflux of rRNA is more complex than described and emerging studies suggest that K-block and D/E tracks containing-proteins contribute. However, this statement ignores the multitude of other process, molecules and biochemical reactions that contribute to this process overall. Therefore, this statement needs to be revised to reflect the facts and what is known and what is just a hypothesis. Similarly, it has been suggested that D/E tracks might serve as proton carriers, but this has not been formally proven and so it should not be state as ground truth.

3• This statement needs references "suggested that the rRNA transcription is initiated at the interface of FC-DFC with co-transcriptional processing, triggering phase separation and rRNA sorting into the subsequent compartments." This has been the prevailing view in the field for the last decade.

4• This statement is completely FALSE/Disproven "It is known that the rRNA leads to the nested tripartite structure of nucleoli (Farley et al, 2015; van Sluis & McStay, 2017) and direct or indirect inhibition of transcription leads to the nucleolar reorganization leading to its segregation or reoriented bipartite structure." The bipartite structure of the nucleolus is defined as the nucleoli structure in non-amniotes organism. It has absolutely nothing to do with the nucleolus under stress. Secondly, three reports have shown that the tripartite structure of the nucleolus is mediated by Treacle.

5• This statement is in principle inaccurate please revise "Condensates formed by RNA or protein or both, get seeded by upon increasing concentration and affinity towards each other"

6• This statement is inaccurate and misleading please revised "This is because rRNA can scaffold multivalent interaction among the Glycine-Arginine rich (GAR) resident proteins which, when altered, shifts to GAR-GAR interacting condensates making it more viscous or dense in nature"

Author response: We take this feedback seriously and appreciate the reviewer’s call for greater precision in our scientific communication. We do not mean to misrepresent the literature in the

field. We have comprehensively reviewed the manuscript, and revised the above statements as appropriate.

Referee #3:

In their manuscript "Nucleolar reorganization on stress depends on physicochemical changes due to nascent rRNA synthesis" the authors use advanced fluorescence microscopy techniques (IF, RNA FISH, FRAP, fluorescence pH sensor in combination with expansion microscopy and SoRa super-resolution imaging) to observe changes in the nucleolus, particularly after treatment with 4-NQO and BMH-21. 4-NQO forms covalent adducts at purine bases, triggering a DNA damage response that indirectly leads to Pol I inhibition. BMH-21 acts as an intercalator of GC-rich DNA without a DNA damage response. Unlike 4-NQO and other Pol I inhibitors such as CX-5461, it causes rapid dissociation of Pol I from rDNA and subsequent proteasome-dependent degradation of the catalytic RPA194 subunit within 2 to 3 hours.

Although the topic is interesting, the claims often lack novelty and originality, and in some cases they are supported more convincingly by previously published results than by the authors' own findings (e.g. "Stress-induced transcription inhibition remodels the sub-nucleolar compartments from a viscoelastic state into solid-like condensates thereby perturbing the nucleolar pH gradient due to the missing rRNA scaffold" in the Abstract). The Title and Abstract promise a significant deepening of the mechanistic understanding of nucleolar reorganization under stress conditions. In fact, the general term "stress" mainly encompasses treatments with the two inhibitors (without systematically comparing them with other DDR-inducing or DDR-independent Pol I inhibitors). Monitoring of physicochemical changes is limited to pH measurements. The role of nascent RNA synthesis is extrapolated from FISH localization studies on pre-rRNA. Another important general criticism is that the context provided by the earlier literature is not adequately presented. Essential information available in the cited literature is not always correctly mentioned or is not detailed enough.

Given the complementary nature of the findings to previous studies (in particular these cited works: Yao et al, 2019, Mol Cell. Nascent Pre-rRNA Sorting via Phase Separation Drives the Assembly of Dense Fibrillar Components in the Human Nucleolus; Quinodoz et al, 2025, Nature. Mapping and engineering RNA-driven architecture of the multiphase nucleolus - the reference has to be properly formatted in the References; Alley et al, 2025, ACS Chem Biol.Expansion Microscopy Provides Nanoscale Insight into Nucleolar Reorganization and Nuclear Foci Formation during Nucleolar Stress) and the lack of in-depth analyses with orthogonal validations, the manuscript would be more suitable for a specialised cell biology journal than for EMBO Reports, which is why I cannot recommend it for publication.

Author response: We thank the reviewer for their comments and have now revised the manuscript to bring out the novelty of our results with respect to the recent studies. Many new experiments have been added in response to reviewers' queries, and we believe the manuscript is much stronger for these changes; we hope the reviewer will agree with us. We once more thank them for their comments and address their concerns below.

Major points

1. The Introduction section should provide an appropriate introduction to various forms of nucleolar stress and clearly state the stress inducers used in the study (4-NQO and BMH-21) and their mechanism of action. Two recent review articles may be helpful here: Imamura R, Yasuhara T. Nucleolar Organization in Response to Transcriptional Stress. Cancer Sci 116, 2649-2656 (2025). Cerezo EL, Henry Y, Henras AK, Romeo Y. Ribosome biogenesis and homeostasis: in the front line to cope with cellular stress. Biochimie 238, 43-56 (2025). Furthermore, it must be clarified and emphasized that only the first steps of ribosome synthesis take place in the nucleolus and that pre-ribosomal particles undergo further maturation steps in the nucleoplasm and cytoplasm (see Vanden Broeck and Klinge 2024 from the cited literature). Also, the statement on page 8 "The modified RNA then diffuses to GC where they assemble with the ribosomal subunits to form mature ribosomal subunits (Dogra & Kriwacki, 2025)." has to be corrected. The multistep process of ribosome assembly is not completed in the GC.

Author response: We thank the Reviewer for these specific suggestions to improve the Introduction and Results sections. We agree that a more nuanced discussion of nucleolar stress and the spatial constraints of ribosome maturation is necessary. We have expanded the Introduction with details of nucleolar Stress and provided a clearer rationale for our choice of stress inducers and their function. We have further discussed ribosome biogenesis stages and assembly sites. We have now investigated the effects of other DNA damage inducers (Figure R6) to substantiate the generality of our claims.

2. The authors state on pages 4 and 7: "But how the rRNA outward flux is spatially altered within the nucleolar sub-compartments in healthy versus stressed cells is still elusive,..." "Although it is known that transcriptional inhibition and DNA damage leads to nucleolar reorganization(Boulon et al., 2010; Shav-Tal et al, 2005; Thomas Haaf, (1996); van Sluis & McStay, 2017), how the rRNA synthesis and maturation impact nucleolar phases and gate the outward rRNA flux is poorly understood."

I cannot agree with this, as the publication 'Mapping and engineering RNA-driven architecture of the multiphase nucleolus' by Quinodoz et al. 2025 in Nature revealed a considerable amount of detail using complementary experimental approaches (SRM + NGS) at a very high technical level. They used chemical inhibition of rRNA processing (FVP) and, above all, a series of genetic inhibitions that provide clearer insights into the factors and mechanisms involved.

Author response: We appreciate the Reviewer’s concern regarding the distinction between published work and our data. We specifically wanted to highlight that the correlation between spatially altered rRNA outflux in the context of damage is less studied. But have reviewed the language and arguments to make the narrative more technically sound.

3. The authors write on page 6: "A very recent study has indeed reported the use to expansion microscopy to study nucleolar structures (Alley et al., 2025), but beyond just reporting the structural details under normal homeostasis, we further investigated how it is reorganized under stress and the roles of rRNA in such reorganization". The results related to signal intensity and localization studies of rRNA using RNA-FISH of the 5'-ETS and 28S probes do not explain the role of rRNA in stress-dependent reorganization of the nucleolus. Although they may be

related, this also applies to numerous other nucleolar factors that were not investigated in the experiments and could be causal. A particularly interesting candidate would be RPA194, which is rapidly degraded when treated with BMH-21, but not when treated with other Pol I inhibitors. It should also be emphasized that Alley 2025 also applied stress.: "We investigate time-dependent changes in nucleolar structure and function under nucleolar stress induced by oxaliplatin, actinomycin D, and other platinum-based compounds."

Author response: We thank the reviewer for this critical perspective on the causality of nucleolar reorganization. We agree that while rRNA depletion is a hallmark of the observed structural changes, it is essential to consider the role of the transcriptional machinery itself, specifically the RNA Polymerase I complex. Therefore, we show that RNAPI (RPA194) signal is not completely lost upon treatment with BMH21 (Figure R1 in this document), but localizes to nucleolar caps. This highlights that perhaps not the degraded machinery itself, but the stalling of the RNAPI because of BMH21, is what leads to reduced rRNA levels and hence nucleolar reorganization.

We have also revised our manuscript to properly credit Alley et al. 2025 and discuss how our approach complements their findings. What we meant was that there was no detection of specific rRNA species relating to stress induced nucleolar reorganization in that study. We apologize that this did not come across and have modified our manuscript to reflect this.

1. On page 9, part of the text is very vague and partly incomprehensible: "UBF1 is in itself a 3D-shell like structure" probably refers to the 3D structure of individual active rDNA units bound with UBF, rather than the structure of UBF1 itself. The meaning of this sentence is unclear: "Co-stained FC-DFC modules (using UBF1 and FIB) along with 5'ETS to understand the spatial localization of the nascent pre-rRNA showed that the pre-rRNA partially overlapped with FIB seen in the images as well as the line profile (Figure 3E, F)." The causal relationship between the two parts of the next sentence is not substantiated by the data provided: "The partial interspersion of 5'ETS with FIB signals (Figure 3F) suggested that the rRNA transcription is initiated at the interface of FC-DFC with co-transcriptional processing, triggering phase separation and rRNA sorting into the subsequent compartments." Thorough analyses using shRNA-mediated depletion of 17 factors and quantitative SIM using the 5'-ETS-1 RNA FISH probe plus mEGFP-RPA194 knock-in as an FC marker by Yao et al. 2019, revealed the emerging 47S pre-rRNA transit from the FC/DFC border to DFCs and the factors involved in much greater detail. Those data suggest "sorting into the subsequent compartments" (transit as they call) more convincingly. The data presented here support these findings well using a simpler experimental setup, without addressing the possible involvement of selected nucleolar proteins in this process through loss-of-function analyses.

Author response: We thank the Reviewer for their feedback on the language. We have now discussed the roles of other nucleolar proteins (Se Figure R6 in this document). We have reviewed the manuscript, and refined our causal claims with evidence and references.

5. Page 10 "Earlier reports suggested that the protein reorganization in both silencing of RNAPI-mediated transcription and DNA damage are similar (Franek et al, 2016), but we show that the spatial localization and levels of nascent rRNA can vary in case of DNA damage and transcriptional stress, perhaps related to the level of rRNA abrogation." Franek et al., 2016 did not use BMH-21, but ActD. As originally described in Peltonen et al.,

2014 PMID: 24434211 (should also be cited), BMH-21 causes different changes in nucleolar reorganization compared to ActD. It is not appropriate to cite the study by Franek et al., which uses a less specific RNA Pol I transcription inhibitor, without mentioning this.

Author response: We appreciate the Reviewer's correction regarding the citation of earlier work (Franek *et al*, 2016). We acknowledge that the nucleolar reorganization induced by Actinomycin D (ActD) and BMH21 is mechanistically and morphologically distinct due to their different modes of action. While both Actinomycin D (Act D) and the small molecule BMH21 function as DNA-intercalating transcription inhibitors, their downstream genotoxic profiles diverge significantly. Actinomycin D can induce γH2AX foci (Mischo *et al*, 2005), reflecting the accumulation of DNA double-strand breaks typical of conventional intercalators. Conversely, BMH21-mediated inhibition of RNA polymerase I has been well-documented to suppress transcription and trigger RPA194 degradation without eliciting a γH2AX-positive DNA damage response (Colis *et al*, 2014), highlighting a distinct, non-genotoxic mechanism of RNA Pol I inhibition and nucleolar stress induction. We too find BMH21 does not induce γH2AX and RPA194 signal is not entirely lost (Fig R1 in this document). We have revised the manuscript to clarify the same.

6. While the control samples and the 4-NQO samples (1h treatment) in Fig. 4 show clear differences in the UBF and 5'-ETS signals, there is no obvious difference between these settings in Fig. 5, raising questions about the reproducibility of the treatment effect or the robustness of the analytical method.

Author response: We take the reviewer's concerns seriously and have revised the manuscript to rectify for any perceived discrepancies between the images and quantification. All the experiments have been performed multiple times (N=3 at least unless otherwise indicated) as mentioned in the figure legends and the results are robust. A statement has been added to the 'Statistics' section about this too.

7. In the metric used to quantify stress-induced nucleolar reorganization using 5'-ETS RNA-FISH analyses, the Results section does not mention what the marker for the "nucleolar periphery/nucleolar perimeter" is (probably 28S, based on the Methods section, but this should at least be briefly mentioned in the Results section). More importantly, even in the Methods section, it remains unclear how the third dimension (Z-axis) and the unevenness of the "periphery/perimeter" are taken into account. Without clarification of the latter points, the numerical assessments and distance measurements remain ambiguous.

Author response: We acknowledge the reviewer's concern regarding the detailing of the methods. We have revised the manuscript to provide the analysis greater details and clarity. The nucleolus is marked using Hoechst channel while analyzing data related to 5'ETS and UBF positioning, while in case of experiments where 28S or NPM was stained, those channels were used for the ease of segmentation. This is now added to the Supplementary Information file (Appendix Figure 1and 2).

8. The use of transiently overexpressed GFP-UBF raises concerns and severely limits the explanatory power of the otherwise sound FRAP approach. First, the amount of overexpressed UBF strongly influences its localization. Second, the presence of endogenous UBF creates competition for rDNA binding. These problems can be solved most elegantly with an endogenous tagging approach, as used in the study by Yao et al. (2019). In addition, when using

BMH-21, the induced degradation of RPA194, an interaction partner of UBF, should also be taken into account. Therefore, it remains unclear whether the effects on UBF localization and dynamics are due to the inhibition of rRNA synthesis, the loss of RPA194, or a combination of both, or something else. The causal relationships are highly speculative and are not sufficiently supported by the FRAP data. They could be discussed to some extent in the "Discussion" section.

Author response: We agree that FRAP data of GFP-UBF, while indicative of reduced molecular exchange, alone does not fully define the material state of the nucleolus and endogenous tagging (as demonstrated by **Yao et al. 2019**) is better for avoiding artifacts related to protein stoichiometry and competition for rDNA binding sites. However, we are comparing among similarly transfected cells under different conditions, so our results still hold. Cells with similar expression were chosen. We agree that the degradation of RPA194 by BMH21 can complicate interpretation. If the scaffold for UBF (the RNA Pol I complex) is degraded, its dynamics will change, but should in fact become faster. We have updated the manuscript to add that the data may be interpreted as a combined effect of both loss of nascent rRNA and the physical dissolution of the RNA Pol I-RPA194 scaffold. Using immunofluorescence assays, we have shown that RPA194 is not completely degraded by dosage and time of BMH21 used in our studies, but rather form nucleolar caps (Figure R1A). In order to further our claims, we performed fluorescence anisotropy measurements of cells transiently transfected with GFP-UBF under conditions of DNA damage by 4-NQO and transcriptional perturbation of rRNA by BMH21 providing additional biophysical evidence of a transition to a low mobility potentially solid-like state (see Figure R2 here and the associated discussion). We observed that cells treated with BMH21 has higher anisotropy (Figure R2A) than both control and cells treated with 4-NQO, suggestive of a slower rotational diffusion. The lower rotational motion is in line with the observation from our FRAP assays.

9. The pH measurements using the SNARF-4F AM ester as a ratiometric pH sensor provide interesting results, but further investigation would be required to support well-founded conclusions. The cited study by Aryan et al. from 2023 reports very significant deacidification during Pol I inhibition using ActD and CX5461. BMH-21-dependent Pol I inhibition should be compared with ActD/CX5461-dependent Pol I inhibition (and a control measurement of the inhibitory effect should be performed). Orthogonal validation of the pH measurements (e.g., with a genetically encoded sensor with NoLS) would also be important due to the known limitations of SNARF-4F (potential cytotoxicity, low SNR).

Author response: We thank the Reviewer for this rigorous assessment of our pH measurements. The distinction between different RNA Pol I inhibitors is critical, and will therefore validating the SNARF-4F results with established benchmarks and orthogonal methods is necessary for a well-founded conclusion. Previous studies have reported relative changes, but SNARF fluorescence ratios can be also calibrated to absolute pH values. We have ourselves verified this, and have provided that necessary data (Figure R7). SNARF-4F has been used for studies on nucleolar pH by several studies before (Aryan *et al.*, 2023; King *et al*, 2024) and we chose this because it is the most standard pH detector in this case.

Minor points

1. The placement of background information requires improvement: the Results section contains several paragraphs, some of which should be included in the Introduction and others in the Discussion section.

2. With regard to the work of van Sluis and McStay, not only their review article should be cited, but also the authors' primary work PMID: 26019174.

3. On Fig 3A the rDNA unit is not in scale.

4. For better understanding, the rRNA-making factories on page 15 should be termed actively transcribed rDNA units.

5. Page 11 "The cell nucleolus is emerging as a stress sensor within the cells (Yang et al., 2018). It has gained popularity as a potential biomarker for pathological conditions of cancer and neurodegeneration in the past decade." The fact that enlarged and irregular nucleoli are characteristic features of cancer cells is known since the late 19th century and has been used by pathologist since many decades.

Author response: We have now addressed these issues in the manuscript. We thank the reviewer for their careful reading of our manuscript and the important points they bring up.

References

Aryan F, Detres D, Luo CC, Kim SX, Shah AN, Bartusel M, Flynn RA, Calo E (2023) Nucleolus activity-dependent recruitment and biomolecular condensation by pH sensing. *Mol Cell* 83: 4413-4423 e4410

Boamah EK, Kotova E, Garabedian M, Jarnik M, Tulin AV (2012) Poly(ADP-Ribose) polymerase 1 (PARP-1) regulates ribosomal biogenesis in Drosophila nucleoli. *PLoS Genet* 8: e1002442

Bose M, Lampe M, Mahamid J, Ephrussi A (2022) Liquid-to-solid phase transition of oskar ribonucleoprotein granules is essential for their function in Drosophila embryonic development. *Cell* 185: 1308-1324 e1323

Colis L, Peltonen K, Sirajuddin P, Liu H, Sanders S, Ernst G, Barrow JC, Laiho M (2014) DNA intercalator BMH-21 inhibits RNA polymerase I independent of DNA damage response. *Oncotarget* 5: 4361-4369

Coria AR, Shah A, Shafieinouri M, Taylor SJ, Orgebin E, Guiblet W, Miller JT, Sharma IM, Wu CC (2025) The integrated stress response regulates 18S nonfunctional rRNA decay in mammals. *Mol Cell* 85: 787-801 e788

Ferreira R, Schneekloth JS, Jr., Panov KI, Hannan KM, Hannan RD (2020) Targeting the RNA Polymerase I Transcription for Cancer Therapy Comes of Age. *Cells* 9

Franek M, Kovarikova A, Bartova E, Kozubek S (2016) Nucleolar Reorganization Upon Site-Specific Double-Strand Break Induction. *J Histochem Cytochem* 64: 669-686

James A, Wang Y, Raje H, Rosby R, DiMario P (2014) Nucleolar stress with and without p53. *Nucleus* 5: 402-426

Kesavan PS, Bohra D, Roy S, Mazumder A (2020) Monitoring global changes in chromatin compaction states upon localized DNA damage with tools of fluorescence anisotropy. *Mol Biol Cell* 31: 1403-1410

King MR, Ruff KM, Lin AZ, Pant A, Farag M, Lalmansingh JM, Wu T, Fossat MJ, Ouyang W, Lew MD *et al* (2024) Macromolecular condensation organizes nucleolar sub-phases to set up a pH gradient. *Cell* 187: 1889-1906 e1824

Kruhlak MJ, Celeste A, Dellaire G, Fernandez-Capetillo O, Muller WG, McNally JG, Bazett-Jones DP, Nussenzweig A (2006) Changes in chromatin structure and mobility in living cells at sites of DNA double-strand breaks. *J Cell Biol* 172: 823-834

Lakowicz JR (2006) *Principles of fluorescence spectroscopy*. Springer, New York

Lin Y, Protter DS, Rosen MK, Parker R (2015) Formation and Maturation of Phase-Separated Liquid Droplets by RNA-Binding Proteins. *Mol Cell* 60: 208-219

Ma H, Pederson T (2013) The nucleolus stress response is coupled to an ATR-Chk1-mediated G2 arrest. *Mol Biol Cell* 24: 1334-1342

Mischo HE, Hemmerich P, Grosse F, Zhang S (2005) Actinomycin D induces histone gamma-H2AX foci and complex formation of gamma-H2AX with Ku70 and nuclear DNA helicase II. *J Biol Chem* 280: 9586-9594

Patel A, Lee HO, Jawerth L, Maharana S, Jahnel M, Hein MY, Stoynov S, Mahamid J, Saha S, Franzmann TM *et al* (2015) A Liquid-to-Solid Phase Transition of the ALS Protein FUS Accelerated by Disease Mutation. *Cell* 162: 1066-1077

Rancourt A, Satoh MS (2009) Delocalization of nucleolar poly(ADP-ribose) polymerase-1 to the nucleoplasm and its novel link to cellular sensitivity to DNA damage. *DNA Repair (Amst)* 8: 286-297

Saha S, Anilkumar AA, Mayor S (2016) GPI-anchored protein organization and dynamics at the cell surface. *J Lipid Res* 57: 159-175

Shav-Tal Y, Blechman J, Darzacq X, Montagna C, Dye BT, Patton JG, Singer RH, Zipori D (2005) Dynamic sorting of nuclear components into distinct nucleolar caps during transcriptional inhibition. *Mol Biol Cell* 16: 2395-2413

Wei J, Zhang Y, Ye B, Fan X, Hu Y, Xiang S, Ma W (2024)

Yin H, Jiang Z, Wang S, Zhang P (2019) Actinomycin D-Activated RNase L Promotes H2A.X/H2B-Mediated DNA Damage and Apoptosis in Lung Cancer Cells. *Front Oncol* 9: 1086

1st Revision - Editorial Decision 5th Aug 2026

Dear Prof. Mazumder,

Thank you for the submission of your revised manuscript. We have now received the enclosed reports from the referees and I am happy to say that all support its publication now. Referees 1 and 3 still have a few more suggestions that I would like you to address and incorporate before we can proceed with the official acceptance of your manuscript.

A few editorial requests will also need to be addressed:

- The sentence in the Data Availability Section regarding the Source Data should be removed and instead this sentence: "This study includes no data deposited in external repositories" should be provided.

- The conflict of interest subheading needs to be corrected to "Disclosure and Competing Interests Statement" (DCIS). Employment in a biotech company should be stated in the DCIS.

- All supplementary figures can be provided as Expanded View (EV) figures that need to be uploaded as individual, production quality figure files. It is fine to have 8 EV figures. The EV figure legends need to be provided after the main figure legends in the main ms file; the correct nomenclature is Figure EV1, etc. Please correct all file names and the callouts to the EV figures.

- A callout is missing for Figure 7, please add.

- The Reagents & Tools TABLE needs to be removed from the ms and uploaded as separate file using the same (Reagents table) file type; callouts to the tables need to be corrected. All tables in the ms file should be part of the Reagents and Tools table.

- The Source Data need to be uploaded as one file per main figure and the readme file can be added to the Figure 5 folder.

- Number of words/figures/ references, etc. should be removed from the title page

- Materials and Methods should be Methods

* Figure Legends - Comments *

- Please note that the exact p values are not provided in the legends of figures 4B,C,D,E,F; 5D,E; 6C,D; EV-1A; EV-3B,F,G,H,J,K; EV-4B,C,F,G; EV-5D; Appendix Figure 3B. Pease provide exact values as reasonable.
- Please note that for the figure 3, p-values and statistical tests are indicated in the legends. However, comparison for the same, ""****/n.s"" has not been represented in the figures. Please rectify this in the figures or legends as applicable.
- Please note that information related to n is missing in the legends of Appendix Figure 3B.
- Please note that the scale bar is missing for figures 2B,C.
- Please note that the scale bar needs to be defined for figures EV-2A,B,C; EV-3A,D,E.
- Please note that in legends of figures EV-4A,D the scale bar unit should be corrected from µM to µm.

I would like to suggest some minor changes to the title and abstract that needs to be written in present tense. Do you agree with this:

Nucleolar reorganization upon stress depends on physicochemical changes due to reduced nascent rRNA synthesis [OK?]

Successive maturation of ribosomal subunits occurs through multilayered phase-separated structures of the cell nucleolus. The spatio-functional relationship between transcription of rRNA and nucleolar substructures, and how this adapts to cellular stress remain incompletely understood. In this study, we resolve the sub nucleolar structures using expansion microscopy to reveal ordered structures of fibrillar center (FC) and dense fibrillar component (DFC) domains as nested shells, which are reorganized upon cellular stress like DNA damage or RNA polymerase I (RNAPI) inhibition. Direct visualization of nascent (5' ETS) and mature (28S or 18S) rRNA suggests that rRNA synthesis is a critical regulator of nucleolar size and organization. Nucleolar reorganization upon stress emerges to be a direct function of nascent rRNA levels. Stress-induced transcription inhibition can remodel the sub-nucleolar compartments to a low mobility state and perturbs the nucleolar pH gradient due to the missing rRNA scaffold and other factors. We show that rather than signaling to mediate rDNA repair, nucleolar reorganization arises naturally from reduced rRNA levels and the resultant biophysical restructuring of the nucleolus under cellular stress.

EMBO press papers are accompanied online by A) a short (1-2 sentences) summary of the findings and their significance, B) 2-3 bullet points highlighting key results and C) a synopsis image that is exactly 550 pixels wide and 300 pixels high. The synopsis image should provide a sketch of the major findings, like a graphical abstract. Please note that text needs to be readable at the

final size. Please send us this information along with the final manuscript.

I look forward to seeing a final version of your manuscript as soon as possible.

Kind regards,
Esther

Esther Schnapp, PhD
Senior Editor
EMBO reports

Referee #1:

The authors have addressed most of the previous questions, and the manuscript has been substantially improved. Before publication, a couple of minor concerns still require clarification: the nucleolar morphology after 1 h of 4-NQO treatment appears to be inconsistent between Figure 2b and Figure 5a. In Figure 2b, the nested FC/DFC structure has been disrupted, whereas in Figure 5a the structure appears largely unaffected. The authors should carefully verify these data and provide a critical assessment of the actual effects of 4-NQO treatment on nucleolar organization. Also, in Figure 5a, the UBF signals are overexposed, making it difficult to accurately evaluate their localization and morphology. Please adjust the image display parameters to better reflect the original fluorescence signal.

Overall, provided that the above concern is satisfactorily addressed, no further concerns remain regarding publication.

Referee #2:

This study uses expansion microscopy to understand how nucleolar organization is influenced during rDNA transcription downregulation and/or exposure to rDNA-damaging agents, with a specific emphasis on how rRNA levels alters the biophysical properties of the nucleolus, namely its viscoelasticity and pH. The authors have largely addressed my previous concerns, and I have no additional concerns.

I will reiterate that, although some of the data presented here confirm and only marginally expand our current understanding of how the nucleolus reorganizes under different conditions, this study is nonetheless important and significant, and I therefore support its publication in EMBO. This work will be well appreciated by the field in light of emerging mechanisms surrounding nucleolar biophysical homeostasis, function, and architecture.

Referee #3:

The authors have carefully addressed all my comments and present a considerable number of new experiments to further substantiate and expand upon their findings. These include pHrodo-AM, yH2AX, 18S and PARP1 analyses, the translocation of DE-tract proteins, NCS and CPT treatments, and fluorescence anisotropy imaging (FAI) of GFP-UBF to investigate local order, viscosity and rotational constraints. In addition, they implemented extensive amendments to the text in line with the suggestions and criticisms. This impressive effort, undertaken within the short timeframe of the peer-review process, is greatly appreciated.

Although the novelty of the study is still limited, the revised, extended version complements earlier findings well. The revised version also refers to these findings more appropriately and presents them more clearly. In my opinion, the use of GFP-UBF still represents a limitation, despite the described selection of cells with comparable expression levels; however, the study's main conclusions do not depend exclusively on the GFP-UBF analyses, so this limitation is acceptable.

Minor points

1. In the PARP1 analysis (Supplementary Figure EV5), nucleoli are presumably identified as described in Appendices 1 and 2. The risk associated with using Hoechst exclusion staining as a substitute for nucleolar staining is that nuclear bodies (Cajal bodies, nuclear spots, paraspeckles) are also only weakly stained and cannot be distinguished from smaller nucleoli. The cell nucleus shown in Appendix 2 illustrates this problem very well. It would be useful to specify the nucleolus selection criteria (size?) and briefly discuss this point in the 'Methods' section, as it is crucial for calculating the nucleolar/nuclear ratio.

2. I am unable to critically assess the quality of the FAI, but I do see a potential problem here too in the competition between exogenous GFP-UBF and endogenous UBF. Changes in the FAI could indicate changes in the ratio of bound to excess GFP-UBF, which correlate with changes in competition with endogenous UBF but do not reflect changes in endogenous UBF function.

The potential pitfalls associated with the use of GFP-UBF overexpression could be mentioned in the Discussion.

3. The legend of Supp Figure EV5 contains „(B) pHrodo-AM incubated in U2OS cells (1:1000) for 30 minutes (C) Nucleolar intensity of pHrodo-AM computed over n>=43 nucleoli. Data represents mean {plus minus} S.E.M, with significance tested with K.S.test (*** represents p<= 0.0001). Scale bar is 1µm.", which should be deleted. (It is from Appendix 3.)

4. Souvik Sen's name is missing from the list of authors in the Supplementary Information.

Review response – Ghosh et al

We sincerely thank the editor and reviewers for their thoughtful evaluation of our manuscript. The feedback has helped us improve both the clarity and technical depth of our work. We have addressed the comments below, with author responses highlighted in **blue**.

Editor's comments:

The sentence in the Data Availability Section regarding the Source Data should be removed and instead this sentence: "This study includes no data deposited in external repositories" should be provided.

The 'conflict of interest' subheading needs to be corrected to "Disclosure and Competing Interests Statement" (DCIS). Employment in a biotech company should be stated in the DCIS.

All supplementary figures can be provided as Expanded View (EV) figures that need to be uploaded as individual, production quality figure files. It is fine to have 8 EV figures. The EV figure legends need to be provided after the main figure legends in the main ms file; the correct nomenclature is Figure EV1, etc. Please correct all file names and the callouts to the EV figures.

A callout is missing for Figure 7, please add.

The Reagents & Tools TABLE needs to be removed from the ms and uploaded as separate file using the same (Reagents table) file type; callouts to the tables need to be corrected. All tables in the ms file should be part of the Reagents and Tools table.

The Source Data need to be uploaded as one file per main figure and the readme file can be added to the Figure 5 folder.

Number of words/figures/ references, etc. should be removed from the title page

Materials and Methods should be Methods

* Figure Legends - Comments *

- Please note that the exact p values are not provided in the legends of figures 4B,C,D,E,F; 5D,E; 6C,D; EV-1A; EV-3B,F,G,H,J,K; EV-4B,C,F,G; EV-5D; Appendix Figure 3B. Pease provide exact values as reasonable.
- Please note that for the figure 3, p-values and statistical tests are indicated in the legends. However, comparison for the same, ""****/n.s"" has not been represented in the figures. Please rectify this in the figures or legends as applicable.
- Please note that information related to n is missing in the legends of Appendix Figure 3B.
- Please note that the scale bar is missing for figures 2B,C.
- Please note that the scale bar needs to be defined for figures EV-2A,B,C; EV-3A,D,E.
- Please note that in legends of figures EV-4A,D the scale bar unit should be corrected from µM to µm.

I would like to suggest some minor changes to the title and abstract that needs to be written in present tense. Do you agree with this:

Nucleolar reorganization upon stress depends on physicochemical changes due to reduced nascent rRNA synthesis [OK?]

Successive maturation of ribosomal subunits occurs through multilayered phase-separated structures of the cell nucleolus. The spatio-functional relationship between transcription of rRNA and nucleolar substructures, and how this adapts to cellular stress remain incompletely understood. In this study, we resolve the sub nucleolar structures using expansion microscopy to reveal ordered structures of fibrillar center (FC) and dense fibrillar component (DFC) domains as nested shells, which are reorganized upon cellular stress like DNA damage or RNA polymerase I (RNAPI) inhibition. Direct visualization of nascent (5' ETS) and mature (28S or 18S) rRNA suggests that rRNA synthesis is a critical regulator of nucleolar size and organization. Nucleolar reorganization upon stress emerges to be a direct function of nascent rRNA levels. Stress-induced transcription inhibition can remodel the sub-nucleolar compartments to a low mobility state and perturbs the nucleolar pH gradient due to the missing rRNA scaffold and other factors. We show that rather than signaling to mediate rDNA repair, nucleolar reorganization arises naturally from reduced rRNA levels and the resultant biophysical restructuring of the nucleolus under cellular stress.

Author response: We thank the editor for their careful evaluation and positive comments on the manuscript. We have now addressed all of these in our current version of the manuscript.

EMBO press papers are accompanied online by A) a short (1-2 sentences) summary of the findings and their significance, B) 2-3 bullet points highlighting key results and C) a synopsis image that is exactly 550 pixels wide and 300 pixels high. The synopsis image should provide a sketch of the major findings, like a graphical abstract. Please note that text needs to be readable at the final size. Please send us this information along with the final manuscript.

Author response: We have now added this to a new document named “Summary and Graphical Abstract”.

Referee #1:

The authors have addressed most of the previous questions, and the manuscript has been substantially improved. Before publication, a couple of minor concerns still require clarification: the nucleolar morphology after 1 h of 4-NQO treatment appears to be inconsistent between Figure 2b and Figure 5a. In Figure 2b, the nested FC/DFC structure has been disrupted, whereas in Figure 5a the structure appears largely unaffected. The authors should carefully verify these data and provide a critical assessment of the actual effects of 4-NQO treatment on nucleolar organization. Also, in Figure 5a, the UBF signals are overexposed, making it difficult to accurately evaluate their localization and morphology. Please adjust the image display parameters to better reflect the original fluorescence signal.

Overall, provided that the above concern is satisfactorily addressed, no further concerns remain regarding publication.

Author response: We thank the reviewer for their careful evaluation and positive comments on the manuscript. We have now modified the figures to address these points. In Figure 5A also there was reorganization like in Figure 2B, but perhaps that was not clear as the nucleolar boundary was not very clear in the images presented. To overcome this, we have now indicated the approximate nucleolar boundaries with a white dotted line in 5A merge column. As is now apparent there is reorganization here too. Further as we have shown, the nucleoli become smaller and UBF foci coalesce with DNA damage or transcriptional inhibition, increasing the apparent intensity of individual foci. At the same contrast settings as control nucleoli this can give the impression of saturation, but we have ensured that there is no saturation in the images during acquisition. To address the point brought up by the reviewer, we have now presented images of nucleoli which do not give the impression of apparent overexposure.

Referee #2:

This study uses expansion microscopy to understand how nucleolar organization is influenced during rDNA transcription downregulation and/or exposure to rDNA-damaging agents, with a specific emphasis on how rRNA levels alters the biophysical properties of the nucleolus, namely its viscoelasticity and pH. The authors have largely addressed my previous concerns, and I have no additional concerns.

I will reiterate that, although some of the data presented here confirm and only marginally expand our current understanding of how the nucleolus reorganizes under different conditions, this study is nonetheless important and significant, and I therefore support its publication in EMBO. This work will be well appreciated by the field in light of emerging mechanisms surrounding nucleolar biophysical homeostasis, function, and architecture.

Author response: We thank the reviewer for their careful evaluation and positive comments on our manuscript.

Referee #3:

The authors have carefully addressed all my comments and present a considerable number of new experiments to further substantiate and expand upon their findings. These include pHrodo-AM, yH2AX, 18S and PARP1 analyses, the translocation of DE-tract proteins, NCS and CPT treatments, and fluorescence anisotropy imaging (FAI) of GFP-UBF to investigate local order, viscosity and rotational constraints. In addition, they implemented extensive amendments to the text in line with the suggestions and criticisms. This impressive effort, undertaken within the short timeframe of the peer-review process, is greatly appreciated.

Although the novelty of the study is still limited, the revised, extended version complements

earlier findings well. The revised version also refers to these findings more appropriately and presents them more clearly. In my opinion, the use of GFP-UBF still represents a limitation, despite the described selection of cells with comparable expression levels; however, the study's main conclusions do not depend exclusively on the GFP-UBF analyses, so this limitation is acceptable.

Minor points

1. In the PARP1 analysis (Supplementary Figure EV5), nucleoli are presumably identified as described in Appendices 1 and 2. The risk associated with using Hoechst exclusion staining as a substitute for nucleolar staining is that nuclear bodies (Cajal bodies, nuclear spots, paraspeckles) are also only weakly stained and cannot be distinguished from smaller nucleoli. The cell nucleus shown in Appendix 2 illustrates this problem very well. It would be useful to specify the nucleolus selection criteria (size?) and briefly discuss this point in the 'Methods' section, as it is crucial for calculating the nucleolar/nuclear ratio.

2. I am unable to critically assess the quality of the FAI, but I do see a potential problem here too in the competition between exogenous GFP-UBF and endogenous UBF. Changes in the FAI could indicate changes in the ratio of bound to excess GFP-UBF, which correlate with changes in competition with endogenous UBF but do not reflect changes in endogenous UBF function. The potential pitfalls associated with the use of GFP-UBF overexpression could be mentioned in the Discussion.

3. The legend of Supp Figure EV5 contains „(B) pHrodo-AM incubated in U2OS cells (1:1000) for 30 minutes (C) Nucleolar intensity of pHrodo-AM computed over n>=43 nucleoli. Data represents mean {plus minus} S.E.M, with significance tested with K.S.test (*** represents p<= 0.0001). Scale bar is 1µm.", which should be deleted. (It is from Appendix 3.)

4. Souvik Sen's name is missing from the list of authors in the Supplementary Information.

Author response: We thank the reviewer for their careful evaluation and positive comments on our manuscript. We have now addressed all of these in our revised manuscript.

3rd Revision - Editorial Decision **31st Aug 2026**

Prof. Aprotim Mazumder
Tata Institute of Fundamental Research (TIFR) Hyderabad
TIFR Centre for Interdisciplinary Sciences
36/P, Gopanpally
Serilingampally Mandal
Hyderabad, Telangana 500046
India

Dear Prof. Mazumder,

I am very pleased to accept your manuscript for publication in the next available issue of EMBO reports. Thank you for your contribution to our journal.

Your manuscript will be processed for publication by EMBO Press. It will be copy edited and you will receive page proofs prior to publication. Please note that you will be contacted by Springer Nature Author Services to complete licensing and payment information.

You may qualify for financial assistance for your publication charges - either via a Springer Nature fully open access agreement or an EMBO initiative. Check your eligibility: https://link.springer.com/journal/44319/how-to-publish-with-us

Should you be planning a Press Release on your article, please notify the production team via this form in order to coordinate publication and release dates: https://forms.cloud.microsoft/e/eH4A8JVP6g

If you have any questions, please do not hesitate to contact the Editorial Office. Thank you for your contribution to EMBO Reports.

Best regards,
Esther

Esther Schnapp, PhD
Senior Editor
EMBO reports

>>> Please note that it is EMBO Reports policy for the transcript of the editorial process (containing referee reports and your response letter) to be published as an online supplement to each paper. If you do NOT want this, you will need to inform the Editorial Office via email immediately. More information is available here: https://link.springer.com/partners/embo-press/editorial-policies#Peer%20review

EMBO Press Author Checklist

Corresponding Author Name: Aprotim Mazumder	
Journal Submitted to: EMBO Reports	
Manuscript Number: EMBOR-2025-63402V4	

USEFUL LINKS FOR COMPLETING THIS FORM
The EMBO Journal - Author Guidelines
EMBO Reports - Author Guidelines
Molecular Systems Biology - Author Guidelines
EMBO Molecular Medicine - Author Guidelines

Reporting Checklist for Life Science Articles (updated January
This checklist is adapted from Materials Design Analysis Reporting (MDAR) Checklist for Authors. MDAR establishes a minimum set of requirements in transparent reporting in the life sciences (see Statement of Task: 10.31222/osf.io/9sm4x). Please follow the journal's guidelines in preparing your manuscript.
Please note that a copy of this checklist will be published alongside your article.

Abridged guidelines for figures
1. Data
The data shown in figures should satisfy the following conditions:
- the data were obtained and processed according to the field's best practice and are presented to reflect the results of the experiments in an accurate and unbiased manner.
- ideally, figure panels should include only measurements that are directly comparable to each other and obtained with the same assay.
- plots include clearly labeled error bars for independent experiments and sample sizes. Unless justified, error bars should not be shown for technical
- if n<5, the individual data points from each experiment should be plotted. Any statistical test employed should be justified.
- Source Data should be included to report the data underlying figures according to the guidelines set out in the authorship guidelines on Data

2. Captions
Each figure caption should contain the following information, for each panel where they are relevant:
- a specification of the experimental system investigated (eg cell line, species name).
- the assay(s) and method(s) used to carry out the reported observations and measurements.
- an explicit mention of the biological and chemical entity(ies) that are being measured.
- an explicit mention of the biological and chemical entity(ies) that are altered/varied/perturbed in a controlled manner.
- the exact sample size (n) for each experimental group/condition, given as a number, not a range;
- a description of the sample collection allowing the reader to understand whether the samples represent technical or biological replicates (including how many animals, litters, cultures, etc.).
- a statement of how many times the experiment shown was independently replicated in the laboratory.
- definitions of statistical methods and measures:
  - common tests, such as t-test (please specify whether paired vs. unpaired), simple $\chi 2$ tests, Wilcoxon and Mann-Whitney tests, can be unambiguously identified by name only, but more complex techniques should be described in the methods section;
  - are tests one-sided or two-sided?
  - are there adjustments for multiple comparisons?
  - exact statistical test results, e.g., P values = x but not P values < x;
  - definition of 'center values' as median or average;
  - definition of error bars as s.d. or s.e.m.

Please complete ALL of the questions below.
Select "Not Applicable" only when the requested information is not relevant for your study.

Materials

Newly Created Materials	Information included in the manuscript?	In which section is the information available? (Reagents and Tools Table, Materials and Methods, Figures, Data Availability Section)
New materials and reagents need to be available; do any restrictions apply?	Not Applicable	

Antibodies	Information included in the manuscript?	In which section is the information available? (Reagents and Tools Table, Materials and Methods, Figures, Data Availability Section)
For **antibodies** provide the following information:<br>- Commercial antibodies: RRID (if possible) or supplier name, catalogue number and or/clone number<br>- Non-commercial: RRID or citation	Yes	Materials and methods

DNA and RNA sequences	Information included in the manuscript?	In which section is the information available? (Reagents and Tools Table, Materials and Methods, Figures, Data Availability Section)
**Short novel DNA or RNA including primers, probes**: provide the sequences.	Yes	Materials and methods- table

Cell materials	Information included in the manuscript?	In which section is the information available? (Reagents and Tools Table, Materials and Methods, Figures, Data Availability Section)
**Cell lines:** Provide species information, strain. Provide accession number in repository **OR** supplier name, catalog number, clone number, and/**OR** RRID.	Yes	Figures, Materials and methods
**Primary cultures:** Provide species, strain, sex of origin, genetic modification status.	Not Applicable	
Report if the cell lines were recently **authenticated** (e.g., by STR profiling) and tested for mycoplasma contamination.	Not Applicable	

Experimental animals	Information included in the manuscript?	In which section is the information available? (Reagents and Tools Table, Materials and Methods, Figures, Data Availability Section)
**Laboratory animals or Model organisms:** Provide species, strain, sex, age, genetic modification status. Provide accession number in repository **OR** supplier name, catalog number, clone number, **OR** RRID.	Not Applicable	
**Animal observed in or captured from the field:** Provide species, sex, and age where possible.	Not Applicable	
Please detail **housing and husbandry conditions**.	Not Applicable	

Plants and microbes	Information included in the manuscript?	In which section is the information available? (Reagents and Tools Table, Materials and Methods, Figures, Data Availability Section)
**Plants:** provide species and strain, ecotype and cultivar where relevant, unique accession number if available, and source (including location for collected wild specimens).	Not Applicable	
**Microbes:** provide species and strain, unique accession number if available, and source.	Not Applicable	

Human research participants	Information included in the manuscript?	In which section is the information available? (Reagents and Tools Table, Materials and Methods, Figures, Data Availability Section)
If collected and within the bounds of privacy constraints report on age, sex and gender or ethnicity for all study participants.	Not Applicable	

Core facilities	Information included in the manuscript?	In which section is the information available? (Reagents and Tools Table, Materials and Methods, Figures, Data Availability Section)
If your work benefited from core facilities, was their service mentioned in the acknowledgements section?	Yes	Acknowledgements

Design

Study protocol	Information included in the manuscript?	In which section is the information available? (Reagents and Tools Table, Materials and Methods, Figures, Data Availability Section)
If study protocol has been **pre-registered, provide DOI in the manuscript**. For clinical trials, provide the trial registration number **OR** cite DOI.	Not Applicable	
Report the **clinical trial registration number** (at ClinicalTrials.gov or equivalent), where applicable.	Not Applicable	

Laboratory protocol	Information included in the manuscript?	In which section is the information available? (Reagents and Tools Table, Materials and Methods, Figures, Data Availability Section)
Provide DOI OR other citation details if **external detailed step-by-step protocols** are available.	Yes	Materials and methods

Experimental study design and statistics	Information included in the manuscript?	In which section is the information available? (Reagents and Tools Table, Materials and Methods, Figures, Data Availability Section)
Include a statement about **sample size** estimate even if no statistical methods were used.	Yes	Figure legends
Were any steps taken to minimize the effects of subjective bias when allocating animals/samples to treatment (e.g. **randomization procedure**)? If yes, have they been described?	Not Applicable	
Include a statement about **blinding** even if no blinding was done.	Not Applicable	
Describe **inclusion/exclusion criteria** if samples or animals were excluded from the analysis. Were the criteria pre-established? If sample or data points were omitted from analysis, report if this was due to attrition or intentional exclusion and provide justification.	Yes	Materials and methods
For every figure, are **statistical tests** justified as appropriate? Do the data meet the assumptions of the tests (e.g., normal distribution)? Describe any methods used to assess it. Is there an estimate of variation within each group of data? Is the variance similar between the groups that are being statistically compared?	Yes	Figure legends, Materials and methods

Sample definition and in-laboratory replication	Information included in the manuscript?	In which section is the information available? (Reagents and Tools Table, Materials and Methods, Figures, Data Availability Section)
In the figure legends: state number of times the experiment was **replicated** in laboratory.	Yes	Figure legends
In the figure legends: define whether data describe **technical or biological replicates**.	Yes	Figure legends

Ethics

Ethics	Information included in the manuscript?	In which section is the information available? (Reagents and Tools Table, Materials and Methods, Figures, Data Availability Section)
Studies involving **human participants**: State details of **authority granting ethics approval** (IRB or equivalent committee(s), provide reference number for approval.	Not Applicable	
Studies involving **human participants**: Include a statement confirming that **informed consent** was obtained from all subjects and that the experiments conformed to the principles set out in the WMA Declaration of Helsinki and the Department of Health and Human Services Belmont Report.	Not Applicable	
Studies involving **human participants:** For publication of **patient photos**, include a statement confirming that consent to publish was obtained.	Not Applicable	
Studies involving experimental **animals**: State details of **authority granting ethics approva**l (IRB or equivalent committee(s), provide reference number for approval. Include a statement of compliance with ethical regulations.	Not Applicable	
Studies involving **specimen and field samples:** State if relevant **permits** obtained, provide details of authority approving study; if none were required, explain why.	Not Applicable	

Dual Use Research of Concern (DURC)	Information included in the manuscript?	In which section is the information available? (Reagents and Tools Table, Materials and Methods, Figures, Data Availability Section)
Could your study fall under dual use research restrictions? Please check biosecurity documents and list of **select agents and toxins** (CDC): https://www.selectagents.gov/sat/list.htm	Not Applicable	
If you used a select agent, is the security level of the lab appropriate and reported in the manuscript?	Not Applicable	
If a study is subject to dual use research of concern regulations, is the name of the **authority granting approval and reference number** for the regulatory approval provided in the manuscript?	Not Applicable	

Reporting

The MDAR framework recommends adoption of discipline-specific guidelines, established and endorsed through community initiatives. Journals have their own policy about requiring specific guidelines and recommendations to complement MDAR.

Adherence to community standards	Information included in the manuscript?	In which section is the information available? (Reagents and Tools Table, Materials and Methods, Figures, Data Availability Section)
State if relevant guidelines or checklists (e.g., **ICMJE, MIBBI, ARRIVE, PRISMA**) have been followed or provided.	Not Applicable	
For **tumor marker prognostic studies,** we recommend that you follow the **REMARK** reporting guidelines (see link list at top right). See author guidelines, under 'Reporting Guidelines'. Please confirm you have followed these guidelines.	Not Applicable	
For **phase II and III randomized controlled trials**, please refer to the **CONSORT** flow diagram (see link list at top right) and submit the CONSORT checklist (see link list at top right) with your submission. See author guidelines, under 'Reporting Guidelines'. Please confirm you have submitted this list.	Not Applicable	

Data Availability

Data availability	Information included in the manuscript?	In which section is the information available? (Reagents and Tools Table, Materials and Methods, Figures, Data Availability Section)
Have **primary datasets** been deposited according to the journal's guidelines (see 'Data Deposition' section) and the respective accession numbers provided in the Data Availability Section?	Yes	Data Availability
Were **human clinical and genomic datasets** deposited in a public access-controlled repository in accordance to ethical obligations to the patients and to the applicable consent agreement?	Not Applicable	
Are **computational models** that are central and integral to a study available without restrictions in a machine-readable form? Were the relevant accession numbers or links provided?	Not Applicable	
If publicly available data were reused, provide the respective **data citations in the reference list**.	Not Applicable	
