# Supplementary material for: Nucleolar reorganization on stress depends on physicochemical changes due to reduced nascent rRNA synthesis
Source: EMBO Rep. 2026 Sep 22;27(21):6305–41. doi: 10.1038/s44319-026-00932-z (PMC13743745; doi:10.1038/s44319-026-00932-z)
Supplement: Supplementary file 4 [file 44319_2026_932_MOESM10_ESM.zip › Figure_EV4/EV4 README.docx]

**EV4A**

| **Scatter plot** | Control | 4-NQO | BMH21 |
| --- | --- | --- | --- |
| **X axis** | Column E of sheet ‘Exp 1 EV4A-B-C’, ‘Exp 2 EV4A-B-C’,  ‘Exp 3 EV4A-B-C’ | Column K of sheet ‘Exp 1 EV4A-B-C’, ‘Exp 2 EV4A-B-C’,  ‘Exp 3 EV4A-B-C’ | Column Q of sheet ‘Exp 1 EV4A-B-C’, ‘Exp 2 EV4A-B-C’,  ‘Exp 3 EV4A-B-C’ |
| **Y axis** | Column F of sheet ‘Exp 1 EV4A-B-C’, ‘Exp 2 EV4A-B-C’,  ‘Exp 3 EV4A-B-C’ | Column L of sheet ‘Exp 1 EV4A-B-C’, ‘Exp 2 EV4A-B-C’,  ‘Exp 3 EV4A-B-C’ | Column R of sheet ‘Exp 1 EV4A-B-C’, ‘Exp 2 EV4A-B-C’,  ‘Exp 3 EV4A-B-C’ |

**EV4B**

| **X axis (category)** | Control | 4-NQO | BMH21 |
| --- | --- | --- | --- |
| **Y axis** | Column F of sheet ‘Exp 1 EV4A-B-C’, ‘Exp 2 EV4A-B-C’,  ‘Exp 3 EV4A-B-C’ | Column L of sheet ‘Exp 1 EV4A-B-C’, ‘Exp 2 EV4A-B-C’,  ‘Exp 3 EV4A-B-C’ | Column R of sheet ‘Exp 1 EV4A-B-C’, ‘Exp 2 EV4A-B-C’,  ‘Exp 3 EV4A-B-C’ |

**EV4C**

| **X axis (category)** | Control | 4-NQO | BMH21 |
| --- | --- | --- | --- |
| **Y axis** | Column E of sheet ‘Exp 1 EV4A-B-C’, ‘Exp 2 EV4A-B-C’,  ‘Exp 3 EV4A-B-C’ | Column K of sheet ‘Exp 1 EV4A-B-C’, ‘Exp 2 EV4A-B-C’,  ‘Exp 3 EV4A-B-C’ | Column Q of sheet ‘Exp 1 EV4A-B-C’, ‘Exp 2 EV4A-B-C’,  ‘Exp 3 EV4A-B-C’ |

**EV4E**

| **Scatter plot** | Control | CPT | NCS |
| --- | --- | --- | --- |
| **X axis** | Column C of sheet ‘Exp 1 EV4E-F-G Control’, ‘Exp 2 EV4E-F-G Control’,  ‘Exp 3 EV4E-F-G Control’ | Column C of sheet ‘Exp 1 EV4E-F-G CPT’, ‘Exp 2 EV4E-F-G CPT’,  ‘Exp 3 EV4E-F-G CPT’ | Column C of sheet ‘Exp 1 EV4E-F-G NCS’, ‘Exp 2 EV4E-F-G NCS’,  ‘Exp 3 EV4E-F-G NCS’ |
| **Y axis** | Column F of sheet ‘Exp 1 EV4E-F-G Control’, ‘Exp 2 EV4E-F-G Control’,  ‘Exp 3 EV4E-F-G Control’ | Column F of sheet ‘Exp 1 EV4E-F-G CPT’, ‘Exp 2 EV4E-F-G CPT’,  ‘Exp 3 EV4E-F-G CPT’ | Column F of sheet ‘Exp 1 EV4E-F-G NCS’, ‘Exp 2 EV4E-F-G NCS’,  ‘Exp 3 EV4E-F-G NCS’ |

**EV4F**

| **X axis (category)** | Control | CPT | NCS |
| --- | --- | --- | --- |
| **Y axis** | Column F of sheet ‘Exp 1 EV4E-F-G Control’, ‘Exp 2 EV4E-F-G Control’,  ‘Exp 3 EV4E-F-G Control’ | Column F of sheet ‘Exp 1 EV4E-F-G CPT’, ‘Exp 2 EV4E-F-G CPT’,  ‘Exp 3 EV4E-F-G CPT’ | Column F of sheet ‘Exp 1 EV4E-F-G NCS’, ‘Exp 2 EV4E-F-G NCS’,  ‘Exp 3 EV4E-F-G NCS’ |

**EV4G**

| **X axis (category)** | Control | CPT | NCS |
| --- | --- | --- | --- |
| **Y axis** | Column C of sheet ‘Exp 1 EV4E-F-G Control’, ‘Exp 2 EV4E-F-G Control’,  ‘Exp 3 EV4E-F-G Control’ | Column C of sheet ‘Exp 1 EV4E-F-G CPT’, ‘Exp 2 EV4E-F-G CPT’,  ‘Exp 3 EV4E-F-G CPT’ | Column C of sheet ‘Exp 1 EV4E-F-G NCS’, ‘Exp 2 EV4E-F-G NCS’,  ‘Exp 3 EV4E-F-G NCS’ |
