# Supplementary material for: Nucleolar reorganization on stress depends on physicochemical changes due to reduced nascent rRNA synthesis
Source: EMBO Rep. 2026 Sep 22;27(21):6305–41. doi: 10.1038/s44319-026-00932-z (PMC13743745; doi:10.1038/s44319-026-00932-z)
Supplement: Supplementary file 5 [file 44319_2026_932_MOESM11_ESM.zip › Figure_EV5/EV5 README.docx]

**EV5B**

| **X axis (category)** | Control | 4-NQO | BMH21 |
| --- | --- | --- | --- |
| **Y axis** | Column J of sheet ‘EV5B Pulled Data’ | Column L of sheet ‘EV5B Pulled Data’ | Column N of sheet ‘EV5B Pulled Data’ |

**EV5D**

| **X axis (category)** | Control | 4-NQO | BMH21 |
| --- | --- | --- | --- |
| **Y axis** | Column C of sheet ‘Figure EV5D’ | Column D of sheet ‘Figure EV5D’ | Column E of sheet ‘Figure EV5D’ |

**EV5E**

| **X axis** | Column B of sheet ‘Figure EV5E’ |
| --- | --- |
| **Y axis** | Column C of sheet ‘Figure EV5E’ |
