# Supplementary material for: Nucleolar reorganization on stress depends on physicochemical changes due to reduced nascent rRNA synthesis
Source: EMBO Rep. 2026 Sep 22;27(21):6305–41. doi: 10.1038/s44319-026-00932-z (PMC13743745; doi:10.1038/s44319-026-00932-z)
Supplement: Supplementary file 6 [file 44319_2026_932_MOESM12_ESM.zip › Appendix figure S3/Appendix figure_3 README.docx]

**Appendix figure 3B**

| **X axis (category)** | Control | 4-NQO | BMH21 |
| --- | --- | --- | --- |
| **Y axis** | Column C | Column E | ColumnG |
