# Supplementary material for: Nucleolar reorganization on stress depends on physicochemical changes due to reduced nascent rRNA synthesis
Source: EMBO Rep. 2026 Sep 22;27(21):6305–41. doi: 10.1038/s44319-026-00932-z (PMC13743745; doi:10.1038/s44319-026-00932-z)
Supplement: Supplementary file 7 [file 44319_2026_932_MOESM3_ESM.zip › Figure_1/Figure_1 README.docx]

**Figure 1C**

| **X axis** | Column B from sheet ‘Figure 1C’ |
| --- | --- |
| **Y axis** | Column C from sheet ‘Figure 1C’ |

**Figure 1D**

| **X axis** | Column B from sheet ‘Figure 1D’ |
| --- | --- |
| **Y axis** | Column C from sheet ‘Figure 1D’ |

**Figure 1F**

| **X axis** | Column C from sheet ‘Figure 1F’ |
| --- | --- |
| **Y axis** | Column J, K and L from sheet ‘Figure 1F’ |

**Figure 1G**

| **X axis** | Column A from sheet ‘Figure 1G’ |
| --- | --- |
| **Y axis** | Column P, AG from sheet ‘Figure 1G’ |
