# Supplementary material for: Nucleolar reorganization on stress depends on physicochemical changes due to reduced nascent rRNA synthesis
Source: EMBO Rep. 2026 Sep 22;27(21):6305–41. doi: 10.1038/s44319-026-00932-z (PMC13743745; doi:10.1038/s44319-026-00932-z)
Supplement: Supplementary file 8 [file 44319_2026_932_MOESM4_ESM.zip › Figure_3/Figure_3 README.docx]

**Figure 3C**

| **X axis** | Column B from sheet ‘Figure 3C’ |
| --- | --- |
| **Y axis** | Column D, H and L from sheet ‘Figure 3C’ |

**Figure 3D**

| **X axis (category)** | UBF | rRNA |
| --- | --- | --- |
| **Y axis** | Column E, M and U of sheet ‘Figure 3D’ | Column F, N and V of sheet ‘Figure 3D’ |

**Figure 3E**

| **X axis** | Column B from sheet ‘Figure 3E’ |
| --- | --- |
| **Y axis** | Column D, H and L from sheet ‘Figure 3E’ |
