# Supplementary material for: Nucleolar reorganization on stress depends on physicochemical changes due to reduced nascent rRNA synthesis
Source: EMBO Rep. 2026 Sep 22;27(21):6305–41. doi: 10.1038/s44319-026-00932-z (PMC13743745; doi:10.1038/s44319-026-00932-z)
Supplement: Supplementary file 9 [file 44319_2026_932_MOESM5_ESM.zip › Figure_4/Figure_4 README.docx]

**Figure 4A**

|  | Control | 4-NQO | BMH21 |
| --- | --- | --- | --- |
| **X axis** | Column C of sheet ‘Figure 4A’ | Column F of sheet ‘Figure 4A’ | Column I of sheet ‘Figure 4A’ |
| **Y axis** | Column B of sheet ‘Figure 4A’ | Column E of sheet ‘Figure 4A’ | Column H of sheet ‘Figure 4A’ |

**Figure 4B**

| **X axis (category)** | Control | 4-NQO | BMH21 |
| --- | --- | --- | --- |
| **Y axis** | Column B of sheet ‘Figure 4B’ | Column C of sheet ‘Figure 4B’ | Column D of sheet ‘Figure 4B’ |

**Figure 4C**

| **X axis (category)** | Control | 4-NQO | BMH21 |
| --- | --- | --- | --- |
| **Y axis** | Column B of sheet ‘Figure 4C’ | Column C of sheet ‘Figure 4C’ | Column D of sheet ‘Figure 4C’ |

Data from figure 4A has been presented as bar graphs in 4B and 4C

**Figure 4D**

| **X axis (category)** | Control | 4-NQO | BMH21 |
| --- | --- | --- | --- |
| **Y axis** | Column C of sheet ‘Figure 4D-E-F-G’ | Column D of sheet ‘Figure 4D-E-F-G’ | Column E of sheet ‘Figure 4D-E-F-G’ |

**Figure 4E**

| **X axis (category)** | Control | 4-NQO | BMH21 |
| --- | --- | --- | --- |
| **Y axis** | Column O of sheet ‘Figure 4D-E-F-G’ | Column P of sheet ‘Figure 4D-E-F-G’ | Column Q of sheet ‘Figure 4D-E-F-G’ |

**Figure 4F**

| **X axis (category)** | Control | 4-NQO | BMH21 |
| --- | --- | --- | --- |
| **Y axis** | Column U of sheet ‘Figure 4D-E-F-G’ | Column V of sheet ‘Figure 4D-E-F-G’ | Column W of sheet ‘Figure 4D-E-F-G’ |

**Figure 4G**

| **X axis (category)** | Control | 4-NQO | BMH21 |
| --- | --- | --- | --- |
| **Y axis** | Column I of sheet ‘Figure 4D-E-F-G’ | Column J of sheet ‘Figure 4D-E-F-G’ | Column K of sheet ‘Figure 4D-E-F-G’ |
