# Supplementary material for: Nucleolar reorganization on stress depends on physicochemical changes due to reduced nascent rRNA synthesis
Source: EMBO Rep. 2026 Sep 22;27(21):6305–41. doi: 10.1038/s44319-026-00932-z (PMC13743745; doi:10.1038/s44319-026-00932-z)
Supplement: Supplementary file 10 [file 44319_2026_932_MOESM6_ESM.zip › Figure_5/Figure_5 README.docx]

**Figure 5B**

| **Scatter plot** | Control | 4-NQO 1h | 4-NQO 3h | BMH21 |
| --- | --- | --- | --- | --- |
| **X axis** | Column H of sheets  ‘Figure 5B-D-E-Exp 1 Control’, ‘Figure 5B-D-E-Exp 2 Control’, and ‘Figure 5B-D-E-Exp 3 Control’ | Column H of sheets ‘Figure 5B-D-E-Exp 1 4NQO-1h’, ‘Figure 5B-D-E-Exp 2 4NQO-1h’, and ‘Figure 5B-D-E-Exp 3 4NQO-1h’ | Column H of sheets ‘Figure 5B-D-E-Exp 1 4NQO-13h’, ‘Figure 5B-D-E-Exp 2 4NQO-3h’, and ‘Figure 5B-D-E-Exp 3 4NQO-3h’ | Column H of sheets ‘Figure 5B-D-E-Exp 1 BMH21’, ‘Figure 5B-D-E-Exp 2 BMH21’, and ‘Figure 5B-D-E-Exp 3 BMH21’ |
| **Y axis** | Column E of sheets ‘Figure 5B-D-E-Exp 1 Control’, ‘Figure 5B-D-E-Exp 2 Control’, and ‘Figure 5B-D-E-Exp 3 Control’ | Column E of sheets ‘Figure 5B-D-E-Exp 1 4NQO-1h’, ‘Figure 5B-D-E-Exp 2 4NQO-1h’, and ‘Figure 5B-D-E-Exp 3 4NQO-1h’ | Column E of sheets ‘Figure 5B-D-E-Exp 1 4NQO-13h’, ‘Figure 5B-D-E-Exp 2 4NQO-3h’, and ‘Figure 5B-D-E-Exp 3 4NQO-3h’ | Column E of sheets ‘Figure 5B-D-E-Exp 1 BMH21’, ‘Figure 5B-D-E-Exp 2 BMH21’, and ‘Figure 5B-D-E-Exp 3 BMH21’ |

**Figure 5C**

| **X axis** | Column C of sheet ‘Figure 5C’ |
| --- | --- |
| **Y axis** | Column D, G, J and M of sheet ’Figure 5C’ |

**Figure 5D**

| **X axis** | Control | 4-NQO 1h | 4-NQO 3h | BMH21 |
| --- | --- | --- | --- | --- |
| **Y axis** | Column H of sheets ‘Figure 5B-D-E-Exp 1 Control’, ‘Figure 5B-D-E-Exp 2 Control’, and ‘Figure 5B-D-E-Exp 3 Control’ | Column H of sheets ‘Figure 5B-D-E-Exp 1 4NQO-1h’, ‘Figure 5B-D-E-Exp 2 4NQO-1h’, and ‘Figure 5B-D-E-Exp 3 4NQO-1h’ | Column H of sheets ‘Figure 5B-D-E-Exp 1 4NQO-13h’, ‘Figure 5B-D-E-Exp 2 4NQO-3h’, and ‘Figure 5B-D-E-Exp 3 4NQO-3h’ | Column H of sheets ‘Figure 5B-D-E-Exp 1 BMH21’, ‘Figure 5B-D-E-Exp 2 BMH21’, and ‘Figure 5B-D-E-Exp 3 BMH21’ |

**Figure 5E**

| **X axis** | Control | 4-NQO 1h | 4-NQO 3h | BMH21 |
| --- | --- | --- | --- | --- |
| **Y axis** | Column E of sheets ‘Figure 5B-D-E-Exp 1 Control’, ‘Figure 5B-D-E-Exp 2 Control’, and ‘Figure 5B-D-E-Exp 3 Control’ | Column E of sheets ‘Figure 5B-D-E-Exp 1 4NQO-1h’, ‘Figure 5B-D-E-Exp 2 4NQO-1h’, and ‘Figure 5B-D-E-Exp 3 4NQO-1h’ | Column E of sheets ‘Figure 5B-D-E-Exp 1 4NQO-13h’, ‘Figure 5B-D-E-Exp 2 4NQO-3h’, and ‘Figure 5B-D-E-Exp 3 4NQO-3h’ | Column E of sheets ‘Figure 5B-D-E-Exp 1 BMH21’, ‘Figure 5B-D-E-Exp 2 BMH21’, and ‘Figure 5B-D-E-Exp 3 BMH21’ |
