# Supplementary material for: Nucleolar reorganization on stress depends on physicochemical changes due to reduced nascent rRNA synthesis
Source: EMBO Rep. 2026 Sep 22;27(21):6305–41. doi: 10.1038/s44319-026-00932-z (PMC13743745; doi:10.1038/s44319-026-00932-z)
Supplement: Supplementary file 11 [file 44319_2026_932_MOESM7_ESM.zip › Figure_6/Figure_6 README.docx]

**Figure 6B**

|  | Control | 4-NQO-1h | 4-NQO-3h | BMH21 |
| --- | --- | --- | --- | --- |
| **X axis** | Column B of sheet ‘Figure 6B-control’ | Column B of sheet ‘Figure 6B-control’ | Column B of sheet ‘Figure 6B-control’ | Column B of sheet ‘Figure 6B-control’ |
| **Y axis** | Column Q of sheet ‘Figure 6B-control’ | Column V of sheet ‘Figure 6B-4NQO-1h’ | Column T of sheet ‘Figure 6B-4NQO-3h’ | Column X of sheet ‘Figure 6B-BMH21’ |

**Figure 6C**

| **X-axis (Category)** | Column B of sheet ‘Figure 6C’ |
| --- | --- |
| **Y axis** | Column C of sheet ‘Figure 6C’ |

**Figure 6D**

| **X axis (Category)** | Control | 4-NQO-1h | BMH21 |
| --- | --- | --- | --- |
| **Y axis** | Column C, G, K of sheet ‘Figure 6D’ | Column D, H, L of sheet ‘Figure 6D’ | Column E, I, M of sheet ‘Figure 6D’ |
