# Supplementary material for: Nucleolar reorganization on stress depends on physicochemical changes due to reduced nascent rRNA synthesis
Source: EMBO Rep. 2026 Sep 22;27(21):6305–41. doi: 10.1038/s44319-026-00932-z (PMC13743745; doi:10.1038/s44319-026-00932-z)
Supplement: Supplementary file 12 [file 44319_2026_932_MOESM8_ESM.zip › Figure_EV1/EV1 README.docx]

**EV1A**

| **X axis (category)** | Unexpanded | Expanded |
| --- | --- | --- |
| **Y axis** | Column C of sheet ‘Figure EV1A’ | Column D of sheet ‘Figure EV1A’ |

**EV1E**

|  | Ki 67-NCL | RNAPI-NCL | NPM1-NCL |
| --- | --- | --- | --- |
| **X axis** | Column A from sheet ‘Figure EV1E Ki-67 NCL’ | Column A from sheet ‘Figure EV1E RNAPI NCL’ | Column A from sheet ‘Figure EV1E NPM1 NCL’ |
| **Y axis** | Column C and F from sheet ‘Figure EV1E Ki-67 NCL’ | Column C and F from sheet ‘Figure EV1E RNAPI NCL’ | Column C and F from sheet ‘Figure EV1E NPM1 NCL’ |
