# Supplementary material for: Nucleolar reorganization on stress depends on physicochemical changes due to reduced nascent rRNA synthesis
Source: EMBO Rep. 2026 Sep 22;27(21):6305–41. doi: 10.1038/s44319-026-00932-z (PMC13743745; doi:10.1038/s44319-026-00932-z)
Supplement: Supplementary file 13 [file 44319_2026_932_MOESM9_ESM.zip › Figure_EV3/EV3 README.docx]

**EV3B**

| **X axis (category)** | Control | 4-NQO | BMH21 |
| --- | --- | --- | --- |
| **Y axis** | Column P of sheet ‘Figure EV3B-C’ | Column Q of sheet ‘Figure EV3B-C’ | Column R of sheet ‘Figure EV3B-C’ |

**EV3C**

| **X axis (category)** | Control | 4-NQO | BMH21 |
| --- | --- | --- | --- |
| **Y axis** | Column L of sheet ‘Figure EV3B-C’ | Column M of sheet ‘Figure EV3B-C’ | Column N of sheet ‘Figure EV3B-C’ |

**EV3F**

| **X axis (category)** | Control | 4-NQO | BMH21 |
| --- | --- | --- | --- |
| **Y axis** | Column L, AD, AV of sheet ‘Figure EV3F-G’ | Column O, AG, AY of sheet ‘Figure EV3F-G’ | Column R, AJ, BB of sheet ‘Figure EV3F-G’ |

**EV3G**

| **X axis (category)** | Control | 4-NQO | BMH21 |
| --- | --- | --- | --- |
| **Y axis** | Column M, AE, AW of sheet ‘Figure EV3F-G’ | Column P, AH, AZ of sheet ‘Figure EV3F-G’ | Column S, AK, BC of sheet ‘Figure EV3F-G’ |

**EV3H**

| **X axis (category)** | Control | 4-NQO | BMH21 |
| --- | --- | --- | --- |
| **Y axis** | Column B of sheet ‘Figure EV3H-K’ | Column C of sheet ‘Figure EV3H-K’ | Column D of sheet ‘Figure EV3H-K’ |

**EV3I**

| **X axis (category)** | Control | 4-NQO | BMH21 |
| --- | --- | --- | --- |
| **Y axis** | Column B, L, V of sheet ‘Figure EV3I-J’ | Column E, O , Y of sheet ‘Figure EV3I-J’ | Column H, R, AB of sheet ‘Figure EV3I-J’ |

**EV3J**

| **X axis (category)** | Control | 4-NQO | BMH21 |
| --- | --- | --- | --- |
| **Y axis** | Column C, M,W of sheet ‘Figure EV3I-J’ | Column F, P, Z of sheet ‘Figure EV3I-J’ | Column I, S, AC of sheet ‘Figure EV3I-J’ |

**EV3K**

| **X axis (category)** | Control | 4-NQO | BMH21 |
| --- | --- | --- | --- |
| **Y axis** | Column I of sheet ‘Figure EV3H-K’ | Column J of sheet ‘Figure EV3H-K’ | Column K of sheet ‘Figure EV3H-K’ |
